# Ultrasound-induced luminescence imaging for deep tissues

**DOI:** 10.7150/thno.133393

**Published:** 2026-09-11

**Authors:** Qiuyu Cheng, Jinlan Luo, Yandi Tan, Tong Xu, Yufeng Sun, Rui Geng, Hanbin Xu, Xiangmin Zhang, Lingwen Ding, Gege Wu, Xinwu Cui, Yijing Liu

**Affiliations:** 1 Department of Medical Ultrasound, Tongji Hospital, Tongji Medical College and State Key Laboratory for Diagnosis and Treatment of Severe Zoonotic Infectious Diseases, Huazhong University of Science and Technology, No. 1095, Jiefang Avenue, Wuhan, Hubei Province, 430030, China.; 2 Hubei Key Laboratory of Bioinorganic Chemistry and Materia Medica; Hubei Engineering Research Center for Biomaterials and Medical Protective Materials; School of Chemistry and Chemical Engineering, Huazhong University of Science and Technology, Wuhan 430074, China.; 3 Department of Radiology, Children's Hospital of Chongqing Medical University, National Clinical Research Center for Child Health and Disorders, Ministry of Education Key Laboratory of Child Development and Disorders, No. 136, Zhongshan Second Road, Chongqing, 400014, China.; 4 Department of Pathology, Yong Loo Lin School of Medicine, National University of Singapore, Singapore, 117597, Singapore.; 5 Nanomedicine Translational Research Program, Yong Loo Lin School of Medicine, National University of Singapore, Singapore, 117597, Singapore.

**Keywords:** ultrasound-induced luminescence, sonoafterglow, mechanoluminescence, ultrasound, deep-tissue imaging

## Abstract

Ultrasound-induced luminescence imaging enables non-invasive and space-constrained luminescent probe activation, thereby overcoming the limited penetration and high background of traditional light-excited imaging. In this review, we summarize recent advances in the area, focusing on three major paradigms: instant ultrasound-induced luminescence, sonoafterglow luminescence, and Off-On switching luminescence, with detailed explanations of their mechanisms. We also explore various strategies to improve luminescent and biological performance. Additionally, we offer a comprehensive overview of biomedical applications in the tumor microenvironment, the immune system, the nervous system and other regions. Moreover, major challenges in clinical translation and prospects are discussed and identified. These advances make ultrasound-induced luminescence a practical route for generating and controlling light in deep tissue, with promise for precise imaging and targeted treatment of deep-seated disease.

## Introduction

Achieving high-resolution visualization within deep biological tissues remains a fundamental challenge in bioimaging. While optical imaging has been central to molecular diagnostics owing to its high sensitivity and specificity 1-5, it is ultimately constrained by its reliance on external light excitation. In biological tissue, light is strongly scattered and absorbed, which limits penetration depth and, together with tissue autofluorescence, markedly lowers the signal-to-background ratio (SBR) for deep-seated targets 6. To bypass the limitations of external optical excitation, various “internal” signal-generation strategies have been explored, including chemiluminescence 7, 8, bioluminescence 9, Cerenkov luminescence 10, and X-ray-activated luminescence 11, 12, yet each entails additional limitations, such as the reliance on unstable chemical substrates or the potential risks associated with ionizing radiation (**Figure 1A**). Consequently, there is a need for an excitation strategy that offers deep tissue penetration, non-ionizing safety, and precise spatiotemporal control over luminescence *in vivo*.

Ultrasound (US) has emerged as an appealing excitation modality for deep-tissue luminescence because it can offer centimeter-scale penetration, non-ionizing safety profiles, broad clinical accessibility, real-time operability, and relatively low cost 13-17. The history of ultrasound-induced luminescence (**Figure 1B**) began with Frenzel and Schultes’ 1934 observation of sonoluminescence 18, in which collapsing cavitation bubbles emitted light^19^, ^20^. Jarman’s analysis clarified it as a consequence of violent bubble collapse 21. In the early 1990s, stable single-bubble sonoluminescence and synchronous picosecond flashes provided a reproducible model of acoustic-to-optical energy conversion 22, 23, though emission remained weak and short-lived for imaging. Subsequent work adapted this phenomenon for biomedical imaging and therapy. Early chemiluminescent probes such as fluoresceinyl cypridina luminescent analogue (FCLA) harnessed reactive oxygen species (ROS) generation during cavitation for *in vivo* signal detection 24, 25. A chemiluminescence-mediated sonodynamic diagnosis study converted ultrasound-induced reactive intermediates into tumor-localized optical readouts 26. Microfluidic sonochemistry and micromachined pit microreactors improved cavitation-driven luminescence control 27, 28. Mechanoluminescent materials shifted the field to solid-state light sources 29. In 2019, direct multi-bubble sonoluminescence under therapeutic ultrasound conditions with microbubbles linked sonoluminescence to biomedical ultrasound 30. Rechargeable mechanoluminescent nanotransducers enabled minimally invasive sono-optogenetics as deep-tissue light sources 31. Cascade-amplified mechanoluminescent nanotransducers subsequently increased light output for sono-optogenetic deep-brain stimulation 32. Integration with optical thermometry in 2022 produced multifunctional platforms 33. Organic nanoparticles later produced ultrasound-induced afterglow, or sonoafterglow, by converting ultrasound-generated singlet oxygen into delayed luminescence with reduced autofluorescence background and improved deep-tissue performance 34. Persistent-luminescence nanoplatforms were then applied to *H. pylori* theranostics and Janus-nanomotor-mediated tumor therapy 35, 36. Recent progress diversified emitter chemistry, imaging mode, and therapeutic integration. In 2024, ultrasound-excited purely organic mechanoluminescent materials achieved red and near-infrared region emission, expanding from inorganic or hybrid emitters to organic luminogens 37. In the same year, trianthracene-derived probes enabled real-time and delayed *in vivo* imaging with millimeter resolution and centimeter penetration 17, and a protocol enhanced reproducibility 38. In parallel, a 2025 study reported a hybrid nanosensitizer that couples cascade energy transformation with luminescence, enabling enhanced sonodynamic therapy and ROS-correlated luminescent output 39. Most recently, studies in 2026 have further accelerated the field such as programmable ultrasound-scanning light sources, ultra-bright fused-trianthracene sonoafterglow for fractionated therapy, and smart activatable nanobombs for hypoxic tumor imaging and gas-sonodynamic therapy 40-42. Together, these studies trace the transition from mechanistic investigations to engineered theranostic platforms.

Research on ultrasound-induced luminescence remains fragmented: mechanisms, material platforms, design principles, imaging strategies, biomedical applications, and translational challenges are rarely considered within a single framework. This review connects these components of ultrasound-induced luminescence. We focus on three paradigms, instant ultrasound-induced luminescence, sonoafterglow luminescence, and Off-On switching luminescence, and discuss the imaging mechanisms behind each. For each paradigm, we compare representative organic, inorganic, and hybrid systems in terms of their molecular design, structure–property relationships, advantages, and limitations. We then trace the energy pathway from energy pathway from excitation input through energy capture, transfer and transformation to radiative emission or nonradiative decay, providing a framework for discussing strategies to increase luminescent intensity, prolong emission lifetime, and tune emission wavelength. We also examine how molecular design and structure–activity relationships influence biocompatibility, targeting, stability, and metabolic clearance. Biomedical applications are discussed by disease model, with emphasis on cancer and disorders involving the immune and nervous system. Finally, we consider barriers to clinical translation and the requirements for developing practical theranostic platforms. **Table S1** summarizes this framework.

## Paradigms and mechanisms of ultrasound-induced luminescence

Based on whether emission persists after ultrasound cessation, ultrasound-induced luminescence can be classified as instant luminescence or sonoafterglow. Instant ultrasound-induced luminescence occurs primarily during ultrasound exposure. In sonoafterglow systems, part of the deposited acoustic energy is temporarily stored in metastable chemical intermediates or defect-associated trap states and released after ultrasound ceases. The two modes therefore serve different imaging purposes: instant luminescence supports real-time monitoring, whereas sonoafterglow provides sustained signals with reduced background for deep tissues imaging 17, 39. Off-On switching luminescence should be understood as an activatable gating strategy rather than a mutually exclusive emission-duration category. It can be integrated with either instant or sonoafterglow systems. In this hierarchy, the Off-On component determines where and when luminescence is activated, whereas the underlying instant or sonoafterglow mechanism determines how the signal is generated. We therefore discuss the Off-On mechanism separately to clarify its unique gating logic, which facilitates subsequent discussions on material design and potential applications. In Off-On switching luminescence, pathological biomarkers unlock luminescence during or after removal of ultrasound excitation, enabling prolonged and highly specific signal generation in targeted microenvironments 17. Each paradigm requires specific molecular design. Instant ultrasound-induced luminescence mainly depends on whether acoustic stimulation can be converted into emissive excited states rapidly and efficiently during insonation. Sonoafterglow luminescence differs from instant luminescence not only in lifetime, but also in its structural requirement that part of the ultrasound-derived energy must be stored and then released in a delayed manner after insonation stops. Off-on switching luminescence represents a distinct design dimension. Rather than defining how long emission persists, it controls whether the luminescent response is activated. These systems couple acoustic energy conversion to an activatable dark-to-bright gating module. **Table 1** compares the three operational paradigms in terms of their definitions, emission timescales, energy-conversion pathways, biomedical advantages, and principal limitations.

Beyond emission duration and signal gating, ultrasound-induced luminescence can also be classified according to three principal mechanisms: cavitation-dependent sonosensitization, piezocatalysis, and mechanoluminescence. In cavitation-mediated systems, ultrasound-induced cavitation generates ROS that inititate chemiluminescent reactions 17, 28, 39. In piezocatalytic platforms, ultrasound-induced stress separates charge carriers in piezoelectric materials. The resulting redox reactions generate ROS that subsequently drive luminescence 43. By contrast, the mechanoluminescent materials considered here produce light under mechanical stimulation through processes such as electron detrapping or lattice deformation rather than ROS-mediated reactions 37. Though conceptually different, these mechanisms may partly overlap in some hybrid systems under ultrasound. These mechanisms support both real-time insonation imaging and background-suppressed afterglow imaging once ultrasound stops 44, 45. To pin down the mechanism, quantitative analysis should establish correlations between acoustic inputs, ultrasound frequency, power density, and irradiation time and optical or chemical outputs, such as emission intensity, ROS generation, and decay behavior 17. Experimental validation using ¹O₂/ROS probes, electron paramagnetic resonance spin-trapping, piezoelectric-response measurements, persistent-luminescence characterization, and trap-state analysis can help tell apart ROS-mediated, piezoelectric, mechanoluminescent, and trap-controlled pathways 28, 33, 35, 46.

### Instant ultrasound-induced luminescence

Instant ultrasound-induced luminescence is defined as light emission occurring during, or immediately after ultrasound stimulation (**Figure 2A**). It arises mainly from acoustic cavitation, piezoelectric responses, thermal effects, and direct mechanical processes, where ultrasound energy is rapidly converted into localized chemical or electrical energy and then into optical emission 47. In practice, several ultrasound effects often act together, additively or synergistically 48. Acoustic cavitation can produce prompt photon emission through bubble dynamics, with efficiency set by the acoustic parameters and the dissolved-gas environment (**Figure 3A-B**) 49-52. Sonosensitizer-loaded nanoparticles or hybrid nanosensitizers can further amplify this process by generating ROS (*e.g.*, singlet oxygen (¹O₂) and hydroxyl radicals (^•^OH)), which oxidize chemiluminescent precursors and trigger emission through chemical excitation or resonance energy transfer 53. Thermal effects from ultrasound modulate thermoresponsive probes and emission intensity by way of local temperature changes 54-56. In piezoelectric materials, ultrasound-induced stress generates internal electric fields that drive charge separation and redox-mediated chemiluminescence (**Figure 3B, 3C**) 57-59. Vibration and pressure from ultrasound can directly activate mechanoluminescent materials 60-63. In trap-controlled inorganic mechanoluminescent phosphors, ultrasonic stimulation detraps electrons into the conduction band, followed by carrier migration to activator sites and radiative recombination (**Figure 3D**) 64. In organic systems, mechanical forces can also induce instant mechanoluminescence through deformation or fracture, such as crystal cracking or molecular disaggregation (**Figure 3E**) 65. These direct transduction routes constitute a major subclass of instant ultrasound-induced luminescence dominated by mechanical-to-optical conversion.

Instant ultrasound-induced luminescence produces optical signals primarily while ultrasound is applied. Depending on the material system, this response may involve cavitation, piezoelectric responses, thermal effects, or mechanically induced emission, each coupling acoustic excitation to light generation during stimulation.

### Sonoafterglow luminescence

In the broader context of luminescence, delayed emission can arise from several pathways (**Figure 4A**), including phosphorescence (10^-7^–10^-1^ s), ultralong phosphorescence (>0.1 s), thermally activated delayed fluorescence (TADF, microsecond–millisecond), and afterglow (>1 min) 66-68. The key distinction is not merely emission lifetime, but the origin of the delayed photons. TADF results from thermally assisted reverse intersystem crossing (RISC) from the triplet (T₁) to the singlet (S₁) state, producing short-lived delayed component emission from activated excitons (**Figure 4B**) 69. Sonoafterglow luminescence denotes delayed light emission that persists after ultrasound is removed, typically for minutes or longer. Strictly speaking, sonoafterglow relies on energy storage in long-lived trapped states or chemical intermediates that slowly release stored energy as photons 68. Mechanistically, delayed emission in sonoafterglow systems generally relies on two broad modes of energy-storage: chemical storage in metastable intermediates and physical storage in defect-associated electronic traps. In organic systems, ultrasound-induced cavitation or piezoelectric effects activate sonosensitizers (*e.g.*, Rose Bengal derivatives) to generate ROS (¹O₂ or O_2_^•-^). These species then oxidize substrates (*e.g.*, phenoxyl-adamantylidene derivatives) into high-energy emissive intermediates (**Figure 4C**), whose slow decay sustains sonoafterglow luminescence 70. In inorganic phosphors, ultrasound-induced localized heating, piezopotentials, or mechanochemical excitation can promote charge carriers into defect-associated traps within host lattices (*e.g.*, SrAl₂O₄, ZnS). Subsequent thermal release of these trapped carriers leads to recombination at luminescent centers such as Eu²⁺ or Mn²⁺/Cr³⁺ dopants, generating persistent afterglow emission (**Figure 4D**) 12. Some sonoafterglow systems may contain a RISC contribution, but when the long persistence is mainly determined by chemical reservoirs or carrier traps, the emission is better classified as afterglow luminescence rather than TADF alone.

Overall, the core characteristic of sonoafterglow luminescence is that energy storage is achieved through long-lived trap states or chemical intermediates after ultrasonic stimulation, and light signals are continuously released in a delayed manner after ultrasonic cessation.

### Off-On switching luminescence

Off-On switching luminescence is a design paradigm that decouples energy delivery from signal specificity, so that luminescence is produced only in the presence of defined biomarkers (**Figure 2C**). In this architecture, ultrasound supplies the required energy, whereas the probe is kept optically silent in its basal “Off” state through either chemical caging or proximity-based quenching (**Figure 5A-B**). In the caging design (**Figure 5A**), the active motif of a luminophore or sonosensitizer is covalently masked with a biomarker-responsive group (*e.g.*, a boronate ester responsive to peroxynitrite or an ether linkage responsive to hydrogen sulfide). In the quenching strategy (**Figure 5B**), a luminophore is placed near a quencher (such as black hole quencher-3 (BHQ-3) or the cyanine dye IR780) *via* a cleavable linker, suppressing emission through energy transfer. Activation, or the transition to the “On” state, occurs when a pathological biomarker removes the locking element, such as a cage or linker. This process may involve oxidative cleavage by ROS or reactive nitrogen species (RNS), such as peroxynitrite (ONOO⁻), proteolytic cleavage by matrix metalloproteinases (MMPs) or granzyme B, reductive activation by biothiols such as glutathione (GSH) or by hypoxia-associated reducing conditions, pH-triggered decomposition, and ion-driven displacement. Molecular recognition first restores the probe’s photophysical activity, after which ultrasound excitation generates a highly specific luminescent signal 34, 71.

Beyond activation chemistry, Off-On ultrasound-induced luminescence probes must also keep a low Off-state background and switch at an appropriate activation threshold, and remain reliable in biological environments. The Off state should remain sufficiently silent before biomarker activation. In a quencher-conjugated sonoafterglow nanoprobe for granzyme B imaging (Q-SNAP), proximity-based energy transfer to the quencher suppresses the sonoafterglow signal. After granzyme B cleaves the peptide linker and releases the quencher, the probe switches to the On state for T-cell activation imaging 72. In addition, Off-On switching is not strictly binary, but depends on local biomarker abundance, reaction efficiency, and ultrasound conditions. Therefore, parameters such as the activation threshold, On/Off ratio, and response kinetics are important for evaluating whether a detectable signal can be generated *in vivo*. In complex biological environments, partial activation or background leakage may arise from insufficient biomarker levels, incomplete biomarker-triggered reactions, nonspecific enzymatic activity, endogenous ROS/RNS, or off-target probe accumulation. This concern is reflected by ONOO⁻-responsive ultrasound-induced afterglow nanoparticles and the H₂S-activated dual-locked DPA-H₂S probe, which improve disease specificity through biomarker-gated activation but also highlight the need to evaluate leakage under physiologically relevant conditions 34, 71.

In Off-On switching luminescence, ultrasound provides excitation energy, whereas biomarker-responsive gating determines whether luminescence is generated. Caging or quenching suppresses emission in the Off state. Interaction with a pathological biomarker removes this suppression, allowing ultrasound to activate the luminescent response. By restricting signal generation to biomarker-positive environments, this design can reduce background emission and increase imaging contrast.

## Molecular design in available materials for ultrasound-induced luminescence

Materials used for ultrasound-induced luminescence include inorganic, organic, and hybrid systems. Although their conversion mechanisms differ, each couples acoustic excitation to optical emission through a specific pathway. Probe design must balance emission intensity and lifetime with biocompatibility, stability, and capacity for functionalization. Nanostructuring, the intergration of complementary conversion pathways, and spectral tuning toward the near-infrared (NIR) window have been used to support deep-tissue imaging and broaden functionality.

### Instant ultrasound-induced luminescence

Instant ultrasound-induced luminescence enables real-time visualization and on-demand activation. These systems can be broadly grouped into inorganic, organic, and hybrid platforms. Inorganic systems mainly exploit piezoelectric fields to drive direct carrier excitation, whereas organic systems rely on rapid cavitation-triggered chemical reactions or physical triboluminescence. Hybrid systems use interfacial synergy to amplify conversion efficiency and expand functionality.

### Inorganic systems

Inorganic systems for instant ultrasound-induced luminescence work mainly by converting sonomechanical energy directly into light. With generally shallow trap states, these materials emit mainly through direct piezoelectric excitation. Ultrasound-induced piezoelectric fields drive electron–hole recombination at luminescent centers, producing instantaneous emission. Structurally, these phosphors include a host lattice doped with activators (emitters) and, in some cases, sensitizers (absorbers) (**Figure 6A**) 73. Sensitizers harvest excitation energy and transfer it nonradiatively to activators, improving luminescence efficiency. Because of their accessible electronic states, rare-earth (RE) ions and transition metals are among the most widely used dopants 74, 75. Normally, REs exist as RE³⁺ ions in their respective compounds, in which Ce³⁺, Tb³⁺, Eu³⁺/Eu²⁺, Gd³⁺, Dy³⁺, Sm³⁺, Tm³⁺, Er³⁺, Yb³⁺, and Nd³⁺ are frequently employed because they can provide photostability, extended luminescence lifetimes, and narrow emission lines (**Figure 6B**) 76. The two primary categories of RE-doped phosphors are broad emission bands caused by f-d transitions and narrow spectral lines due to f-f transitions 77, 78. Meanwhile, Cr³⁺, Ni²⁺, Mn⁴⁺, Mn²⁺, and Fe³⁺ are most prevalent in transition metals 79, 80. Tungstates, vanadates, aluminates, borates, silicates, and phosphates are widely employed as phosphor hosts because of their thermal, optical, and structural stability (**Figure 6C**) 81. The effectiveness of these inorganic components in ultrasound-induced luminescence critically depends on the piezoelectric properties of the host and the specific dopants. A representative inorganic platform is SrAl₂O₄:Eu²⁺. Under ultrasonic stress, the non-centrosymmetric aluminate host generated a piezoelectric field, while Eu²⁺ acted as the activator responsible for green emission 82. Related co-doped systems, such as Sr₂MgSi₂O₇:Eu, Dy, followed a similar design. In these materials, the silicate host provided the piezoelectric response, whereas Dy³⁺ modified the trap states and thereby influenced the efficiency of ultrasound-induced luminescence from Eu²⁺ 82.

Together, these examples identify dopant identity, lattice piezoelectricity, and trap depth as key variables in converting acoustic stress into radiative recombination. Their effects on response rate and signal stability during repeated insonation depend on the carrier-trapping and release dynamics of the specific material.

### Organic systems

Organic systems produce instant ultrasound-induced luminescence through molecule luminophores or composite organic formulations. Typical representatives of such systems include nanoparticles prepared from trianthracene derivatives (TD), semiconducting polymers (*e.g.*, PFODBT, PFBT, MEHPPV), porphyrins (*e.g.*, Ce6, F-PpIX), boron-dipyrromethene dyes (BODIPY, BODIPY-Br), cyanines (ICG, IR780, HD), A-D-A'-D-A conjugated molecules (*e.g.*, BTz-IC-H, BTz-IC-F, BTz-IC-Cl), and curcumins (**Figure 7A**) 17. In many liquid-phase systems, ultrasonic cavitation generates ROS through sonochemical processes. In piezoelectric or piezocatalytic formulations, ultrasound-induced mechanical stress can also promote charge separation and ROS generation. These ROS then rapidly oxidize the luminophores and trigger photon emission. For example, TD nanoparticles contained an oxidizable motif that reacted with piezocatalytically generated ROS (¹O₂ and ^•^OH), preferentially forming TD–^•^OH and dioxetane intermediates. Subsequent cleavage proceeded mainly through C–C bond rupture, releasing chemical energy that was transferred to neighboring TD molecules to generate luminescence. Under standard ultrasound conditions (30 kHz, 4.5 W cm⁻²), this two-step intraparticle energy-conversion process produced a luminescence signal more than 1428 times stronger than water sonoluminescence. The resulting signal supported deep-tissue imaging with high spatial resolution 17. Organic crystalline materials constitute another class and exhibit mechanoluminescence in response to processes such as friction, fracture, or dislocation. The emission is generally attributed to charge separation during crystal fracture, or piezoelectric effects within the lattice. Typical examples include 9-anthracenemethanol 83, p-anisidine 84, resorcinol, methyl salicylate derivatives 85, borate derivatives 86-88, tetraphenylethene derivatives 89, N-phenyl imide derivatives 90, triphenylphosphine derivatives 91, dioxetanes 92, coumarin derivatives 93, anthracene derivatives 94, which can emit mechanoluminescence from blue to yellow in their crystalline state (**Figure 7B**). Unlike ROS-mediated chemical pathways in liquid-phase probes, these crystals rely on physical structural changes. Phenothiazine derivatives, for instance, reversibly switch between “butterfly-like” quasi-axial and quasi-equatorial conformations under mechanical stimulation, accompanied by corresponding changes in emission 95. Organic composites have also been incorporated into supramolecular assemblies and lipid formulations to improve stability and light output. Lipo@IR780/L012 co-encapsulated the chemiluminescent probe L012 and the sonosensitizer IR780 within a liposomal nanoplatform, producing blue light in response to focused ultrasound excitation 96. The molecular design of L012 featured a hydrazide bond (N–N bond) or pyridazine heterocyclic core that was highly susceptible to oxidation by ROS (¹O₂ and ^•^OH) generated from IR780 under focused ultrasound (FUS) stimulation. This specific structure underwent rapid ROS-triggered bond cleavage, leading to the formation of an excited-state intermediate that subsequently emitted blue light. With this structure–activity relationship, ROS were rapidly consumed, limiting residual cytotoxicity while providing efficient chemiluminescence for deep-brain optogenetic applications. In another formulation strategy, confinement of carbon nanodots (CNDs) within cyclodextrin-based supramolecular assemblies suppressed nonradiative vibrational relaxation and increased quantum efficiency 97. Lipid-coated oxygen microbubbles, including modified Lumason®, enhanced cavitation-induced excitation near sonosensitizers, an effect attributed to the lower ionization energy of oxygen 98.

Together, these examples illustrate several routes to immediate ultrasound-induced emission: mechanically responsive molecular packing, ROS-activated luminophores, or supramolecular suppression of nonradiative decay, and formulations that enhance cavitation.

### Hybrid systems

In hybrid systems for instant ultrasound-induced luminescence, organic and inorganic components are integrated through interfacial coupling and hierarchical assembly to couple acoustic excitation to rapid light generation 99, 100. The inorganic phase can provide a piezoelectric response or cavitation-nucleation sites, whereas the organic phase supplies the luminophore and offers molecular tunability 32, 101. Interfacial processes, including piezoelectrically driven charge separation and catalytic ROS generation, couple these functions and can enhance emission during ultrasound exposure. For instance, combining gold nanoparticles (GNPs) with protoporphyrin IX (PpIX) yielded Au–PpIX, an ultrasound-responsive hybrid nanoconjugate (**Figure 8A**) 53. In the molecular design of Au–PpIX, a sulfur-containing PpIX derivative was conjugated to GNPs *via* Au–S bonds, owing to the presence of a sulfur atom in the organic moiety (C_40_H_45_N_4_O_4_S). PpIX acted as a sonosensitizer, generating reactive species under ultrasound, while the GNPs served as cavitation nuclei to promote cavitation. The design gave a dual synergy: the GNPs raised the cavitation rate and provided nucleation sites, while PpIX released more free radicals once activated. The result was stronger sonoluminescence and more efficient sensitization in ultrasound-based applications. Another nano-hybrid system assembled the chemiluminescent probe L012 (luminol analogue with a hydrazide N–N bond), the sonosensitizer IR780 (conjugated polyene–heterocycle), and polyethylene glycol-coated calcium peroxide (CaO₂, containing O–O peroxide bonds) within liposomes, all of which responded to ultrasound together (**Figure 8B**) 32. Under ultrasound, IR780 generated ROS (¹O₂ and ^•^OH) *via* cavitation; mechanical perturbation disrupted the PEG coating, letting CaO₂ react with water to yield H₂O₂ and Ca(OH)₂, which raised local pH and free radical concentration. The ROS and H₂O₂ oxidized the N–N bond of L012, driving excited-state intermediates to emit blue light, while the higher pH prevented protonation of L012 and so improved its quantum yield. This structure–activity relationship gave a cascaded amplification of blue light emission, enabling low-latency optogenetic activation at depths up to 5 mm for minimally invasive deep-brain sono-optogenetics. Mesoporous silica nanoparticles (MSNs) served as carriers for inorganic phosphor hybrids, including Zn_2_Ga_2.96_Ge_0.75_O_8_:Cr_0.02_,Nd_0.02_ nanoparticles assembled within MSNs to form Zn_2_Ga_2.96_Ge_0.75_O_8_:Cr_0.02_,Nd_0.02_ (ZGGO) @MSN composites 64, and Zn_1.2_Ga_1.6_Sn_0.2_O_4_:0.5%Cr^3+^ (ZGSO) material embedded in MSNs 101. Both hybrids relied on the same ultrasound-induced mechanoluminescence mechanism, a piezoelectric-field-induced trap-detrapping process followed by Cr³⁺ ²E→⁴A₂ (and ⁴T₂→⁴A₂) emission to generate NIR luminescence. Yet their trap chemistries and energy-transfer pathways diverged in critical ways. Ge⁴⁺ in ZGGO created a broad distribution of shallow and deep traps (0.7–1.2 eV) and enabled Cr³⁺→Nd³⁺ energy transfer, producing NIR-II afterglow (~1062/1335 nm) for deep-tissue lymph node immunotherapy. By contrast, Sn⁴⁺ substitution in ZGSO introduced shallower traps and lacked secondary energy transfer, confining its emission solely to the Cr³⁺ ²E→⁴A₂ NIR-I peak (~694 nm) for macrophage engineering (**Figure 8C**). Another molecular design encapsulated iron doped zinc oxide nanocrystals in a self-assembled lipid shell, giving a core-shell ZnO-Lip hybrid material (**Figure 8D**) 102. The semiconductor ZnO core (with Zn²⁺/Fe³⁺ ions introducing defect states) and the dynamic lipid shell worked together. Under ultrasound, the lipid shell nucleated and stabilized gas bubbles, sustaining inertial cavitation and generating prolonged sonoluminescence without direct bubble attachment to the nanocrystal surface. Meanwhile, the ZnO nanocrystals enhanced sonoluminescence by serving as cavitation nucleation sites and possibly through charge-transfer processes. The result was a single biocompatible nanoplatform capable of both sonoluminescence-based cell imaging and contrast-enhanced ultrasound imaging.

Instant ultrasound-induced luminescence can therefore be realized through three distinct but related molecular logics, namely direct piezoelectric recombination in inorganic phosphors, ROS-mediated or mechanoluminescent activation in organic systems, and interfacial amplification in hybrid architectures.

### Sonoafterglow luminescence

Sonoafterglow luminescence persists after ultrasonic excitation is removed, enabling high-contrast bioimaging by temporally separating excitation from readout and markedly reducing tissue autofluorescence and prompt scattering. Materials for this modality fall into three classes, each suited to particular biomedical needs. Inorganic systems rely on defect-mediated carrier trapping, and are valued for robust physicochemical stability, resistance to photobleaching, and tunable ultra-long emission. Organic systems work through cascade generation of metastable chemical intermediates, offer intrinsic biocompatibility, structural versatility, and relatively facile metabolic clearance, which makes them well suited for *in vivo* use. Hybrid systems integrate organic and inorganic components through interfacial processes such as catalytic amplification and resonance-energy-transfer-based recharging 103. By coupling complementary functions, these processes can improve energy conversion and support combined imaging and therapeutic functions. Any advantage over the corresponding single-component systems, however, should be established through direct comparison under matched conditions.

### Inorganic systems

Inorganic sonoafterglow materials store excitation energy in deep trap states and release it as prolonged light emission after ultrasound is switched off. This behavior usually requires defect engineering or composite architectures that promote deep, efficient trap filling under ultrasonic stimulation. As a representative, CaZnOS:Nd³⁺ used a piezoelectric semiconductor matrix. Under ultrasound, the generated piezoelectric field promotes detrapping of charge carriers from deep traps (enhanced by Nd³⁺ doping) and refills them. After ultrasound ceases, these trapped carriers are gradually released through thermal activation (heterologous detrapping) and recombine at Nd³⁺ centers *via* ⁴F₃/₂→⁴I₁₁/₂ and ⁴F₃/₂→⁴I₉/₂ transitions, producing a persistent NIR afterglow tunable within the biological window 104. Another platform used Cr³⁺-doped ZnGa₂O₄ glass-ceramic composite, where the glass matrix introduced a high density of interface-associated traps that retained trapped carriers. After excitation, carriers were thermally detrapped and recombined at Cr³⁺ centers *via* the ²E→⁴A₂ transition, yielding persistent red-to-NIR luminescence compatible with the biological imaging window 105. Through such deep-trap or interface-trap engineering, these inorganic systems convert mechanical energy into a stable optical signal for acoustic read-out and stress sensing, and their deep-trap principle offers a blueprint for engineering biocompatible sonoafterglow nanomaterials toward biomedical applications.

### Organic systems

Organic afterglow systems rely more on excited state stabilization and suppression of fast nonradiative decay. Here, molecular rigidification, aggregation control, and enhanced intersystem crossing (ISC) matter most. Organic sonoafterglow systems usually couple a sonosensitizer to a chemiluminescent substrate in a cascade, so luminescence persists once ultrasound stops. Their molecular design follows two strategies. The first is co-loading: both components are co-loaded in one nanoparticle, so collisional energy transfer proceeds efficiently through the Chemically Initiated Electron Exchange Luminescence (CIEEL) cascade. The second is conjugation: a molecular linker covalently ties the sonosensitizer to the afterglow substrate, promoting intramolecular energy transfer and tighter spatiotemporal control. Common sonosensitizers include rose bengal octyl ester (RB), hematoporphyrin (HMP), verteporfin (VP), silicon 2,3-naphthalocyanine bis(trihexylsilyloxide) (NCBS), and chlorin e6 (Ce6). They are paired with chemiluminescent substrates such as phenoxyl-adamantylidene (PA), azide-methyl acrylate-phenoxyl-adamantylidene (AMPA), poly(2-methoxy-5-(2′-ethylhexyloxy)-1,4-phenylene vinylene) (MEHPPV), dicyanomethylene-4H-benzopyran-phenoxyl-adamantylidene (DPAo), and dicyanomethylene-4H-benzothiopyran-phenoxyl-adamantylidene (DPAs) (**Table 2**) 17, 32, 34, 38.

The excitation behavior of a sonosensitizer largely depends on its triplet quantum yield and triplet lifetime. Porphyrin-based sonosensitizers such as ZnPc, PpIX, and Ce6 contain an 18π-electron aromatic macrocycle that promotes efficient intersystem crossing and triplet-state formation, raising the ¹O₂ quantum yield under ultrasound 106. Under ultrasound (*e.g.*, 1 MHz, 1.5-2.0 W cm⁻², 30-60 s), the sonosensitizer efficiently generated singlet oxygen (¹O₂) through the type II mechanism. The generated ¹O₂ then reacts with the phenoxyl adamantylidene moiety of the substrate *via* [2+2] cycloaddition, forming a high-energy 1,2-dioxetane intermediate. The four membered cyclic peroxide structure was strained and unstable. Through the CIEEL mechanism, which involved intramolecular electron transfer and back electron transfer, the dioxetane slowly decomposed with a half-life of 70-180 s. The decomposition produced a chemically excited benzoate species that emitted afterglow luminescence, and the wavelength was determined by the molecular orbital energy gap of the chemiluminescent fragment. Importantly, the NCBS sensitizer can be re-excited directly by the chemical energy released from the dioxetane, a self-charging feature that prolonged the afterglow. This structure-activity relationship, namely dioxetane ring strain leading to CIEEL thermal decomposition leading to a chemically excited state and direct sensitizer re-excitation, was the molecular foundation of sonoafterglow. Consequently, NCBS/DPAs nanoparticles can deliver afterglow intensities 2.4-fold higher than photo-induced counterparts and maintain a signal-to-background ratio of 12.5 even at 4 cm depth of tissue 34.

The ultrasound-activated NIR-II afterglow probe NPs-Ce4-SN adopted a more advanced design. Here the NIR-II emissive unit was covalently integrated into the afterglow substrate skeleton instead of relying on intermolecular Förster resonance energy transfer (FRET) from a NIR-I intermediate. Under ultrasound, Ce6 generated ¹O₂ and oxidized the substrate to form a dioxetane whose C=O π conjugation extended to a benzobisthiadiazole (BBTD) NIR-II chromophore. Upon dioxetane decomposition *via* CIEEL, excitation energy was transferred intramolecularly to the BBTD acceptor through a through-bond energy transfer (TBET) mechanism, a single-step process that avoided the efficiency losses of collisional transfer. Molecular orbital tuning red-shifted the emission peak by nearly 400 nm versus conventional afterglow probes, reaching ~1100 nm in the NIR-II window. Electron-withdrawing cyano groups on the BBTD unit lowered the LUMO, while donor–acceptor alternation along the conjugated backbone modulated the HOMO. Emission within this tissue -transparent window supported high signal-to-noise imaging at centimeter depths 107.

FZ970 added a chemiluminescence resonance energy transfer (CRET) dual-lock design. The afterglow substrate FZA was made by covalently tethering a chemiluminescent adamantylidene unit to a NIR-II BBTD emissive core, then co-encapsulated with ZnPc sonosensitizer in a DSPE-PEG2000 nanoparticle. Under ultrasound, ZnPc generated ¹O₂, converting the adamantylidene unit into a dioxetane. CIEEL decomposition then chemically excited the adjacent BBTD core through an intramolecular CRET pathway. Critically, an ONOO⁻ responsive aryl boronate group, specifically a phenylboronic acid pinacol ester, was placed in the molecular linker between the adamantylidene and BBTD units. This group stayed intact in the absence of ONOO⁻ but was specifically hydrolyzed in the presence of tumor-overexpressed ONOO⁻, removing a quenching moiety that otherwise suppresses CRET efficiency. Therefore, both ultrasound (to generate ¹O₂ and form the dioxetane) and ONOO⁻ (to cleave the quencher and restore CRET) were required for afterglow activation, creating an AND logic molecular gate. This dual-control design, which combined sonosensitizer ¹O₂ generation, ONOO⁻ responsive dequenching, and intramolecular CRET, reduced non-specific background signals in healthy tissues. The afterglow intensity reached 2.4 times that of traditional systems, and the intramolecular CRET design improved energy transfer efficiency from about 40% in intermolecular systems to about 70% in the conjugated architecture, achieving millimeter-resolution NIR-II afterglow imaging with real-time therapeutic monitoring 106.

In summary, all these organic sonoafterglow systems rely on a common molecular logic, including dioxetane formation, CIEEL-mediated slow decomposition, and energy transfer to a luminescent reporter. The performance is continuously improved by covalently integrating the emitter, engineering orbital energy levels, and introducing responsive molecular gates. These purely organic designs complement the inorganic and hybrid systems discussed elsewhere in this section on molecular design.

### Hybrid systems

For sonoafterglow luminescence, hybrid systems are engineered to address the limited energy-storage capacity of single-component materials by introducing *in situ* recharging pathways. In contrast to instant systems that prioritize immediate sonomechanical-to-optical conversion, these architectures leverage interfacial coupling to sustain emission after the acoustic field is removed. Hybrid sonoafterglow systems are commonly implemented in two ways: (1) coupling a mechanoluminescent “harvester” that converts ultrasound into light with a persistent “emitter” that stores energy and releases it as afterglow; (2) incorporating an inorganic catalytic component that continuously generates reactive intermediates to prolong the emission of organic probes after removal of ultrasound excitation.

A representative example of the former was the NPs@CoOOH hybrid system (**Figure 9A**), in which semiconducting polymer nanoparticles (SPNs) were coupled with cobalt oxyhydroxide (CoOOH) through surface coordination 39. In this system, organic sonosensitizing nanoparticles were first prepared by nanoprecipitation using DSPE-PEG and PSMA. The PSMA was subsequently hydrolyzed to generate surface carboxyl groups for cobalt ion binding. Coordination of Co²⁺ followed by oxidation in alkaline NaClO solution formed a CoOOH shell on the nanoparticle surface. Under ultrasound, CoOOH contributed to ¹O₂ amplification, while ultrasound also promoted electron-hole separation in the SPNs to generate **^•^**OH. These ROS then reacted with thiophene units in the polymer, releasing chemical energy that was transferred to intact polymer chains and ultimately converted into afterglow luminescence. This hybridization approach also exhibited good generality and was extended to various organic components with active sites (*e.g*., other semiconducting polymers, cyanines, porphyrins), as long as they can interface with CoOOH.

The molecular design of hybrid nanomaterial mSZ-Clr@ePDA NPs for ultrasound-triggered luminescence relied on a dual-nanodot core deposited on mesoporous silica nanoparticles (MSNs): mechanoluminescent SrAl₂O₄:Eu²⁺ (SAOE) nanodots and persistent-luminescent ZnGa₂O₄:Cr³⁺ (ZGC) nanodots 36. Under ultrasound excitation, the piezoelectric field generated in the non-centrosymmetric SrAl₂O₄ lattice facilitated electron detrapping from oxygen vacancy or Dy³⁺-induced shallow traps, followed by recombination at Eu²⁺ centers, producing green mechanoluminescence. This green emission matched the excitation of the ZGC shell and transferred energy *via* FRET, where Cr³⁺ substituted Ga³⁺ at octahedral sites, forming Cr³⁺-defect clusters. The host-lattice O²⁻ → Ga³⁺ charge-transfer transition stores electron–hole pairs in these Cr³⁺-associated defect levels; thermally released electrons recombine at Cr³⁺ centers *via* the ²E → ⁴A₂ transition, yielding persistent NIR afterglow. Initially, the organic enzyme-responsive polydopamine (ePDA) coating quenched the emission. Exposure to *H. pylori*-secreted phospholipase C hydrolyzed the coating and thereby restored the afterglow. The same research team further reported the Janus mSZ@PDA-NO nanoparticle hybrid system, which shared the identical SAOE/ZGC core as mSZ-Clr@ePDA, and thus relied on the same ultrasound-rechargeable persistent NIR afterglow *via* FRET (detailed above) 35. The distinction lay in its asymmetric organic functionalization with a polydopamine (PDA) layer loaded with nitric oxide (NO) donors (**Figure 9B**). Unlike the uniform ePDA shell that quenched luminescence for enzyme-gated imaging, this Janus structure converted the same persistent NIR afterglow into self-thermophoretic force and NO-gas propulsion to drive autonomous motion. The design may be extended to other mechanoluminescent (*e.g.*, CaAl_2_O_4_:Eu^2+^, Nd^3+^) or afterglow (*e.g.*, CaTiO_3_:Pr^3+^, Y_2_O_2_S:Eu^3+^) pairs, provided that spectral overlap supports efficient energy transfer, and can be further hybridized with organic polymers or bio-functional layers to achieve tailored theranostic functions 108.

In this way, inorganic materials contribute trap-governed persistence, organic materials contribute chemically programmable afterglow, and hybrid materials solve the limited storage or recharging capacity of single-component systems.

### Off-On switching luminescence

In addition to instant and afterglow luminescence, switchable Off-On ultrasound-induced luminescence should be discussed as a third materials-oriented mode because its defining feature is gated emission rather than prompt or persistent output. Materials (or probes) engineered for Off-On switching luminescence achieve high specificity through molecular caging or quenching architectures that respond to defined environmental triggers (**Table 3**) 109, 110. In caging designs, active luminophores are silenced by covalent masking groups that undergo trigger-specific cleavage. For instance, the probe SNAP-M used an ONOO⁻-responsive boronate ester to cage the DPAs substrate; ONOO⁻-mediated cleavage restored the reactivity of the substrate toward ultrasound-generated ¹O₂, enabling afterglow emission. Similarly, a sonoafterglow cancer nanoimmunotheranostic probe (SCAN) caged methylene blue (MB) with the same boronate ester and the immunomodulator imiquimod (R837) *via* a singlet-oxygen-labile linker, enabling sequential unlocking driven by ONOO⁻ and ¹O₂ 17, 34. Another example, DPA-H₂S, employed a 2,4-dinitrobenzene (DNB) ether to lock a luminogenic precursor. Reductive cleavage by hydrogen sulfide (H₂S) unmasked a reactive double bond 71. Caging motifs widely used in non-ultrasound probes, such as pH-labile linkages (*e.g.*, acetals, hydrazones) or hypoxia-activated nitroreductase (NTR)-cleavable groups, can also be adapted for ultrasound-activated probes 109, 110.

Quenching-based systems rely on proximity-dependent energy transfer. One example is trianthracene-derivative (TD) nanoparticles conjugated to the quencher BHQ-3 through enzyme-cleavable peptide linkers Ile-Glu-Phe-Asp peptide (IEFD) for granzyme B, Gly-Pro-Leu-Gly-Ile-Ala peptide (GPLGIA) for matrix metalloproteinase-2 (MMP-2), and Asp-Glu-Val-Asp peptide (DEVD) for caspase-3 protein. Proteolytic cleavage physically separated BHQ-3 from the TD emitter, thereby relieving quenching and restoring luminescence 17, 34. A “self-sacrificing” quencher strategy was demonstrated by TD@IR780, in which the IR780 dye was oxidatively degraded by ONOO⁻, thereby abolishing quenching. Beyond molecular cleavage, hybrid systems can physically gate emission using pH-sensitive dyes (*e.g.*, bromothymol blue), while biohybrid sonogenetic materials used mechanosensitive channel of large conductance (MscL) to regulate Ca²⁺ influx and activate bioluminescent proteins such as aequorin 71, 111.

Different classes of ultrasound-induced luminescent platforms exhibit clear trade-offs among structural complexity, functional integration, and translational feasibility (**Table 4**). Molecularly defined small-molecule probes are clear in composition, easy to characterize, and reproducible between batches, but their* in vivo* stability and acoustic amplification may still be limited. Self-assembled organic nanoplatforms can amplify signals and integrate theranostic functions, but their formulation-sensitive performance complicates quality control. Polymeric and semiconducting platforms support modular design and ROS amplification, but their compositional heterogeneity complicates scale-up. Liposomal systems may offer a more established development route because manufacturing methods and regulatory experience already exist for some liposomal products. However, this precedent does not by itself establish the translational feasibility of a specific ultrasound-responsive formulation. Inorganic persistent-luminescent platforms can be recharged by ultrasound and sustain emission after excitation, but limited biodegradation and long-term tissue retention remain concerns. Hybrid multifunctional systems can combine closed-loop imaging, immune activation, and combination therapy, although their compositional complexity creates challenges for quality control, standardization and manufacturing.

This section maps the materials available for ultrasound-induced luminescence. Instant emission favors rapid output, afterglow favors persistent output, and Off-On systems favor controllable output. Within each mode, inorganic, organic, and hybrid materials reach luminescence by distinct yet related molecular design routes.

## Luminescent performance enhancement strategies for energy pathways

How molecular structures and nanoassemblies regulate acoustic energy conversion pathways ultimately governs the imaging and therapeutic performance of ultrasound-induced luminescent systems, setting signal brightness, persistence, penetration depth, and therapeutic output. This section examines how targeted molecular design, including donor–acceptor modulation, conjugation extension, and conformational rigidification, shapes each step of the energy-conversion pathway: (Stage 1) excitation input, (Stage 2) energy capture, (Stage 3) energy transfer and transformation, and (Stage 4) competition between radiative and nonradiative processes (**Figure 10**). Tuning the bandgap, charge-transfer character, intersystem crossing probability, and nonradiative decay pathways is key to building the structure–activity relationships that govern ultrasound-induced luminescence. These relationships decide whether acoustic excitation gives red-shifted, persistent, and biologically detectable emission, and thus the material's imaging performance and therapeutic potential. These four energy stages are kinetically intertwined and cannot be strictly isolated in most molecular systems. However, we present them as distinct steps and highlight the dominant stage for each design strategy in the following examples for clarity of discussion.

### Enhancing energy excitation *via* cavitation amplification

The first step in optimizing ultrasound-induced luminescence is to increase the efficiency with which acoustic energy is introduced into the system. At this stage, the most effective molecular and nanoassembly strategies include constructing cavitation-active composite architectures, introducing interfaces that concentrate local stress, and designing nanostructures that repeatedly nucleate or stabilize bubbles under insonation (**Figure 10A**). The governing structure–property relationship is that component arrangement, interfacial accessibility, and shell dynamics determine how effectively ultrasound can be converted into localized cavitation events, which then serve as the initial source of excitation. Improvements at this stage are therefore reflected mainly in stronger signal onset, lower activation threshold, higher excitation sensitivity, and in some cases greater usable imaging depth.

This can be achieved by introducing engineered nanostructures that serve as artificial cavitation nuclei, lowering the energy threshold for bubble formation and increasing the density and efficiency of bubble activity. A representative example was an Au-PpIX nanoconjugate, in which GNPs acted as preferential cavitation nucleation sites that locally concentrated acoustic energy to increase cavitation rate and intensity. The sensitizer PpIX, in turn, benefited from the enhanced cavitation field. The GNPs may also reshape the local microenvironment of PpIX, prolonging its nonradiative relaxation time and improving energy conversion efficiency 53. Another strategy is to stabilize cavitation bubbles, as core-shell ZnO-lipid (ZnO-Lip) structures showed. Bare ZnO nanocrystals trapped transient microbubbles, and triggered immediate but short-lived inertial cavitation. By contrast, the dynamic lipid shell of ZnO-Lip prevented irreversible bubble entrapment and remained dynamically reconfigurable under ultrasound, enabling sustained bubble nucleation and stable oscillation for minutes. Bare ZnO gave a strong immediate signal that quickly decayed, whereas ZnO-Lip showed a time-dependent onset of persistent inertial cavitation; this gave enhanced, prolonged sonoluminescence and supported applications such as high-definition *in vitro* cell visualization 102.

### Optimization of energy capture *via* trap engineering

At the energy capture stage, molecular design sets how efficiently the system accepts and holds the incoming excitation. The main strategies are bandgap regulation, donor-acceptor modulation, heteroatom or dopant incorporation, and local-environment control. Tuning molecular parameters such as bandgap and charge-transfer character can increase the population of emissive excited states instead of being lost prematurely. In inorganic systems, trap-states engineering regulates carrier capture, storage, and release under ultrasound stimulation (**Figure 10B**). Three approaches are shown for modifying trap characteristics and coupling trapped carriers to subsequent energy-transfer pathways.

First, piezoelectric hosts can drive detrapping with ultrasound-induced internal fields. The CaZnOS crystal family illustrates this: doping with various ions such as Mn²⁺, Cu⁺, Bi³⁺, Er³⁺, Nd³⁺ introduces luminescent centers, while the host's intrinsic piezoelectricity supplies the ultrasound-induced fields that release captured carriers. CaZnOS:Nd³⁺ showed strong mechanoluminescence in the biologically transparent NIR region, highlighting the host-dopant synergy in tuning both emission and trap-release mechanisms 104. A recent ZnS-based study further demonstrated the value of dopant engineering. ZnS nanocrystals doped with Ag⁺, Cu²⁺, or Mn²⁺ gave focused ultrasound-induced mechanoluminescence at 480, 500, and 585 nm, respectively, showing that controllable dopant incorporation can tune emission color in nanoscale mechanoluminescent systems 112.

Second, bandgap and defect engineering can be used to create a high density of stable deep traps to prolong energy storage. For example, in Zn_1.2_Ga_1.6_Sn_0.2_O_4_:0.5%Cr^3+^ embedded in mesoporous silica (ZGSO@MSN), optically generated carriers were captured by deep traps. Ultrasound-induced mechanical stress then generated a local piezoelectric field that promoted carrier detrapping back to the conduction band, ultimately leading to NIR-I emission centered at 694 nm *via* the ²E to ⁴A₂ transition of Cr³⁺. The emission spectrum of this system (from Cr³⁺) overlapped with its absorption spectrum (mainly corresponding to transitions like ⁴A₂ to ⁴T₂ of Cr³⁺) in specific bands (*e.g.*, the 580 nm excitation band), which was primarily involved in its optical excitation process. In the ultrasound-triggered mechanism, the stability of deep traps is critical for maintaining a stable mechanoluminescence output even after shallow traps are exhausted 101.

Third, trap engineering can be integrated with resonant energy transfer to achieve wavelength conversion through spectral matching. At the level of inorganic persistent-luminescence materials, heteroatom substitution and dopant engineering primarily regulate trap depth, defect distribution, radiative transitions, and detrapping kinetics, which in turn determine whether ultrasound stimulation can be converted into stable and tissue-penetrant optical signals. This is exemplified in the Cr³⁺/Nd³⁺ co-doped Zn₁.₂Ga₁.₆Sn₀.₂O₄ nanoclusters embedded in mesoporous silica, forming the ZGGO@MSN system (*i.e.*, the focused ultrasound-responsive immunomodulator-loaded nanomaterial (FURIN) nanocomposite). In this system, the ultrasound-triggered emission band from Cr³⁺ ions (710-850 nm, originating from the ⁴T₂ to ⁴A₂ transition) effectively overlapped with the characteristic absorption peaks of neighboring Nd³⁺ ions (at 745 nm and 805 nm, corresponding to the ⁴I9/2 to ⁴F7/2+⁴S3/2 and ⁴I9/2 to ⁴F5/2+²H9/2 transitions). This spectral overlap enabled efficient resonant energy transfer (from Cr³⁺ to Nd³⁺), thereby converting the captured mechanical energy into final NIR-II emission from Nd³⁺ at wavelengths such as 1062 nm and 1335 nm. This cascade linked carrier trapping and detrapping with cross-ion energy transfer, to increase energy absorption and shift the emission toward a biologically relevant spectral window 64. A related SrAl₂O₄-based system further showed that Nd³⁺ co-doping can markedly enhance persistent mechanoluminescence in SrAl₂O₄:Dy³⁺-Eu²⁺ phosphors, while Eu²⁺→Nd³⁺ energy transfer introduced long-lasting NIR emission for sound-to-light conversion and luminescence thermometry 113.

### Promotion of energy transfer and transformation *via* multi-level management

In Stage 3, captured excitation is redistributed among downstream channels. The key molecular strategies are: amplifying the ROS-mediated chemical-energy pool, opening direct intramolecular relay pathways that limit energy leakage, building intermolecular transfer circuits within nanostructured assemblies, and strengthening intersystem crossing or chemiluminescence resonance energy transfer. The goal is to funnel chemically stored energy efficiently into a chosen emitter. The structure-activity relationship at this stage lies in how molecular packing, donor-acceptor geometry, spin-related coupling, and interfacial pathway design determine whether the captured energy is converted into radiative singlet emission, long-lived triplet-related states, reactive intermediates, or gated signal outputs. Therefore, this stage has the broadest impact on luminescence intensity, emission wavelength, persistence time, and activatable functionality. Optimizing energy conversion focuses on maximizing the photon emission yield by designing efficient energy transfer and conversion channels, which can be categorized into three complementary strategies based on the dominant transfer mechanisms (**Figure 10C**).

First, the initial chemical-energy pool can be amplified by increasing ROS generation through multi-mechanistic designs. For instance, the hybrid SPNs@CoOOH nanosensitizer achieved a 3.5-fold increase in ROS *via* a dual-pathway mechanism: ultrasound-enhanced chemodynamic catalysis that converted water to singlet oxygen by CoOOH and improved electron-hole separation within SPNs for hydroxyl radical formation 39. Similarly, CaO₂-based liposomal transducers co-loaded with IR780 and L012 augmented ROS and locally raised pH, which deprotonated and activated the L012 luminol to significantly boost its chemiluminescence quantum yield 32. FTA NPs also achieved this sustained energy excitation by generating prolonged ROS for improved afterglow luminescence. Upon ultrasound irradiation, the piezoelectric effect of FTA directly converted mechanical energy into electrical energy, generating electrons (e⁻) and radical cations (FTA^•^⁺) that initiated immediate ROS production (O₂^•^⁻, ^•^OH, ¹O₂). Generated ROS oxidized FTA to form unstable endoperoxide intermediates (EPO I and EPO II), which acted as temporary chemical energy reservoirs. After ultrasound ceased, these endoperoxides decomposed slowly, releasing the stored energy to re-excite FTA molecules, thereby driving prolonged ISC and subsequent energy transfer to ¹O₂. This “capture-store-release” cycle sustained both ROS production and afterglow luminescence for over 15 min after irradiation. To increase cumulative energy output, a fractionated ultrasound regimen (multiple low-power pulses with dark intervals) was applied. By repeatedly charging the energy reservoirs and permitting delayed release, it raised total ROS yield by 2.5-fold over continuous irradiation at the same total dose 41.

Together, donor–acceptor (D–A) modulation and conformational rigidification can tune energy conversion pathways to support high-contrast imaging and therapeutic efficacy in the reported models. In PpIX-M, the TPP⁺ modification played two roles: a bulky 3D substituent that suppressed π–π stacking of the porphyrin plane, and a non-conjugated electron acceptor that introduced D–A character. Of the four energy stages, Stage 3 (energy transfer and transformation) was the most important for PpIX-M. The D–A modulation drove intramolecular charge separation and stabilized a long-lived charge-separated state, which enhances both Type I (superoxide anion) and Type II (singlet oxygen) ROS generation under ultrasound. These dual-mode ROS then oxidized the porphyrin ring, forming unstable peroxide intermediates whose decomposition released chemical energy *via* chemiexcitation, producing an exceptionally long afterglow lasting 20 min. This stage-3 dominance was enabled by supportive modifications in other stages. The reduced ΔEST and increased SOC (Stage 2) prolonged the triplet exciton lifetime (9.66 ms vs. 8.80 ms for PpIX), while suppressed π–π stacking (Stage 4) raised the fluorescence quantum yield from 0.22% to 1.13%. As a result, these structure–activity parameters including narrowed bandgap, enhanced charge transfer, increased intersystem crossing probability, and suppressed nonradiative decay collectively yielded an *in vivo* afterglow SBR of 48.6 and effective sonodynamic therapy (SDT) inducing ferroptosis and apoptosis, with simultaneous self-degradation for reduced long-term toxicity 114.

Second, direct intramolecular “bridge” pathways can be introduced to minimize energy loss. This is exemplified by the NPs-Ce4-SN probe. Sonosensitizer-generated singlet oxygen oxidized the thiophene moiety on the SN dye to form a high-energy thiophene-dioxane intermediate. This intermediate then transferred its chemical energy directly to an adjacent SN molecule *via* CIEEL, producing NIR-II afterglow. The same CIEEL principle was applied in the polymeric nanochemiluminescence (PNCL) probe, where singlet oxygen acted as an “intermediary” to oxidize the phenoxy-dioxetane precursor, forming an unstable phenol-dioxetane intermediate that initiates a near-infrared chemiluminescence decomposition process 47. Likewise, in TD nanoparticle systems, ROS-oxidized dioxetane or TD-^•^OH intermediates transferred their chemical energy to nearby intact or decomposed TD molecules to yield luminescence.

Third, intermolecular energy-transfer circuits can be engineered within nanoparticle assemblies, where the final emitter is determined by spectral overlap. In sonoafterglow nanoparticles (SNAPs), spectral overlap between the emission of the oxidized substrate and the absorption of the initiator favored FRET, allowing the initiator to serve as the terminal emitter, as exemplified by RB/DPAo SNAP. When the spectral overlap was limited (*e.g.*, in NCBS/MEHPPV SNAP), the substrate remained the dominant emitter. Linker structure provides an additional means of regulating intramolecular energy transfer and the stability of emissive intermediates. In a ruthenium-based hybrid probe, a non-conjugated linker produced stronger sonoafterglow emission than its conjugated analogue, an effect attributed primarily to slower degradation of emissive intermediates 115. The emitter microenvironment can also be adjusted independently of ROS generation. Doping HBA-COOH into PFODBT nanoparticles altered the energy levels and increased luminescence intensity by 121.4-fold by tuning intrinsic radiative pathways 17.

Enhancing ISC and CRET improves afterglow nanoprobe performance. The FD970/FZ970 nanoprobe illustrated this dual energy-enhancement strategy. Metal coordination in ZnPc promoted ISC and thereby increased ¹O₂ generation under ultrasound irradiation. Covalent donor–acceptor coupling and spectral overlap facilitated CRET, directing the resulting chemical energy into NIR-II afterglow emission. FD970 served as a nonresponsive control comprising the afterglow substrate FDA and the sonosensitizer ZnPc. FZ970 was a dual-lock-activatable probe in which FDA was functionalized with an additional phenylboronate group designed to respond to tumor-associated ONOO⁻. FDA itself contained a chemiluminescent donor based on an adamantylidene-dioxetane scaffold derived from Schaap’s chemiluminescence skeleton and a NIR-II acceptor based on a BBTD core. Metal coordination in ZnPc enhanced ISC, so ¹O₂ was generated efficiently under ultrasound irradiation. In FD970, ¹O₂ directly triggered a [2+2] cycloaddition with the dioxetane precursor. The resulting high-energy dioxetane intermediate then decomposed and transferred energy to the BBTD core *via* CRET, producing bright NIR-II afterglow at ~970 nm. In FZ970, the phenylboronate caging group blocked this process until ONOO⁻ cleaves it, allowing tumor-specific activation. Meanwhile, the ¹O₂ generated during sonication also exerted potent SDT effects 106.

### Mitigation of nonradiative energy dissipation

The final stage determines whether the converted energy emerges as useful luminescence or is lost through nonradiative pathways. Therefore, strategies that rigidify molecular conformations, suppress vibrational relaxation, stabilize emissive states, and optimize aggregation become decisive. Emission stage optimization is particularly important for improving brightness and lifetime at the same time. It is also highly relevant to spectral tuning toward the near-infrared window, because a red-shifted emissive state is only useful if it remains radiatively competitive. In other words, molecular strategies that stabilize the final emissive channel do not merely polish the signal, they often decide whether a biologically detectable signal can exist at all.

Mitigating nonradiative energy dissipation relies on restricting excited-state molecular motion, so that energy is funneled into radiative luminescent channels (**Figure 10D**) 116. Three complementary strategies recur across high-performance systems. First, the use of rigid frameworks or microenvironments restricts molecular motion. For example, CNDs encapsulated within the hydrophobic cavity of ultrasound-induced self-assembled cyclodextrin were confined within a progressively formed rigid framework that constrained molecular vibrations and limited oxygen quenching, thereby stabilizing triplet excitons and yielding ultralong phosphorescence 97. Second, in the aggregation-induced emission (AIE) systems, intramolecular motions (*e.g.*, rotations, vibrations) are restricted in the aggregated state 117, 118, closing nonradiative decay channels and allowing radiative transitions to dominate 119, 120. AIE luminogens (*e.g.*, MeTTh) can undergo ultrasound-promoted assembly, producing enhanced emission through constrained conformation and microenvironment, making them ideal for constructing efficient ultrasound-activated probes or light-harvesting nanosystems 121, 122. Third, nonradiative decay can be suppressed by designing skeletons with specific electronic structures. For instance, BTB ([1,2,5]thiadiazolo[3,4-e]thieno[2″,3″:4′,5′]benzo[2′,1′:4,5]thieno[3,2b]thieno[3‴,2‴:4″,5″]benzo[1″,2″:4′,5′]thieno[2′,3′:4,5]pyrrolo[3,2-g]indole) combined a strong ICT effect with a rigid “V”-shaped structure and served as a conjugated backbone 37. Its dual ICT character suppressed nonradiative transitions and redshifted emission wavelength, while the heterocyclic skeleton promoted intersystem crossing *via* n-π transitions to generate excited triplet states. Together with optimized crystalline packing, this design supported simultaneous high-efficiency fluorescence and phosphorescence, extended ultrasound-excited emission into the NIR region, and achieved high SBR subcutaneous phosphorescence imaging.

The afterglow enhancement of TCL NPs relied on a triple cascade that includes energy generation, energy transfer, and energy dissipation suppression. At the energy generation level, the sonosensitizer TTQ adopted a twisted donor–acceptor architecture with triphenylamine as the donor and quinolinium as the acceptor, which created a strong intramolecular charge transfer effect. Combined with its aggregation-induced emission property, which originated from the propeller-shaped triphenylamine rotor that restricted intramolecular motion in the aggregated state, TTQ exhibited superior ultrasound-triggered reactive oxygen species (especially ¹O₂ production). At the energy transfer level, these ROS oxidized the ONOO⁻-responsive chemiluminescent substrate CL─COOCH₃; the resulting chemiluminescence was then transferred to TTQ *via* chemiluminescence resonance energy transfer. At the energy dissipation suppression level, the AIE property of TTQ was the deciding factor. By restricting molecular rotation and vibration within nanoaggregates, AIE suppressed nonradiative decay and increased the fraction of excited-state energy released through radiative emission, thereby enhancing the afterglow signal. Ultrasound-generated ROS played two roles. They induced the oxidative stress required for SDT and activated the ROS-responsive prodrug DHF to release carbon monoxide. The released CO alleviated tumor hypoxia and sensitized cancer cells to SDT, resulting in a synergistic gas-sonodynamic therapeutic effect 42.

Design principles developed for materials excited by light or X-ray may also inform the development of ultrasound-responsive systems. Rigid confinement restricts molecular motion and can stabilize excited states. Such strategies include incorporation into metal–organic frameworks (MOFs) or covalent organic frameworks (COFs) 123, 124, construction of host-guest networks 125, confinement within protein cavities such as IR-783@BSA 126, 127, use of through-space charge-transfer systems with curved spiro cores 128, and cocrystallization 129. Electronic-structure tuning provides a complementary approach by strengthening spin–orbit coupling or promoting charge transfer, thereby favoring the formation and radiative decay of emissive excited states. Relevant strategies include heavy-atom incorporation 69, donor-acceptor architectures 68, 107, 130, and hybrid metal halides 131.

This section explains how molecular design controls each stage of the ultrasound-induced luminescent cascade. Stage 1 determines whether excitation is efficiently launched. Stage 2 determines whether excitation is efficiently captured. Stage 3 determines how energy is transformed into emissive, persistent, or activatable pathways. Stage 4 determines whether converted energy survives as useful radiative output.

### Integration of ultrasound-induced luminescence with other modalities

Combining ultrasound-induced luminescence with complementary imaging modalities can link distinct energy conversion and delivery pathways. In one approach pairing ultrasound-mediated barrier opening with fluorescence imaging, ultrasound broke ICG-loaded microbubbles into nanoparticles, which may raise the surface-to-volume ratio of ICG and make it more responsive to acoustic energy 132. In a coaxial multimodal configuration, although the transparent ultrasound transducer array (TUT-array) was designed for optical excitation in ultrasound imaging, photoacoustic imaging, and fluorescence imaging, its coaxial structure also supported efficient acoustic transmission and collection of potential sonoluminescence signals when only ultrasound was applied, thereby enhancing optical output from ultrasound excitation 133. Magnetically targeted particles may accumulate in the target region, thereby increasing the local sonosensitizer concentration and enhancing acoustic energy activation to amplify ultrasound-induced luminescence (**Figure 11**) 134.

## Biological performance optimization

Improving the biocompatibility, stability, targeting, toxicity profile, and metabolic fate of ultrasound-induced luminescence probes is essential for *in vivo* translation, as these agents must maintain light emission under repeated ultrasonic excitation while remaining biologically tolerable during circulation, activation, and clearance 72, 135. At the molecular design level, these properties are mainly determined by hydrophilic interfacial engineering, confined coassembly, activatable gating motifs, and degradable or clearance aware architectures.

### Biocompatibility

The molecular design strategy for biocompatibility is to replace the direct exposure of hydrophobic and redox-active luminescent cores with a hydrated, deformable, and biologically buffered interface. In practice, this is usually achieved by PEGylation, DSPE-PEG coating, or lipid encapsulation. For example, in PEG-b-PPG-b-PEG-stabilized SNAPs and DSPE-PEG-coated TD nanoparticles, flexible hydrophilic chains shielded the reactive core from blood, so the key structure–property relation was that interfacial hydration and steric shielding improved aqueous dispersibility and reduced nonspecific adsorption 17, 34. In Lipo@IR780/L012, the lipid bilayer further separated IR780 and L012 from direct plasma contact while preserving co-loading, showing that the improvement in compatibility came not only from increased hydrophilicity but from converting a chemically exposed surface into a buffered nanointerface 96. At a broader level, future biocompatibility design should incorporate protein-corona-aware and low-fouling surfaces, because adsorbed proteins redefined the biological identity of nanomaterials in blood, whereas excessive stealth can compromise subsequent targeting efficiency 136, 137.

### Stability

Confined co-assembly is the key strategy for stability, preserving structural coupling before the probe reaches the lesion. For ultrasound-induced luminescence systems, many functional components are useful when sensitizers, substrates, oxidants, and catalytic units remain packaged together up to the point of activation. During circulation, the main risk is therefore not just aggregation but also premature dissociation, leakage, component exchange, or chemical deactivation in blood 138. In Lipo@IR780/L012/CaO2 32, co-encapsulating IR780, L012, and CaO2 in a single lipid compartment shortened intermolecular diffusion distances and kept them in operative proximity, so the system stayed functional after transport and still responded under insonation. SPNs SP1@CoOOH 39 and CSAPs 139 similarly showed that compartmentalized or hybrid structures can protect ROS related reaction modules under complex biological conditions. At a finer molecular scale, ncRuPA_APN_ is a representative example, because replacing a conjugated alkyne pathway with a nonconjugated amide connection suppressed rapid electron transfer, stabilized the dioxetane intermediate, and prolonged sonoafterglow emission 115.

### Targeting

The molecular design strategy for targeting should be described as a combination of delivery localization and activation localization. In the current field of ultrasound-induced luminescence, one relatively developed route is the design of activatable gating motifs, in which cleavable motifs or quenching groups are incorporated into the luminophore so that the probe remains optically silent during circulation and becomes emissive only in response to disease-associated enzymes, ONOO⁻, or granzyme B 17, 34. The key structure–property relationship is that a trigger responsive group can convert broad probe distribution into spatially selective signal output, even when anatomical delivery is not perfectly precise. This design logic differs from traditional active targeting based on folate, hyaluronic acid, peptides, aptamers, or antibodies, but current nanomedicine studies suggest that combining such ligands with ultrasound compatible probes could further improve tumor localization and cellular uptake in future ultrasound luminescence systems 140, 141. Although ligand-mediated and biomimetic targeting remain underdeveloped in ultrasound-induced luminescence probes, related ultrasound and sonodynamic systems provide useful examples for targeted probe design. Folate-conjugated nanobubbles loaded with low-molecular-weight hyaluronic acid demonstrated folate receptor-mediated targeting of tumor-associated macrophages and ultrasound-triggered delivery for macrophage reeducation 142, whereas macrophage membrane-coated carriers showed biological adaptation and tumor accumulation by inheriting membrane proteins and immune-evasive surface features 143. At the same time, passive delivery still depends on circulation lifetime and the enhanced permeability and retention effect, although this route remains heterogeneous across tumors and patients, which limits delivery reproducibility and motivates the need for more robust multilevel targeting strategies 144-147.

### Toxicity

Activatable gating motifs together with degradable or clearance aware architectures reduce toxicity by limiting both exposure and persistence, although direct probe specific structure toxicity relationships are still not well established. Hydrophilic shells and lipid membranes reduce direct contact between blood and hydrophobic or redox-active cores, which can otherwise promote membrane disruption, nonspecific oxidation, or immune activation during circulation 148. Conditional activation further confines ROS-generating chemistry mainly to pathological sites, suppressing off-target oxidative damage in healthy tissues 17, 34.

### Metabolism

The molecular design strategy for metabolism is to encode clearance into the probe architecture itself through cleavable bonds, degradable backbones, or assemblies that can disintegrate into excretable fragments after function is completed. Efficient clearance is distinct from biocompatibility, as a probe may exhibit favorable circulation behavior yet undergo slow elimination. In many PEGylated or liposomal ultrasound-induced luminescence systems, these formulations primarily improve injectability and short-term biocompatibility, whereas molecular disassembly after the intended function remains a secondary design objective 34, 130. Future probe design could adopt design principles established for renally clearable optical agents, treating small size, minimal nonspecific retention, and rapid renal excretion as primary criteria rather than downstream formulation afterthoughts 149. These structure-property relationship identify molecular weight, hydrophilicity, and labile-bond position as key determinants of metabolic fate. These parameters should be optimized together with luminescence efficiency to balance brightness and clearance.

Overall, ultrasound-induced luminescence probes gain compatibility from hydrophilic interfacial engineering, transport integrity and on-site function from confined coassembly, spatial selectivity and lower off-target toxicity from activatable gating motifs, and long-term safety from degradable or clearance-aware architectures. Together, they connect molecular design and structural control to biocompatibility, circulation stability, targeting, toxicity, and metabolic fate.

## Biomedical applications in various disease models

Ultrasound-induced luminescence has progressed from a physicochemical phenomenon to an optical approach investigated for biomedical use. By pairing acoustic excitation with engineered organic, inorganic, and hybrid systems, ultrasound-induced luminescence allows deep-tissue visualization, high-contrast optical readouts, and controllable activation within defined biological microenvironments. These features have supported its applications to tumor imaging and therapy, modulation of the tumor microenvironment, immune activation, neuromodulation, and other regions. Together, these studies highlight the versatility of ultrasound-induced luminescence in monitoring complex biological processes and, in some cases, regulating them with optical feedback. A comparative performance matrix of representative systems is provided in **Table S2**.

### Tumor microenvironment (TME) visualization

#### Cancer cells

Ultrasound-induced luminescence provides a noninvasive strategy for high-contrast and deep-tissue tumor localization. Representative platforms have been developed to validate ultrasound as an internal energy source for optical imaging. For instance, the small-molecule chemiluminescent probe conjugated PpIX to an enol-ether/dioxetane precursor; focused ultrasound activated PpIX and initiated oxidative dioxetane cleavage, generating sustained and repeatable NIR chemiluminescence (710 nm), and realized US-activated deep tissue imaging (∼20 mm) (**Figure 12A**) 47. The nanoparticle system (NPs-Ce4-SN) with Chlorin e4 (Ce4) and an NIR-II afterglow dye (SN) co-encapsulated into DSPE-PEG carriers emitted long-wavelength afterglow (~1100 nm) with reduced tissue scattering and greater penetration depth (2 cm) (**Figure 12B**) 107. Across *in vitro* assays, tissue-phantom experiments, and murine tumor models (4T1 and MC38), both platforms enabled millimeter-to-centimeter detectability and improved signal-to-background readouts for delineating solid-tumor regions 47, 107. In a separate system (NPs-Ce6), upon ultrasound excitation, the NPs-Ce6 emitted sustained red afterglow light. This light continuously drove photosynthesis in the engineered cyanobacteria PCC 7942, leading to prolonged *in situ* oxygen production that alleviated tumor hypoxia (**Figure 12C**). The improved oxygenation directly enhanced SDT by promoting ultrasound-triggered generation of cytotoxic ¹O₂ from the same NPs-Ce6, while modulating hypoxia-associated signaling (*e.g.*, HIF-1α-related pathways) 150.

Beyond imaging, ultrasound-induced luminescence can function as a controllable *in situ* light source to potentiate photodynamic, photothermal, gas, and immune-modulating therapies. Zhang *et al*. developed AIE nanoparticles in which ultrasound triggered ¹O₂ generation to induce apoptosis of 4T1 breast cancer cells and suppress tumor growth, while intrinsic fluorescence enabled image-guided SDT 118. Zhu and colleagues advanced a dual-function lanthanide-doped nanothermometer (LN-STNP), which acted simultaneously as a microscopic heater and thermal reporter; under mild focused ultrasound, LN-STNPs confined tumor hyperthermia while enabling real-time luminescent thermal readout 151.

To enhance intratumoral penetration, Zhang’s group further designed an ultrasound-chargeable persistent-luminescence nanomotor (mSZ@PDA-NO) composed of SAOE mechanoluminescent dots and ZGC afterglow nanodots coated with an NO-loaded PDA shell. Ultrasound-induced persistent luminescence (696 nm) enabled continuous photothermal heating and NO release, facilitating autonomous motion, deep tumor infiltration, and synergistic photo-gas therapy with marked tumor suppression (**Figure 12D**) 35. In addition, Liu *et al*. developed a sono-bioluminescence transduction platform that integrated the mechanosensitive channel MscL with aequorin to trigger intracellular Ca²⁺ influx and AEQ-mediated bioluminescence, which in turn activated Ce6 for potent ROS generation. Delivered *via* nanoparticle carriers, this approach induced immunogenic cell death, suppressed tumors and metastasis, and generated durable anti-tumor immunity (**Figure 12E**) 111.

Across these studies, ultrasound-induced luminescence served both as a deep-tissue tumor imaging readout and as an internal light source driving several therapies toward solid-tumor treatment.

#### Tumor-infiltrating immune cells

Ultrasound-induced afterglow nanoplatforms have also been applied to visualize the tumor microenvironment, such as the inflammation-associated features. Pu and co-workers developed sonoafterglow SNAPs by integrating singlet-oxygen-producing sonosensitizers with chemiexcitable dioxetanes, generating bright NIR afterglow detectable through deep tissues. Through the incorporation of ONOO⁻-responsive elements, SNAPs selectively illuminated M1-polarized, pro-inflammatory regions within tumors, allowing noninvasive mapping of macrophage activation *in vivo*. Extending this imaging strategy, the team designed a dual-locked sono-immunotherapeutic probe (SCAN) that released the immune agonist R837 and produced ROS when ultrasound exposure coincided with inflammatory stimulation, allowing conditional immunomodulation and optical tracking of TME remodeling (**Figure 13A-B**) 34.

Ultrasound-induced mechanoluminescence has also been used to actively reshape the tumor microenvironment. Zhu and co-workers introduced FUSION, a focused ultrasound responsive immunomodulator-loaded optical nanoplatform, formed by embedding NIR-emitting Zn_1.2_Ga_1.6_Sn_0.2_O_4_:0.5%Cr³⁺ nanoparticles in mesoporous silica and encapsulating the TLR7/8 agonist R848 within a dipalmitoylphosphatidylcholine (DPPC) lipid layer. Once macrophages were labeled with this construct (FUSION-M), their inherent tumor tropism drove targeted accumulation, and focused ultrasound disrupted the lipid shell to release R848 and promote localized M1 polarization. The induced mechanoluminescence reported on macrophage activation in real time, while elevated hydroxyl-radical levels in M1 macrophages amplified the persistent luminescence signal, allowing inflammation to be tracked dynamically. Through this closed-loop mechanism, FUSION shifted the immunosuppressive TME toward an M1-dominant, immune-active state, and enabled macrophage-based immunotherapy with greater spatial precision and an improved safety profile (**Figure 13C-D**) 101. Together, these designs show that coupling localized immune modulation with real-time optical feedback can improve spatial control and provide a direct readout of treatment activity.

#### Lymphatic immune system

Beyond tumor-infiltrating immune cells, ultrasound-induced luminescence has also been investigated for localized immunomodulation and real-time monitoring of immune in deep lymphatic tissues. In one example, ultrasound-responsive nanocomposites combined persistent luminescence with ultrasound-induced mechanoluminescence to regulate immune activity in lymph nodes. The FURIN platform, for instance, accumulated in lymph nodes and gave NIR-II luminescent signals to track nanoparticle delivery. Under focused ultrasound, it released the immunomodulator R837 to activate dendritic cells while emitting mechanoluminescent feedback that reported the onset and progression of immune activation (**Figure 14**) 64. These results supported ultrasound-induced luminescence as a way to combine deep-tissue addressability, spatially confined immune activation, and real-time optical feedback within lymphatic immune tissues.

#### Nervous system

Ultrasound-induced luminescence has recently been expanded into neuromodulation, offering a fiber-free route to deliver optical stimulation to genetically defined neurons. In an early demonstration, Wang and colleagues developed a mechanoluminescent liposomal nanotransducer (Lipo@IR780/L012), a liquid-phase mechanoluminescent nanotransducer designed to generate *in situ* blue light under focused ultrasound excitation. Specifically, ultrasound activated the sonosensitizer IR780 to produce ROS through acoustic cavitation. These radicals then triggered the chemiluminescent substrate L012, producing blue-light pulses aligned with the activation spectra of channelrhodopsin-2 (ChR2) and CheRiff. The authors showed that Lipo@IR780/L012 induced calcium influx and action potentials in opsin-expressing neurons *in vitro.* Following intravenous administration, the nanoparticles enabled noninvasive activation of motor cortical neurons *in vivo* and produced quantifiable, reversible limb movements upon transcranial ultrasound stimulation (**Figure 15A**) 96. Building on this proof of concept, the same group later introduced a cascade-amplified design by incorporating CaO₂-based sono-amplifiers into the lipid carrier to enhance local ROS production and alkalinity during ultrasound exposure. This modification increased luminol emission intensity and shortened the rise time to the millisecond scale, making the emission kinetics more compatible with neural excitation. With this upgraded system, the researchers stimulated deeper structures such as the ventral tegmental area, eliciting dopaminergic firing and reinforcing reward-seeking behaviors in behaving mice (**Figure 15B**) 32.

These studies suggested that ultrasound-induced luminescence can support minimally invasive, genetically targetable modulating of superficial and deep neural circuits with high temporal precision.

#### Other regions

*Helicobacter pylori* colonization and biofilm formation complicate both imaging and treatment. Zhou *et al*. developed a theranostic nanoparticle (mSZ-Clr@ePDA) that couples US-propelled motion with US-activated persistent luminescence and targeted antibiotic delivery for imaging-guided bacterial eradication 36. Mechanoluminescent SAOE and persistent ZGC nanodots were deposited onto mesoporous silica cores, which were subsequently loaded with clarithromycin (CLR), and coated with an ePDA shell (**Figure 16A**). Upon US stimulation, SAOE excited ZGC to generate NIR afterglow imaging with minimal tissue autofluorescence, whereas the ePDA layer both quenched luminescence and protected the payload under gastric conditions. Once *H. pylori*-derived phospholipase cleaved the shell, NIR emission was restored and local CLR release was triggered. At the same time, ultrasound-driven propulsion enhanced mucus penetration, promoted biofilm infiltration, and increased gastric-wall retention and epithelial accumulation. These effects together disrupted biofilm and cleared bacteria efficiently while lowering the antibiotic dose required, demonstrating the potential of deep-tissue persistent-emission as a strategy for precise theranostics against *H. pylori*.

Metformin (MET)-induced liver injury remains hard to assess, largely because sensitive and specific imaging tools are lacking. To address this, Song *et al*. developed a dual-activated sonoafterglow probe (DPA-H_2_S) that combined US-induced luminescence with biomarker-responsive activation for targeted hepatic imaging 71. This small-molecule probe linked the sonosensitizer PpIX, to an afterglow substrate and was dual-locked by an H₂S-sensitive moiety and an ¹O₂-responsive electron-rich double bond (**Figure 16B**). Under focused US excitation, PpIX generated localized ROS locally. At injury sites with elevated H₂S, the two chemical inputs sequentially removed these locks and activated sonoafterglow emission. This resulting contrast enabled deep-tissue visualization and localization of MET-induced hepatic lesions. By pairing molecular specificity with ultrasound activation, DPA-H_2_S offered a noninvasive and sensitive platform that could improve diagnosis of drug-induced liver injury.

## Challenges and perspectives

### Fundamental bottlenecks and critical assessment of current strategies

The main bottlenecks of ultrasound-induced luminescence systems remain inefficient energy transduction, limited photon output 152, mechanistic uncertainty 33, 34, 153, and optical loss in biological tissues 154. These limitations mark the line between transformative advances and incremental improvements. Sonoafterglow systems, biomarker-gated probes, and deep-tissue molecular imaging represent more transformative strategies, as they change the activation or readout of ultrasound-induced optical signals *in vivo* 17, 34, 71. By contrast, formulation-level changes usually improve brightness, stability, circulation, or delivery, and are therefore incremental unless they alter the imaging mechanism or biological readout. Reported differences in the literature mainly stem from uncertainty over the emission origin, rather than direct disagreement in experimental results. Different material systems and ultrasound conditions may favor cavitation-related sonochemistry, ROS-mediated excitation, or mechanoluminescence 33, 34, 153, suggesting that the dominant pathway is system-dependent 152, 154.

### Practical limitations and translational barriers

For all their biomedical promise, ultrasound-induced luminescence systems still face practical and translational challenges: limited energy conversion efficiency, signal attenuation in biological tissues, possible background activation, material safety, and clinical translation.

Low ultrasound-to-light conversion efficiency remains a practical barrier for current systems. Reported efficiencies for related two-step energy-conversion strategies are generally below 15%, with substantial losses occurring during the mechanical-to-chemical step before photon emission 152.

Signal attenuation in biological tissues imposes a second limitation. Although ultrasound avoids the need for external optical excitation, acoustic energy can still be attenuated before reaching the target region. In soft tissues, the attenuation coefficient is approximately 0.2–0.5 dB cm⁻¹ MHz⁻¹ 155. Emitted photons subsequently undergo absorption and scattering, further limiting signal escape and detection at depth 154. Background signals may also result from nonspecific probe accumulation, ultrasound activation outside the target region, or microenvironment- responsive luminescence in nontarget tissues. These effects are particularly problematic when imaging small lesions or low-abundance biomarkers. Activatable or dual-locked probe designs may reduce nonspecific activation and improve imaging specificity 71.

Biosafety assessment of ultrasound-induced luminescent probes should encompass biocompatibility, pharmacokinetics, and long-term safety. In addition to short-term cytotoxicity, hemocompatibility, immune activation, inflammatory responses, and material stability require systematic assessment 156. Probe pharmacokinetics are strongly influenced by size and degradability. For example, efficient renal clearance has been reported for nanoparticles with hydrodynamic diameter below 5.5 nm, whereas larger or poorly degradable probes may clear more slowly and accumulate in the liver and spleen 157. Protein adsorption onto probe surfaces can further alter biodistribution, immune recognition, and clearance 158. Repeated administration of PEGylated probes may also induce anti-PEG immune responses and accelerated blood clearance 159, 160. Probe development should therefore prioritize biodegradable architectures, low-toxicity components, standardized pharmacokinetic characterization, and long-term repeated-dose toxicity studies.

Clinical translation will also require scalable synthesis, batch-to-batch reproducibility, and appropriate regulatory considerations. Many probes require multistep synthesis, doped inorganic matrices, polymeric nanocarriers, or supramolecular assemblies, all of which can complicate large-scale production 161. Process variations during scale-up may alter particle size, colloidal stability, acoustic responsiveness, and emission output, with consequent effects on pharmacokinetics, safety, and imaging reliability 161. For systems combining luminescent probes with ultrasound excitation and optical detection, regulatory evaluation will likely need to address nanomaterial-specific characterization, manufacturing quality control, pharmacokinetic assessment, and probe-device compatibility.

Standardized reporting acoustic parameters is necessary for reproducibility, mechanistic interpretation, cross-study comparison, and eventual clinical translation. Frequency, acoustic pressure, intensity, pulse duration, duty cycle, total exposure time, and cavitation status should all be reported 162. Diagnostic imaging settings should also be distinguished from therapeutic ultrasound settings, because the safety constraints and biological consequences can be different. In particular, cavitation-related effects can include microstreaming, microjet formation, changes in cell permeability, and free radical generation, which are relevant to both therapeutic efficacy and off-target safety 163. In parallel, optical outputs such as emission wavelength, luminescence intensity, signal-to-background ratio, afterglow duration, imaging depth, and therapeutic endpoints should be measured under clearly defined and comparable conditions. A minimum reporting framework should therefore include both acoustic input parameters and optical or therapeutic output metrics, enabling comparison across instant ultrasound-induced luminescence, sonoafterglow luminescence, and Off-On ultrasound-switchable systems.

### Future directions and perspectives

Future progress in ultrasound-induced luminescence probes will likely depend on four closely related aspects: artificial intelligence (AI)-assisted molecular design, disease-responsive activation, multimodal imaging capability, and clinically relevant applications, including precision medicine and immunomodulation.

Future development of ultrasound-induced luminescence probes may move from empirical screening toward AI-assisted predictive design. AI-guided design in this field is still underexplored, but recent advances in AI-assisted fluorophore design and fluorescent probe prediction offer useful frameworks 164, 165. By integrating molecular databases, structure-property prediction, and generative design, AI could speed up the discovery of luminophores with optimized optical properties. Coupling AI predictions with acoustic activation assays, optical readout, ROS analysis, and *in vivo* validation may further guide the rational design of next-generation probes.

Another important direction is to move beyond simple ultrasound responsiveness toward activatable or logic-gated probe designs. By integrating ultrasound activation with disease-associated cues, such as enzymes, ROS/RNS, H₂S, acidic pH, hypoxia, or inflammatory signals, these systems may reduce nonspecific background activation and improve molecular specificity. Recent activatable and dual-locked sonoafterglow probes show that this strategy is feasible for disease-specific imaging and immune-related molecular monitoring 71, 72. Such designs may facilitate the detection of small lesions, low-abundance biomarkers, and molecular changes associated with treatment.

Combining ultrasound-induced luminescence with fluorescence, photoacoustic imaging, or MRI can provide complementary molecular, functional, and anatomical information 166. Fluorescence and afterglow imaging provide molecular readouts, photoacoustic imaging supports deep-tissue localization while adding vascular or functional information, and MRI provides high-resolution anatomical context. Multimodal integration can therefore improve lesion localization and longitudinal monitoring, although these benefits require validation against the individual modalities under matched conditions.

Potential translational applications of ultrasound-induced luminescence extend beyond lesion detection to biomarker-guided patient stratification, treatment selection, and longitudinal response monitoring. Probes responsive to enzyme activity, ROS levels, inflammatory status, or immune-cell activation can report molecular features associated with disease progression or therapeutic response. Whether these signals are sufficiently quantitative and reproducible to guide clinical decisions remains to be established. ROS generated during ultrasound can also be coupled to SDT-inspired immunomodulatory strategies, including the induction of immunogenic cell death and combination immunotherapy 167, 168.

## Conclusion

Ultrasound-induced luminescence has been investigated for deep-tissue optical imaging and therapy. Its three principal paradigms address different functional requirements: instant ultrasound-induced luminescence provides signals during insonation, sonoafterglow extends emission after ultrasound cessation, and Off-On switching luminescence designs restrict activation to defined biological conditions. Further development is constrained by ultrasound-to-light conversion efficiency, attenuation of acoustic and optical signals in tissue, nonspecific background activation, and unresolved issues involving safety, pharmacokinetics, reproducibility, and regulation. Future work should address these limitations through experimentally validated molecular design, including AI-assisted approaches, activatable probes, and integration with complementary imaging modalities. Evaluation in clinically relevant models will be necessary to establish their utility for precision imaging and targeted therapy.

## Supplementary Material

Supplementary tables.

## Figures and Tables

**Figure 1:**
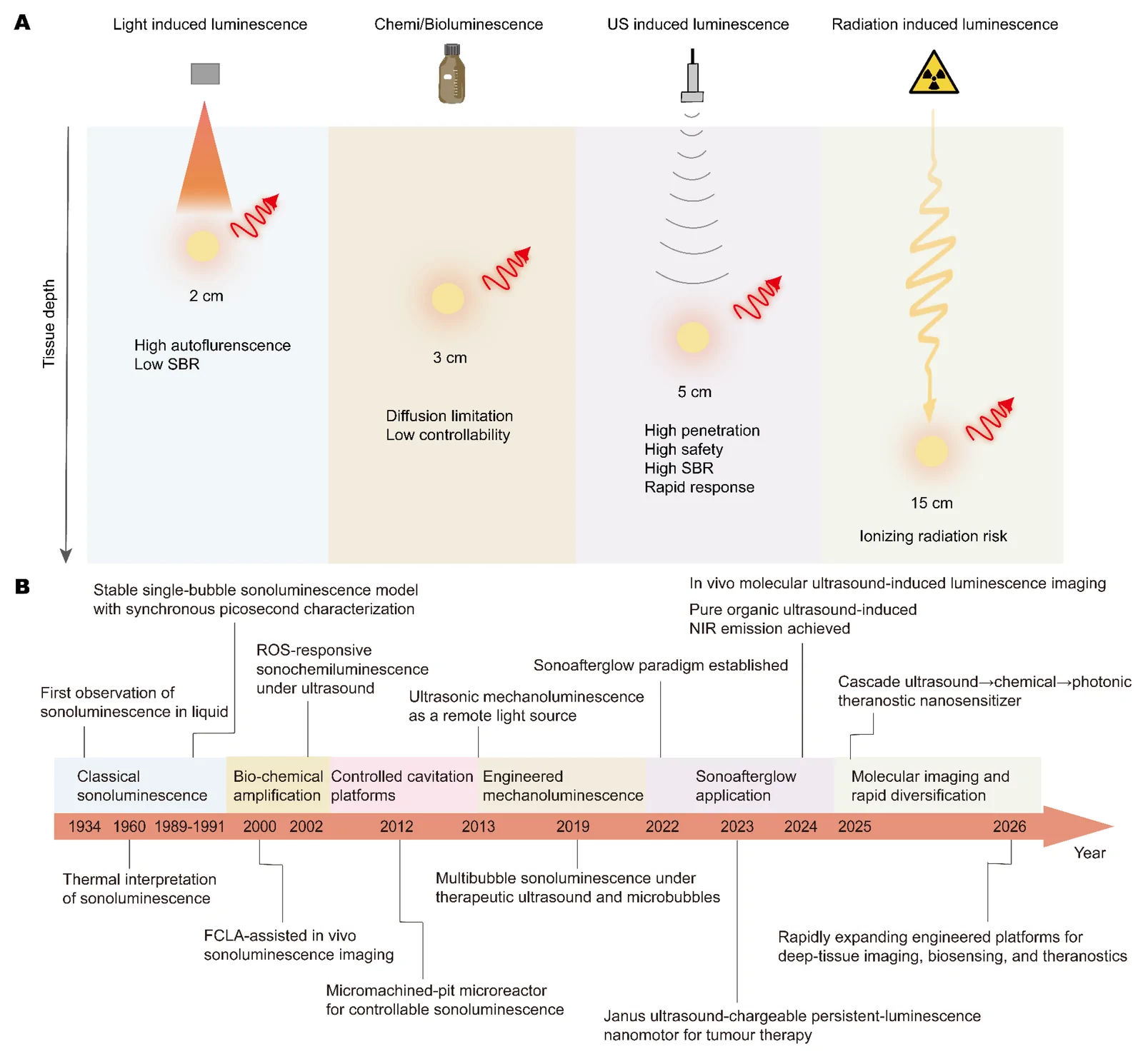
** A. Schematic comparison of different excitation modalities for deep-tissue luminescence imaging**. Light induced luminescence is limited to superficial tissues (~2 cm) and suffers from low SBR due to tissue autofluorescence. Chemi-/Bioluminescence eliminates external excitation noise but is constrained by substrate diffusion (~3 cm) and lacks precise spatiotemporal controllability. Radiation-induced luminescence reaches the greatest depth (~15 cm) but carries safety risks from ionizing radiation. Ultrasound-induced luminescence, by contrast, bridges these gaps, combining deep tissue penetration, high safety (non-ionizing excitation), high SBR (low background), and rapid responsiveness, which makes it well suited to controlled deep-tissue bioimaging. **B. Timeline of the history and milestones of ultrasound-induced luminescence.** Created with Adobe Illustrator CC. SBR, signal-to-background ratio.

**Figure 2:**
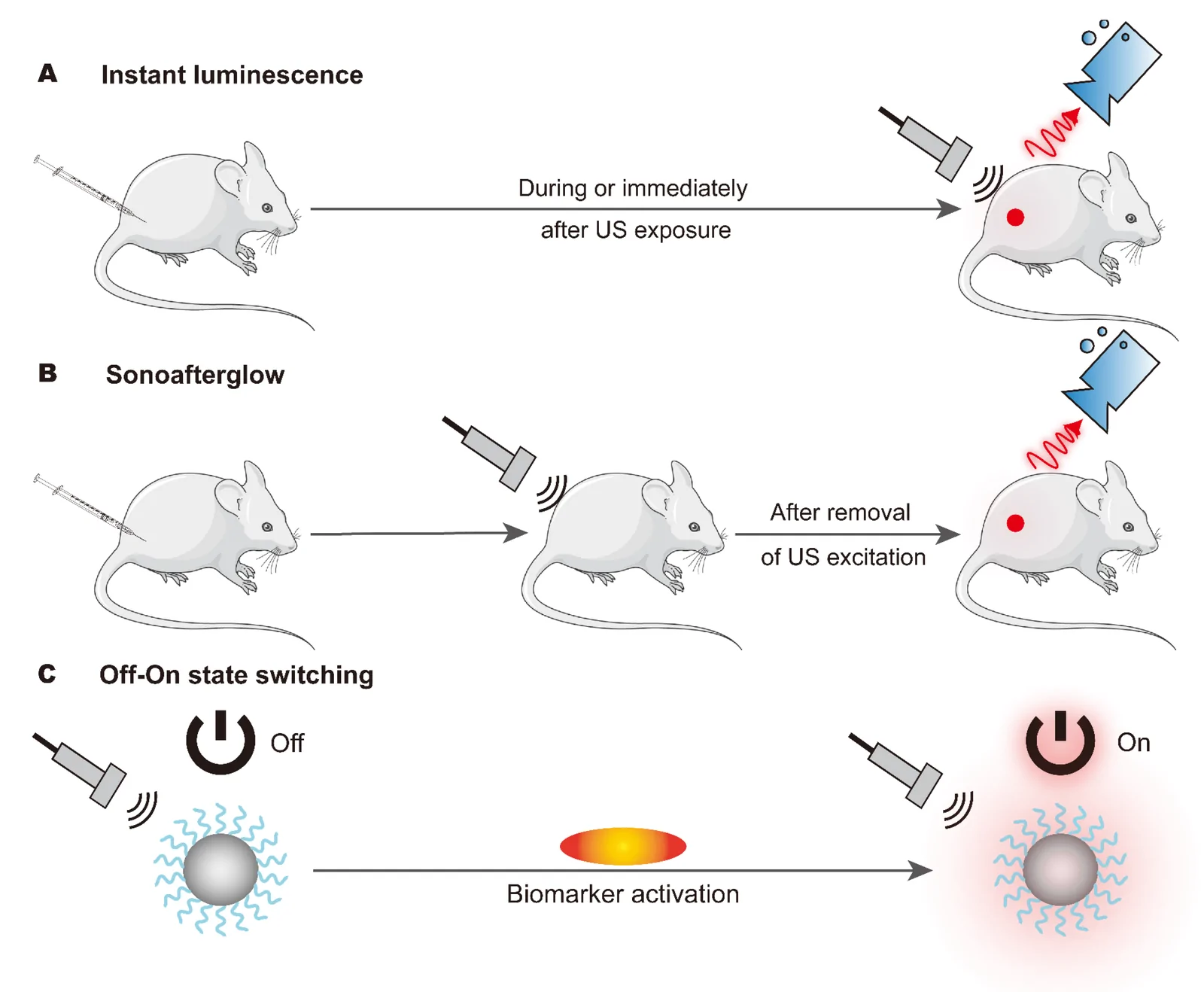
** Paradigms of ultrasound-induced luminescence. (A)** Instant ultrasound-induced luminescence, real-time light emission during or immediately after ultrasound stimulation. **(B)** Sonoafterglow, delayed light emission after the removal of ultrasonic excitation. **(C)** Off-On switching luminescence remains in the “Off” state until exposure to its target biomarkers and turns to the “On” state to activate luminescence. Created with Adobe Illustrator CC.

**Figure 3:**
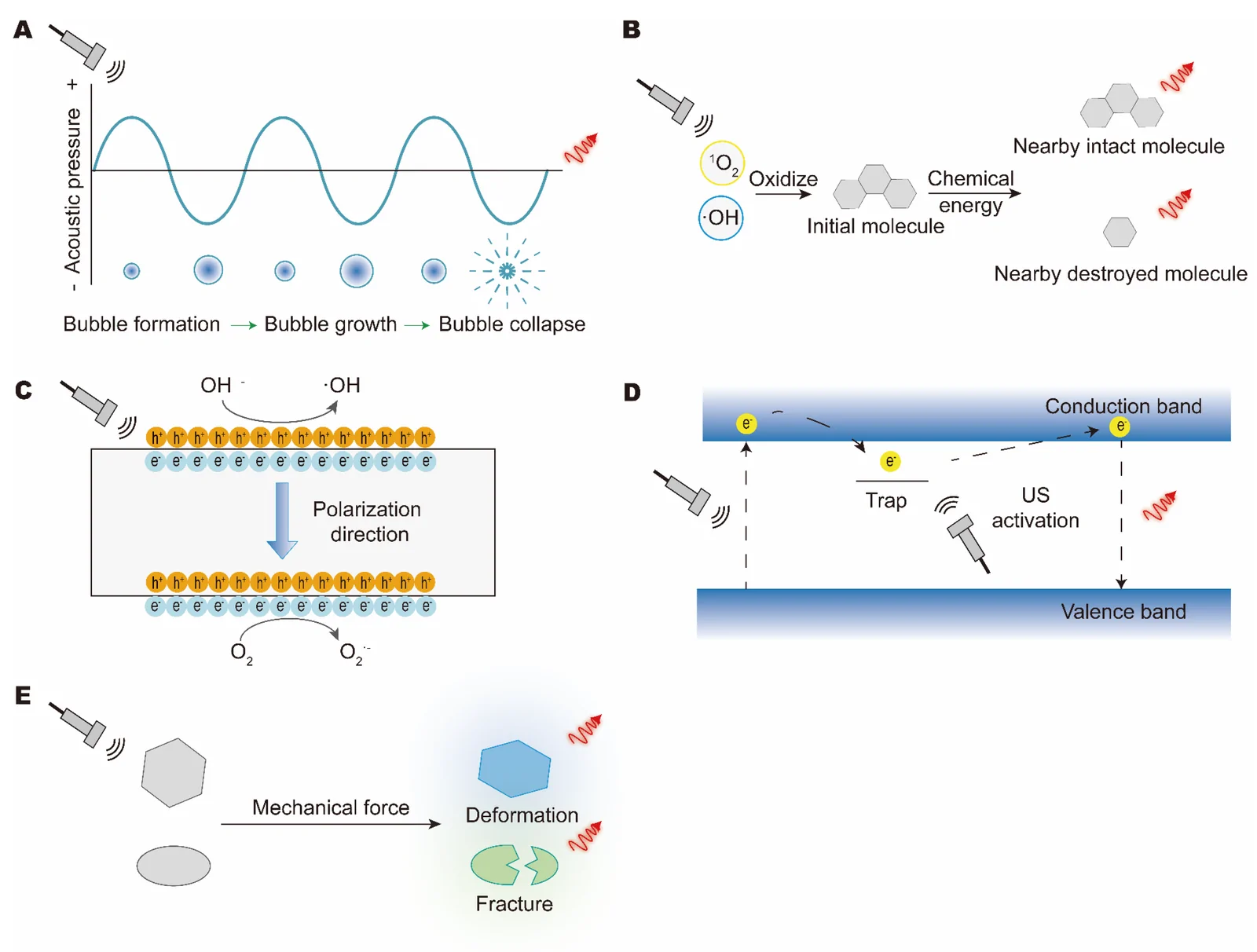
** Mechanisms for instant ultrasound-induced luminescence. (A)** Immediate photon emission *via* bubble dynamics from the ultrasonic cavitation effect.** (B)** Sonogenerated ROS (*e.g.*, ¹O₂, ^•^OH) from reactive species generated during cavitation and piezoelectricity activate chemiluminescence. **(C)** The piezoelectric effect generates ROS to oxidize precursors. **(D)** Mechanical effect releases the electron trap for photon emission. **(E)** Mechanical forces induce instant mechanoluminescence through deformation or fracture. Created with Adobe Illustrator CC.

**Figure 4:**
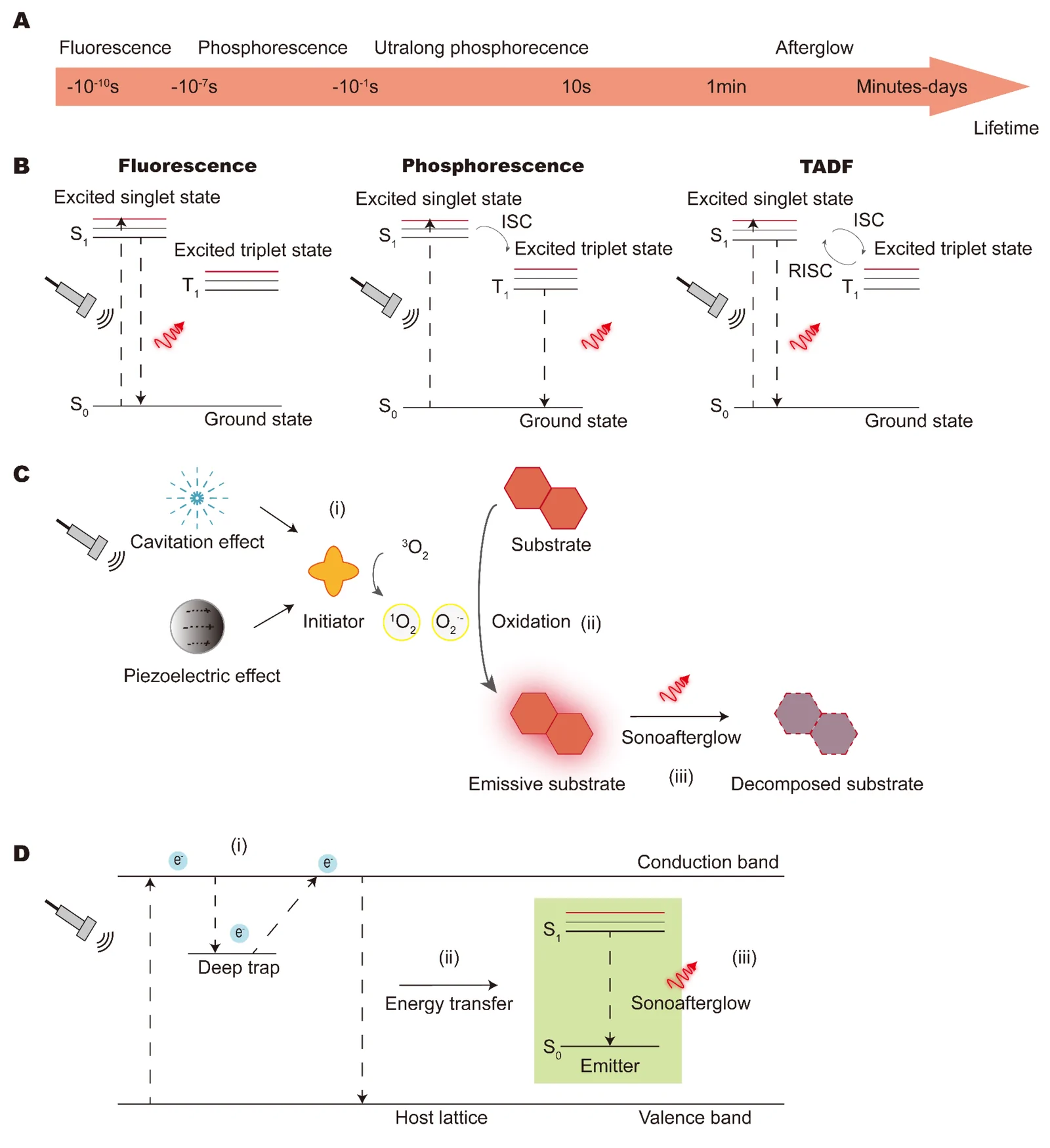
** Mechanisms of sonoafterglow luminescence. (A)** Schematic timeline comparison of luminescence lifetimes, categorizing emission modes from transient fluorescence (10^-10^–10^-7^s) and phosphorescence to long-lived ultralong phosphorescence and afterglow (minutes to days). (**B**) Following ultrasound activation, the depopulation of excited states occurs through three primary radiative pathways: prompt fluorescence from direct S₁ to S₀ transition, phosphorescence from forbidden T₁ to S₀ transition, and delayed fluorescence *via* RISC from T₁ back to S₁. TADF specifically harnesses the latter two long-lived processes, namely the persistent radiative recombination of triplet excitons either as phosphorescence or as recycled singlet emission through thermally activated reverse intersystem crossing, which collectively enable light emission that persists long after removal of ultrasound excitation. **(C)** In organic systems, (i) sonosensitizers absorb ultrasonic energy to generate ROS (¹O₂, O_2_^•-^) through cavitation or the piezoelectric effect. (ii) These ROS subsequently oxidize substrates into emissive intermediates possessing high chemical energy, (iii) whose slow decomposition then produces sonoafterglow emission *via* intramolecular electron transfer. **(D)** In inorganic doped phosphors, (i) ultrasound induces electron promotion from the valence band to the conduction band. (ii) The resultant charge carriers are subsequently captured by defect states within the host lattice, which function as energy traps. (iii) Persistent luminescence ultimately results from the delayed recombination of these de-trapped charge carriers at the emitter centers. Created with Adobe Illustrator CC. S₀, the ground singlet state, S₁, the first excited singlet state, T₁, the first excited triplet state; ISC, intersystem crossing; TADF, thermally activated delayed fluorescence; RISC, reverse intersystem crossing.

**Figure 5:**
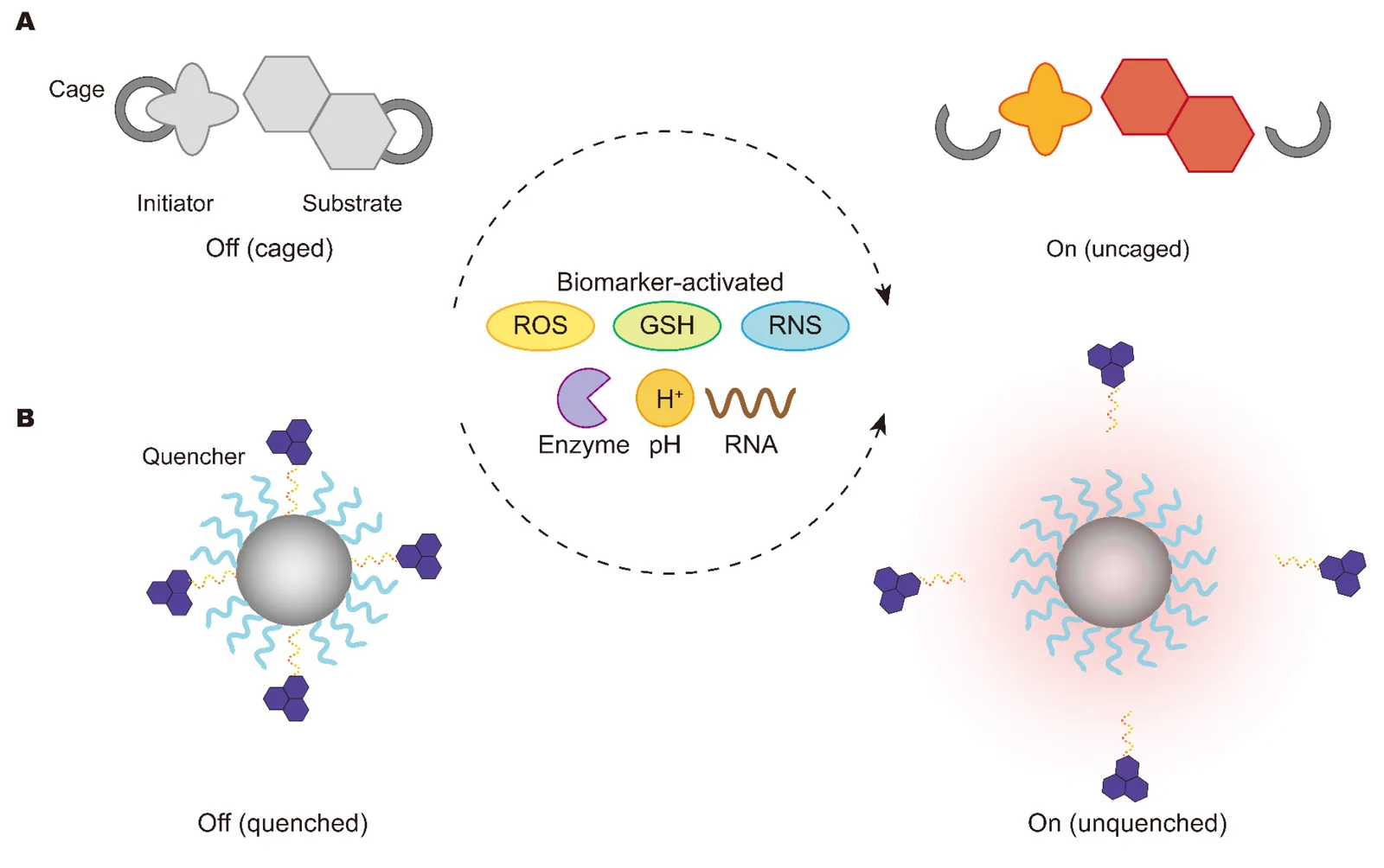
** Logic gates of Off-On switching luminescence. (A)** The initiator or substrate is temporarily caged until exposure to specific environmental biomarkers (*e.g.*, ROS, GSH, RNS, enzymes, or acidic pH). **(B)** The substrate remains quenched until target biomarkers unlock responsive linkers to restore luminescence. Created with Adobe Illustrator CC. GSH, glutathione; RNS, reactive nitrogen species.

**Figure 6:**
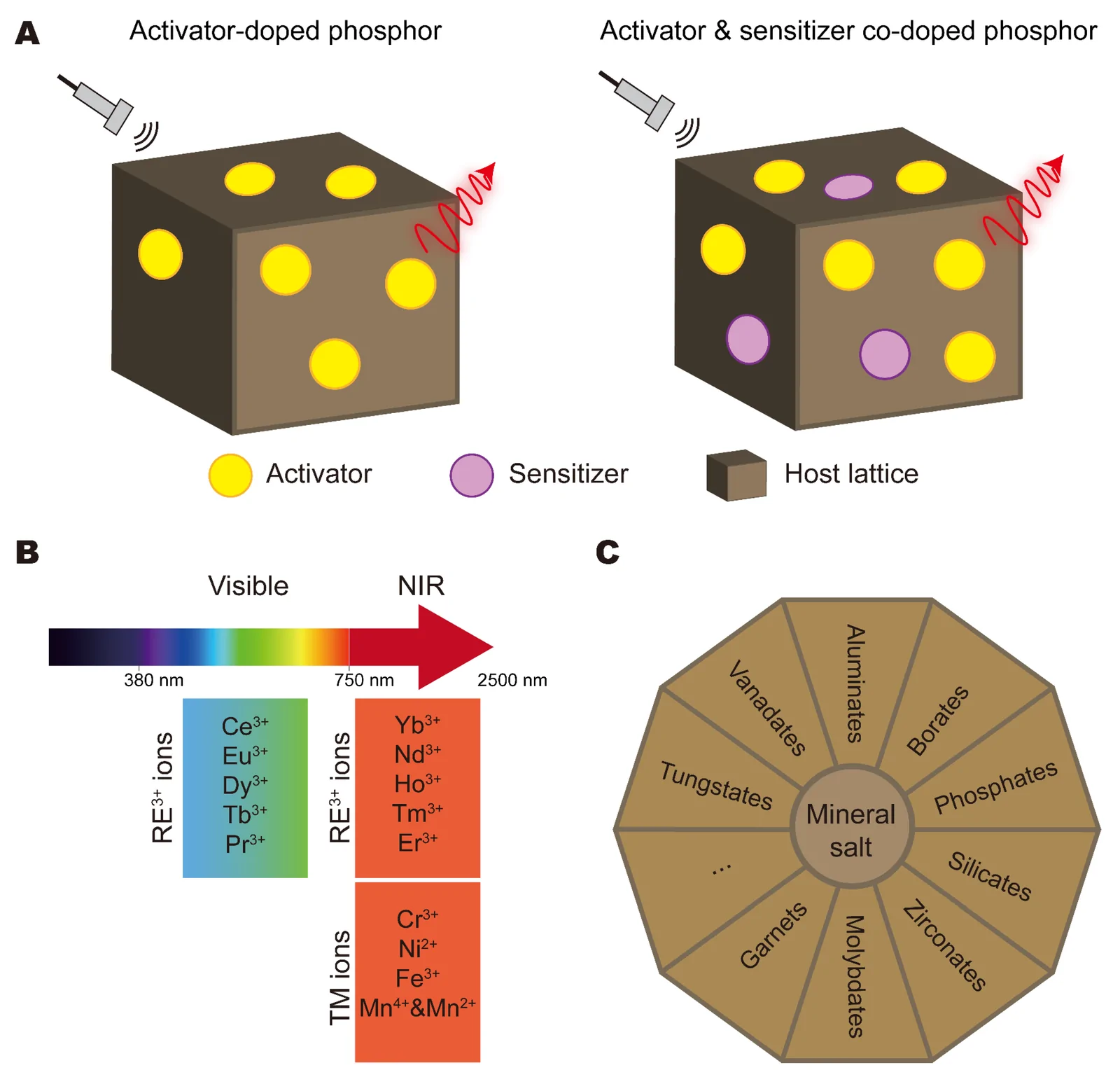
** List of inorganic materials in instant ultrasound-induced luminescence.** (**A**) Schematic illustration of activator-doped and activator-sensitizer co-doped phosphors. The brown cube denotes the mineral-salt host lattice, yellow dots represent activator ions, and purple dots represent sensitizer ions. (**B**) Common RE³⁺ and TM ions used as activators and sensitizers. (**C**) Representative mineral salts used as phosphor host lattices. Adapted with permission from 81, copyright 2025 The Royal Society of Chemistry. Created with Adobe Illustrator CC. RE, rare-earth; TM, transition metal.

**Figure 7:**
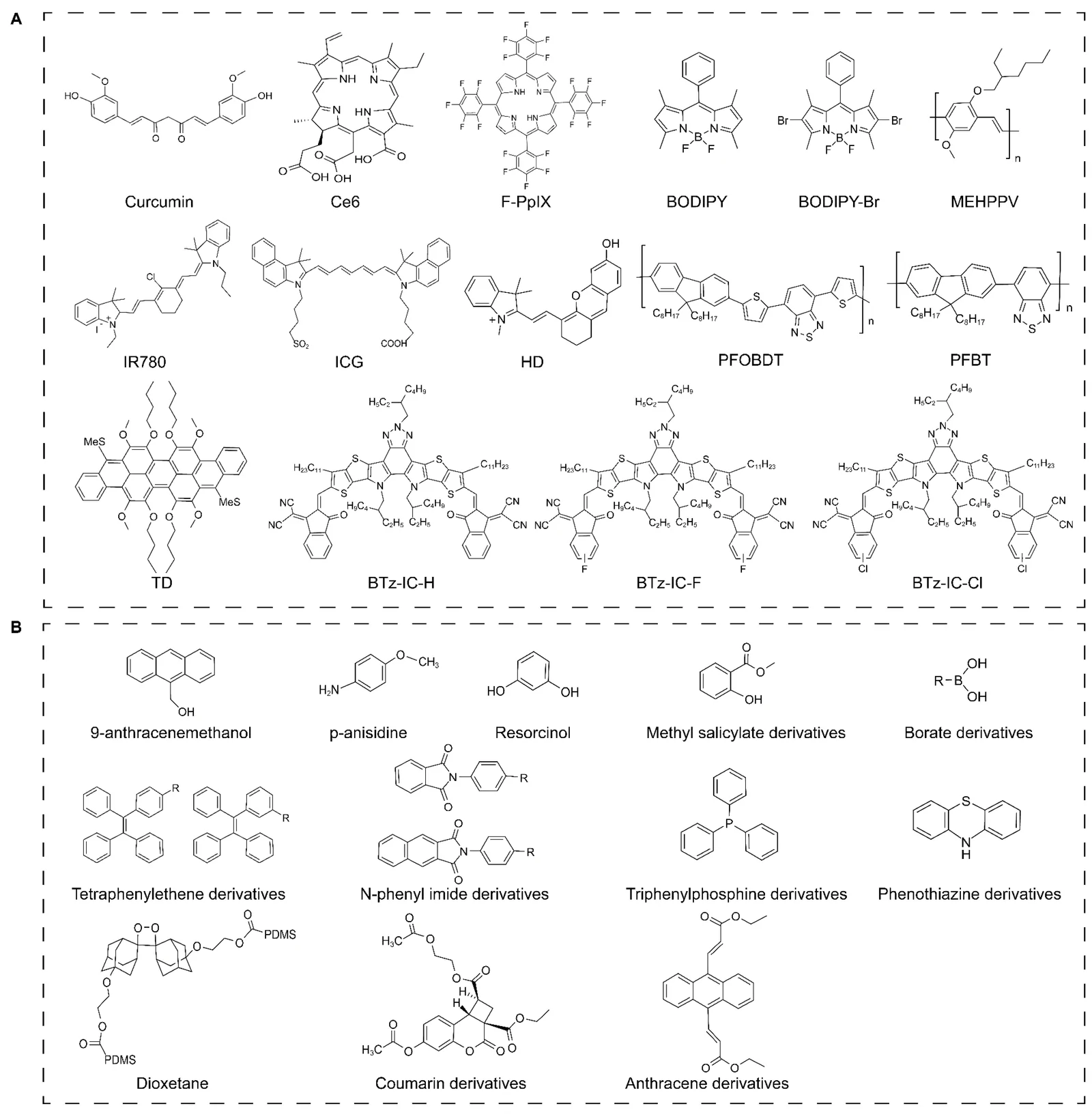
** Representative organic systems for instant ultrasound-induced luminescence.** (**A**) Molecular structures of typical organic luminophores used in nanoparticle systems, including porphyrins, BODIPY, cyanine, TD, and A–D–A′–D–A conjugated molecules. (**B**) Molecular structures of molecular crystals of various organic mechanoluminescent materials (*e.g.*, borate, tetraphenylethene, and coumarin derivatives). Created with Adobe Illustrator CC and KingDraw software. TD, trianthracene derivatives; BODIPY, boron-dipyrromethene.

**Figure 8:**
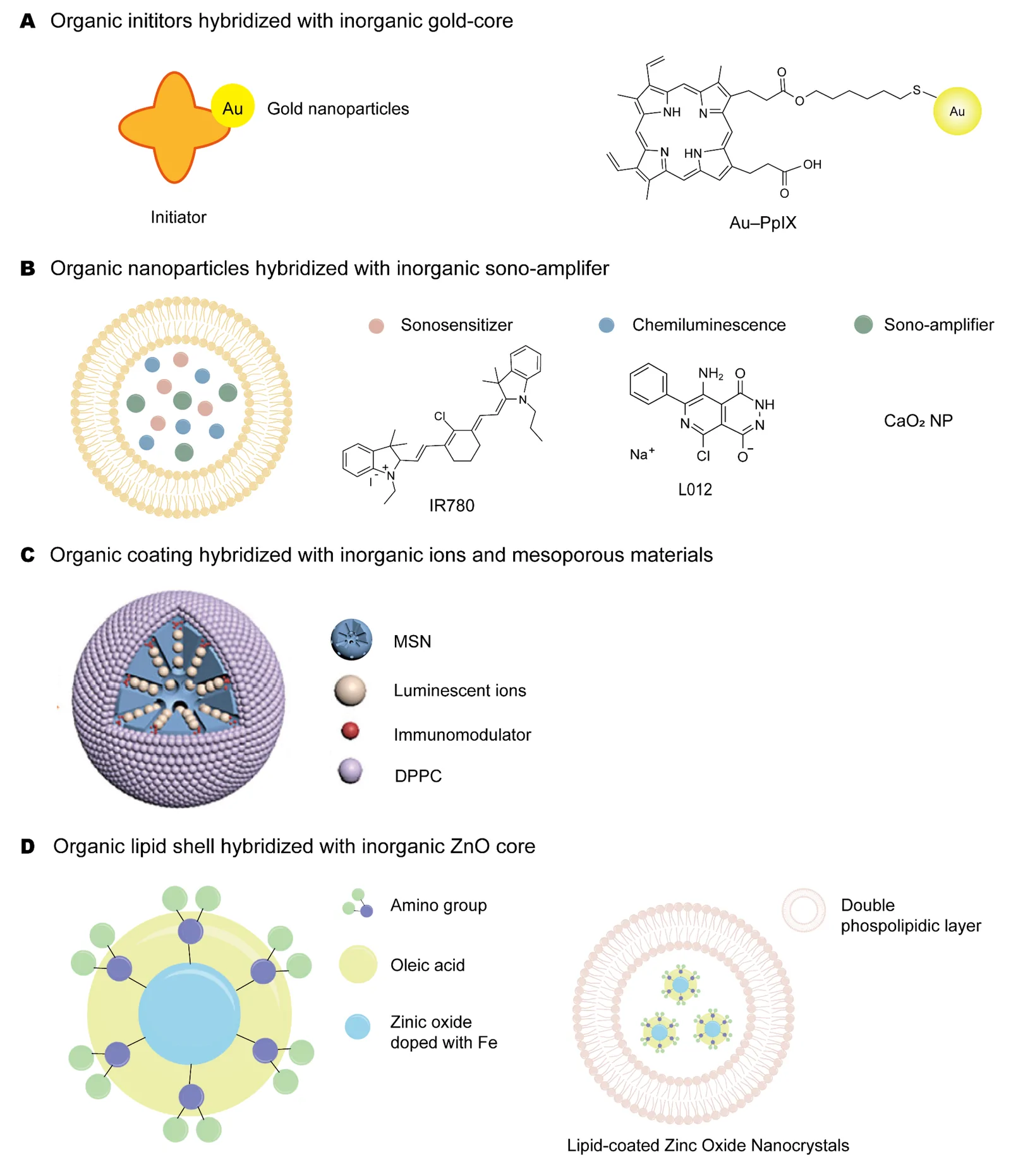
** Hybrid systems for instant ultrasound-induced luminescence.** (**A**) Au–PpIX conjugate linking gold nanoparticles and protoporphyrin IX. (**B**) Nano-hybrid co-encapsulating L012, IR780, and PEG-coated CaO₂. (**C**) MSN-based hybrids loaded with inorganic phosphors (ZGGO or ZGSO). Adapted with permission from 101, copyright 2024 John Wiley and Sons. (**D**) Core–shell ZnO-Lip hybrid with lipid-encapsulated zinc oxide nanocrystals. Created with Adobe Illustrator CC, KingDraw software and BioGDP.com. PpIX, protoporphyrin IX; PEG, polyethylene glycol; MSN, mesoporous silica nanoparticles; ZGGO, Zn_2_Ga_2.96_Ge_0.75_O_8_:Cr,Nd; ZGSO, Zn_1.2_Ga_1.6_Sn_0.2_O_4_:Cr; DPPC, dipalmitoylphosphatidylcholine.

**Figure 9:**
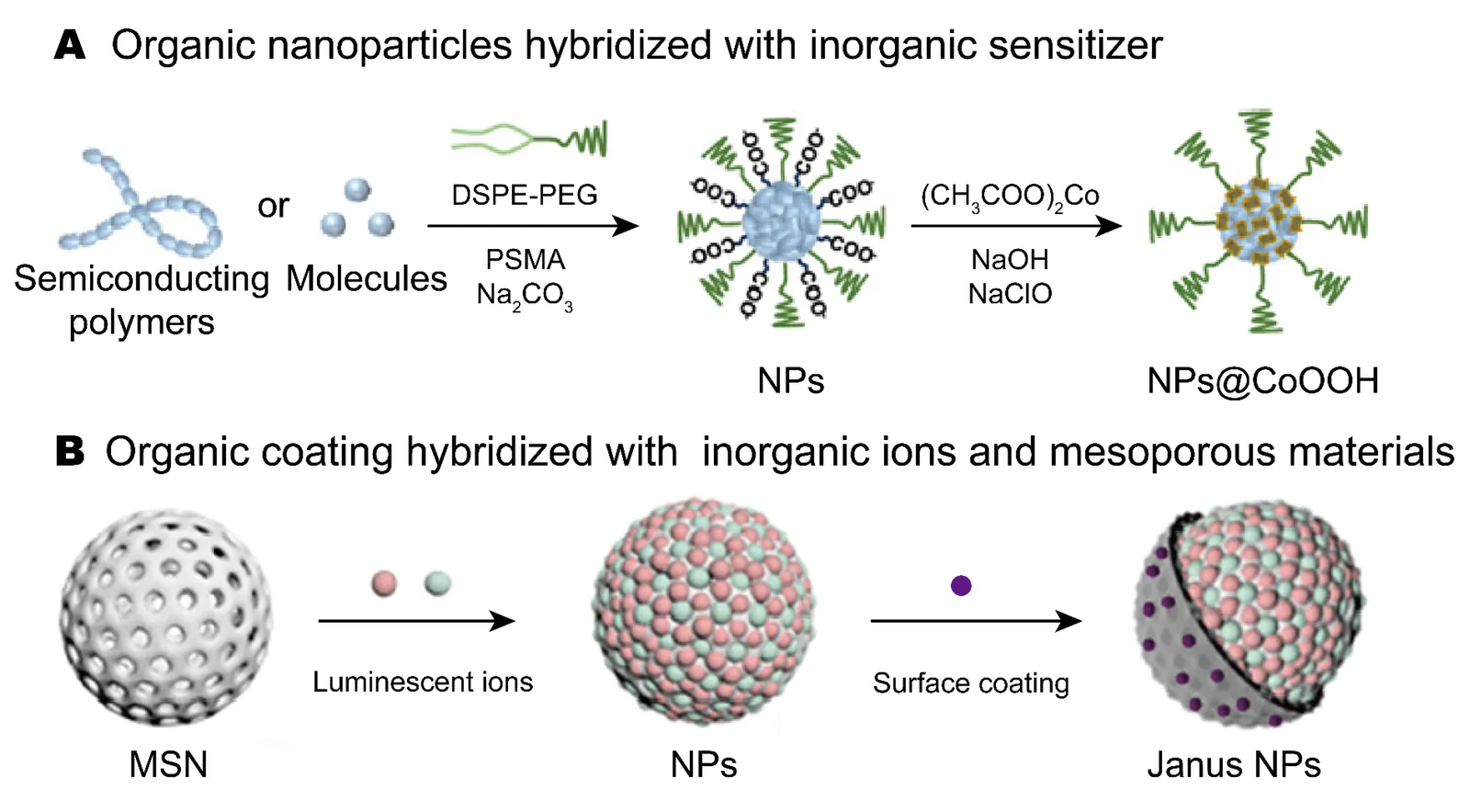
** Hybrid systems applied in sonoafterglow imaging**. (**A**) Organic components with active sites (*e.g.*, semiconducting polymers, cyanines, porphyrins) interface with inorganic sensitizer (CoOOH). Adapted with permission from 39, copyright 2025 American Chemical Society. (**B**) Inorganic mechanoluminescent core–afterglow shell architecture hybridized with organic functional coating. Adapted with permission from 35, copyright 2023 American Chemical Society. Created with Adobe Illustrator CC. NP, nanoparticles; CoOOH, cobalt oxyhydroxide; MSNs, mesoporous silica nanoparticles.

**Figure 10:**
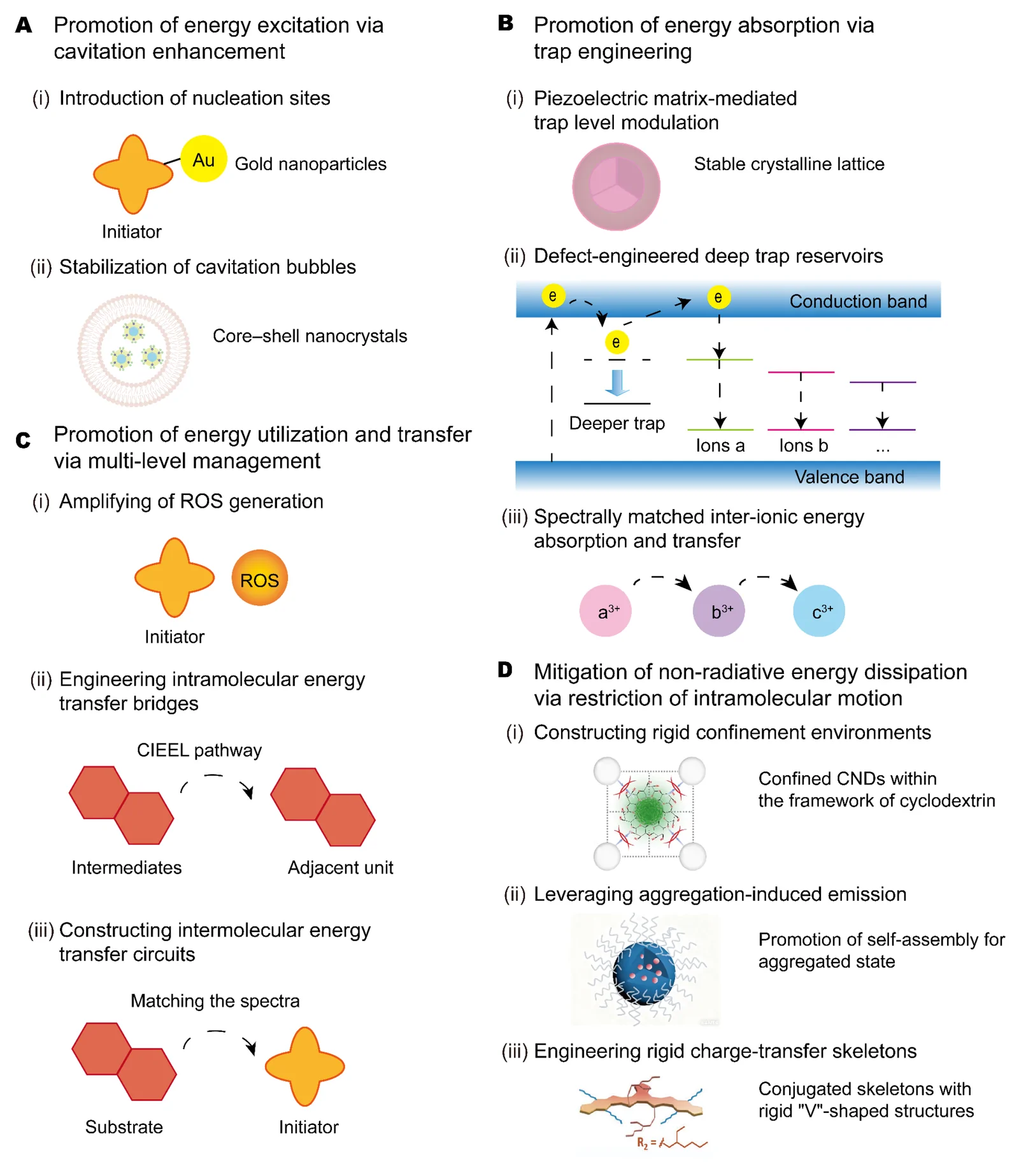
** Optimization of the performance of ultrasound-induced luminescence.** (**A**) Enhancing energy excitation *via* cavitation amplification. (i) Introduction of nucleation sites (*e.g.*, GNPs) to lower the cavitation threshold; (ii) Stabilization of cavitation bubbles using core-shell structures (*e.g.*, ZnO-Lip). (**B**) Promotion of energy absorption *via* trap engineering. (i) Piezoelectric matrix-mediated trap modulation (*e.g.*, CaZnOS); (ii) Defect-engineered deep trap reservoirs for long-term storage (*e.g.*, ZGSO@MSN); (iii) Spectrally matched inter-ionic energy transfer (*e.g.*, ZGGO@MSN). (**C**) Promotion of energy utilization and transfer *via* multi-level management. (i) Amplifying ROS generation or modulating the microenvironment; (ii) Engineering intramolecular energy transfer bridges (*e.g.*, CIEEL); (iii) Constructing intermolecular FRET circuits to tune emission. (**D**) Mitigation of nonradiative energy dissipation *via* motion restriction and electronic optimization. (i) Rigid confinement stabilizing triplet states (*e.g.*, CNDs in frameworks). Adapted with permission from 97, copyright 2023 American Chemical Society; (ii) Leveraging AIE to shut down nonradiative pathways. Adapted with permission from 169, copyright 2024 John Wiley and Sons; (iii) Engineering rigid charge-transfer skeletons. Adapted with permission from 37, copyright 2024 John Wiley and Sons. GNPs, gold nanoparticles; NIR, near-infrared; CIEEL, chemically initiated electron exchange luminescence; FRET, Förster resonance energy transfer; CNDs, carbon nanodots; AIE, aggregation-induced emission.

**Figure 11:**
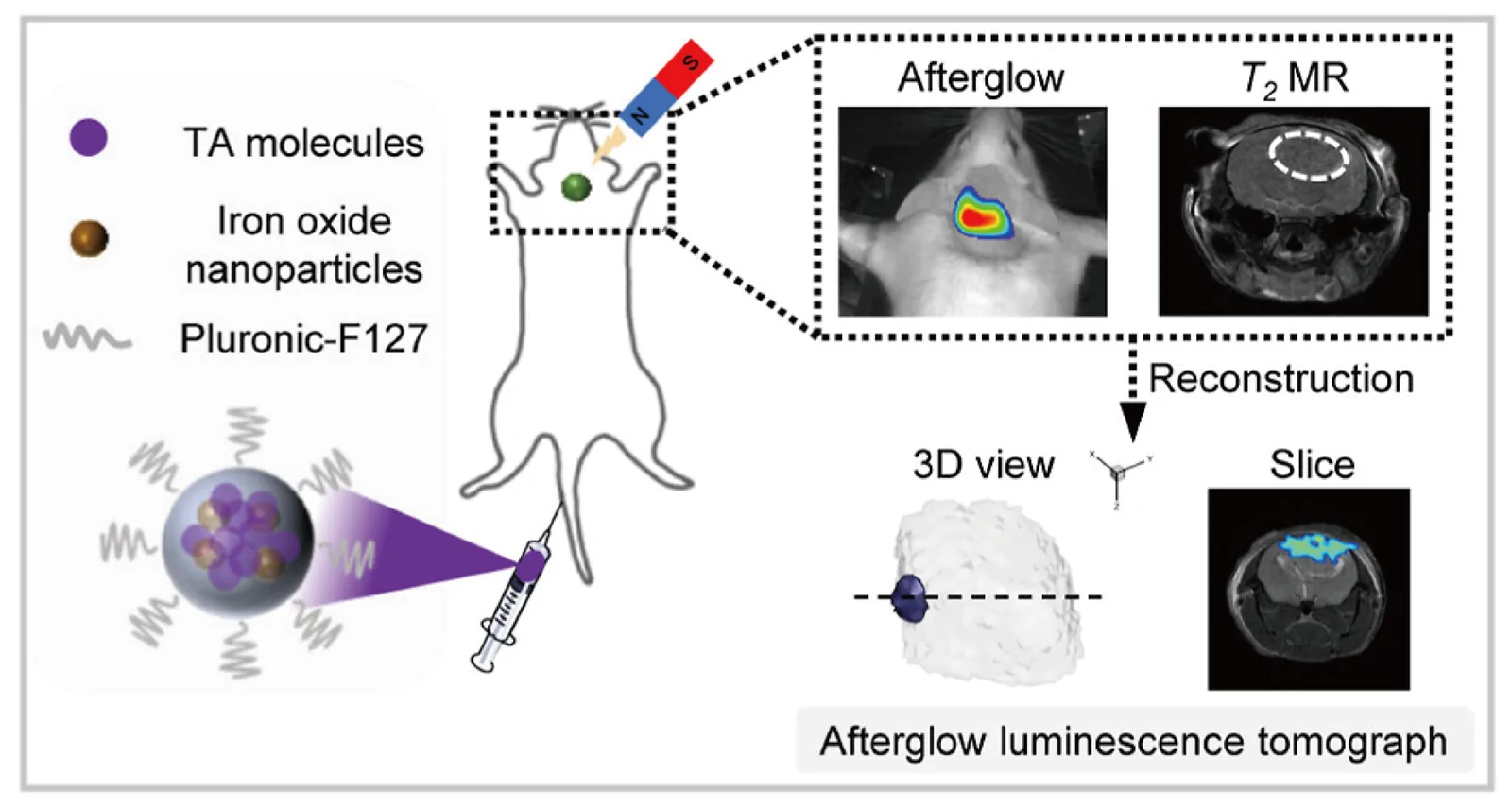
** Integration of ultrasound-induced luminescence imaging with other modalities.** The luminescent-magnetic nanoparticles platform integrates magnetic targeting, ultrasound- and light-induced afterglow, and T₂-weighted magnetic resonance imaging (MRI). The iron oxide cores enable magnetic targeting and MRI, while the co-encapsulated trianthracene (TA) molecules mediate both optical modes. Magnetic enrichment of the nanoparticles at the tumor site increases the local density of TA, thereby enhancing the light-induced afterglow and enhancing ultrasound-induced luminescence through elevated sonosensitizer concentration and energy deposition. Reprinted with permission from 134. Copyright 2025 American Chemical Society.

**Figure 12:**
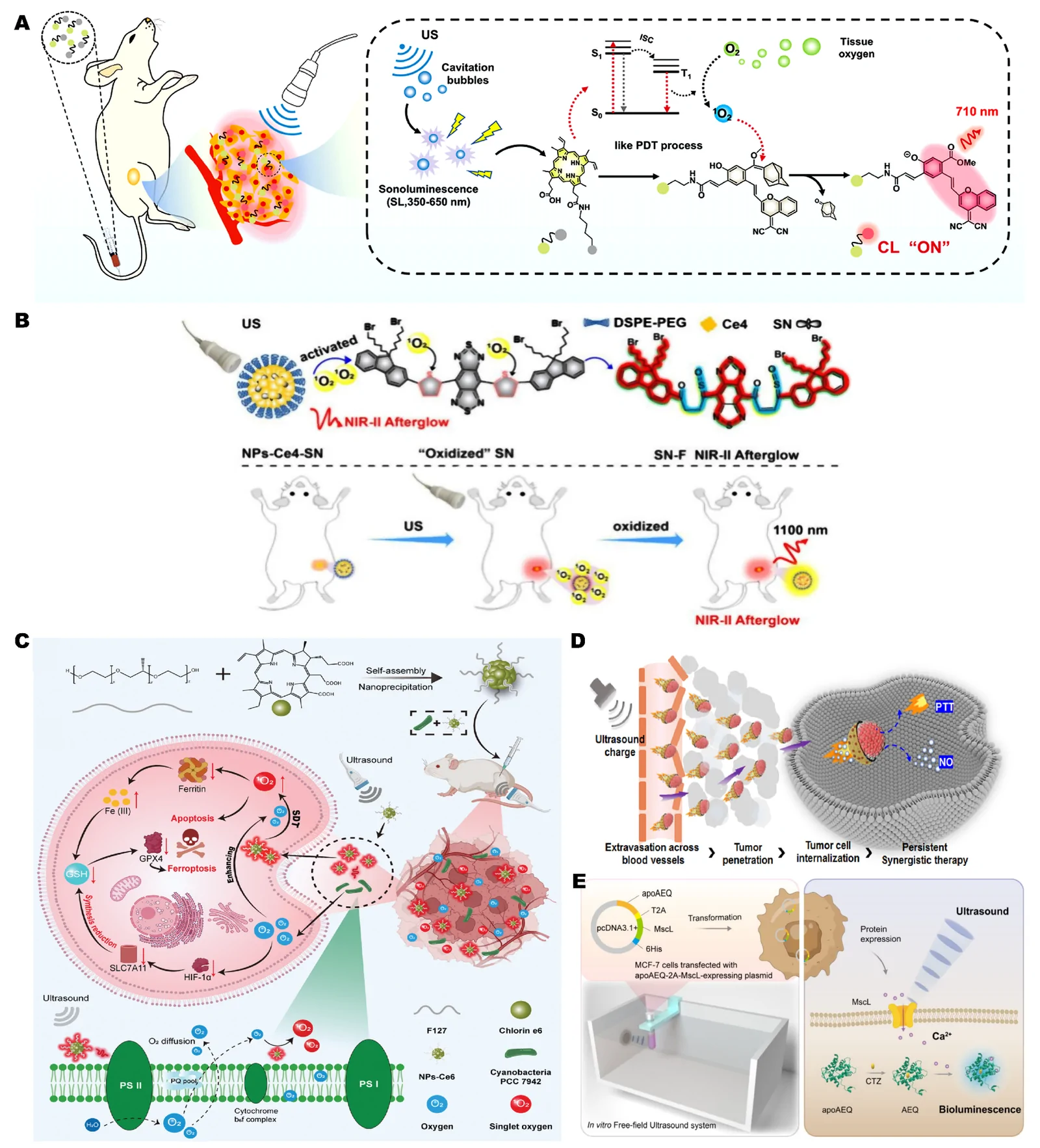
** Applications of ultrasound-induced luminescence in tumors.** (**A**) Schematic illustration of US-activated PNCL for NIR CL imaging of tumor. Reprinted with permission from 47. Copyright 2023 American Chemical Society. (**B**) Schematic display of US-activated NPs-Ce4-SN for *in vivo* NIR-II afterglow imaging. Reprinted with permission from 107. Copyright 2025 American Chemical Society. (**C**) Schematic illustration of sonoafterglow-enhanced photosynthetic oxygenation to boost SDT. Reprinted with permission from 150. Copyright 2024 John Wiley and Sons. (**D**) The internal NIR excitation of PDA caps produced a persistent self-thermophoretic force to propel NP motion, which promoted tumor accumulation, intratumoral penetration, and intracellular uptake of NPs. In the meantime, NIR-stimulated PTT and PL-triggered NO release achieved a synergistic antitumor efficacy. Reprinted with permission from 35. Copyright 2023 American Chemical Society. (**E**) Schematic diagram depicting the luminescence mechanisms of the mechanoresponsive bioluminescent protein when exposed to ultrasound excitation. Reprinted with permission from 111. Copyright 2025 American Chemical Society. PNCL, polymeric nanochemiluminescence; NIR, near-infrared; CL, chemiluminescence; SDT, sonodynamic therapy; PDA, polydopamine; PTT, photothermal therapy; PerL, persistent luminescence; NO, nitric oxide.

**Figure 13:**
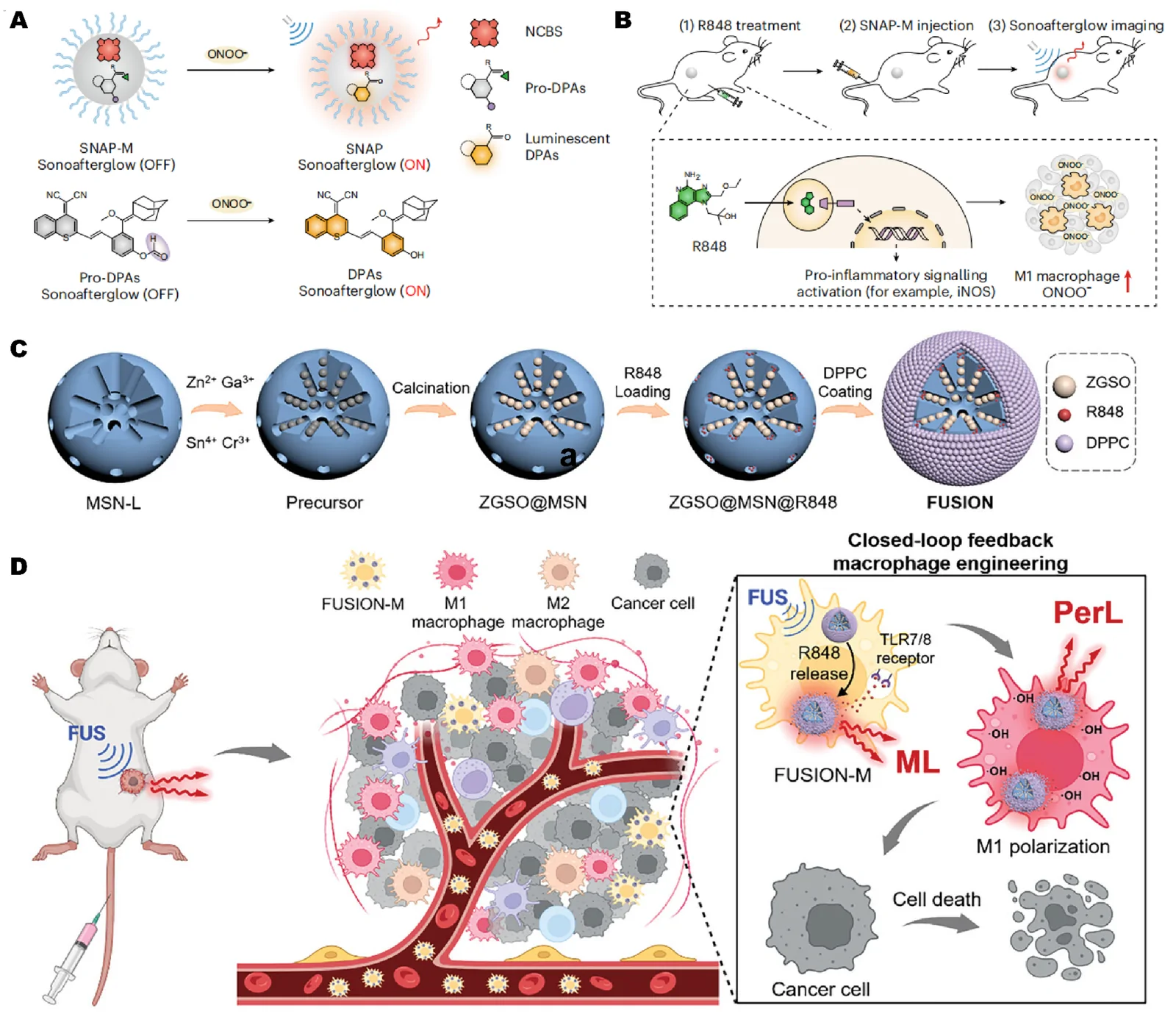
** Applications of ultrasound-induced luminescence in the TME.** (**A**) SNAP-M for activatable sonoafterglow imaging of M1-characterized pro-inflammatory TME, where overproduced ONOO⁻ activated Pro-DPAs. (**B**) R848-induced pro-inflammatory TME monitored by *in vivo* sonoafterglow imaging. Reprinted with permission from 34. Copyright 2023 Springer Nature. (**C**) Synthesis of FUSION: ZGSO embedded in MSNs loaded with R848 and encapsulated by DPPC. (**D**) FUSION-M integrated with macrophages for closed-loop immunotherapy. Reprinted with permission from 101. Copyright 2024 John Wiley & Sons, Inc or related companies. TME, tumor microenvironment; ONOO⁻, peroxynitrite; MSNs, mesoporous silica nanoparticles; DPPC, dipalmitoylphosphatidylcholine; ML, mechanoluminescence; FUS, focused ultrasound; PerL, persistent luminescence.

**Figure 14:**
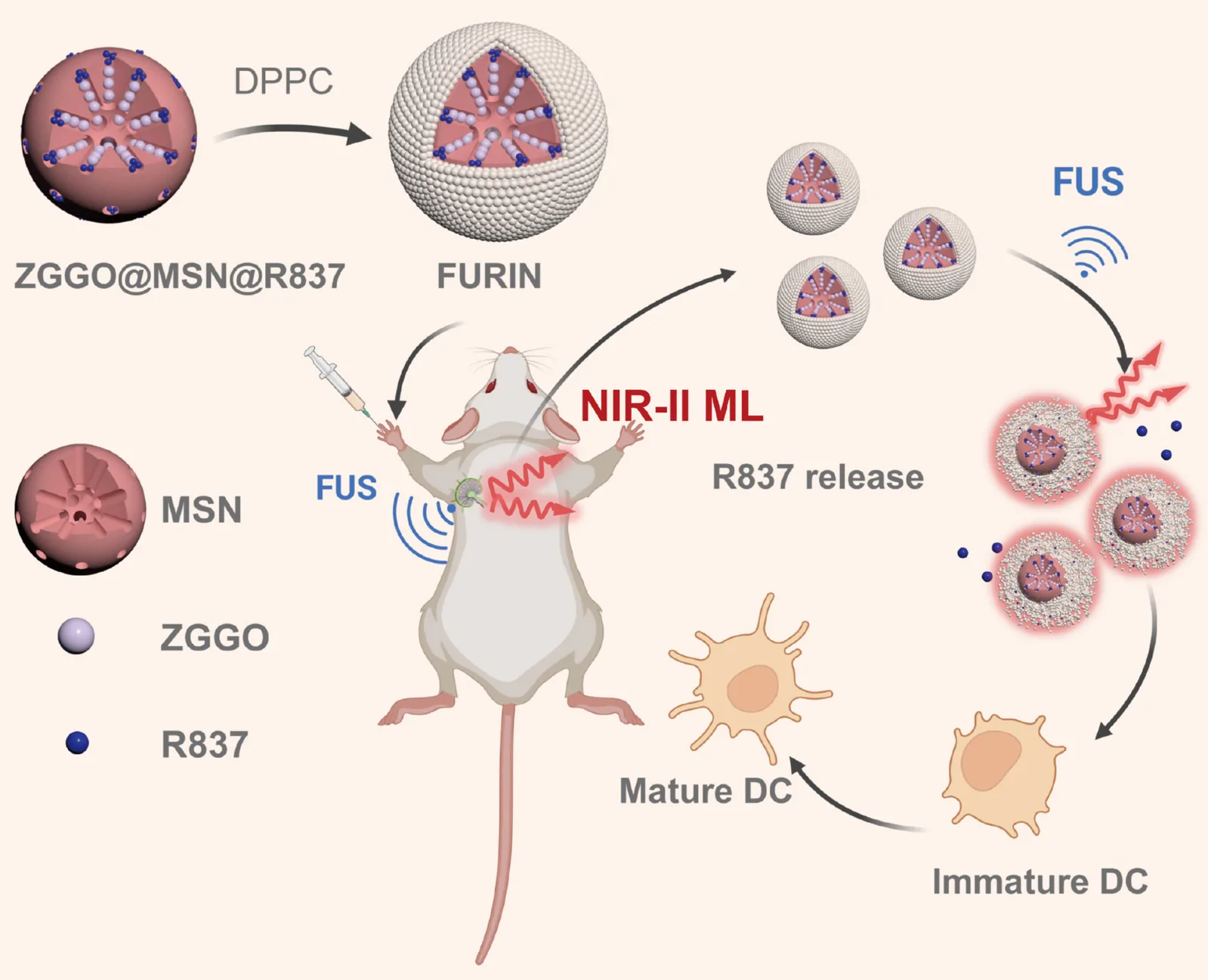
** Applications of ultrasound-induced luminescence in the immune system.** Schematic diagram of FURIN for spatiotemporally controllable immune activation in the lymphatic system *in vivo*. FURIN could be enriched in the lymph node of mice *via* subcutaneous injection, and the mechanoluminescence of FURIN in the near-infrared region enabled real-time, visualized feedback of FUS by bioimaging. FUS with high precision and deep penetration could activate dendritic cells (DCs) *in vivo*. Reprinted with permission from 64. Copyright 2024 Wiley. FURIN, focused ultrasound-responsive immunomodulator-loaded nanomaterial; ML, mechanoluminescence; FUS, focused ultrasound; NIR, near-infrared.

**Figure 15:**
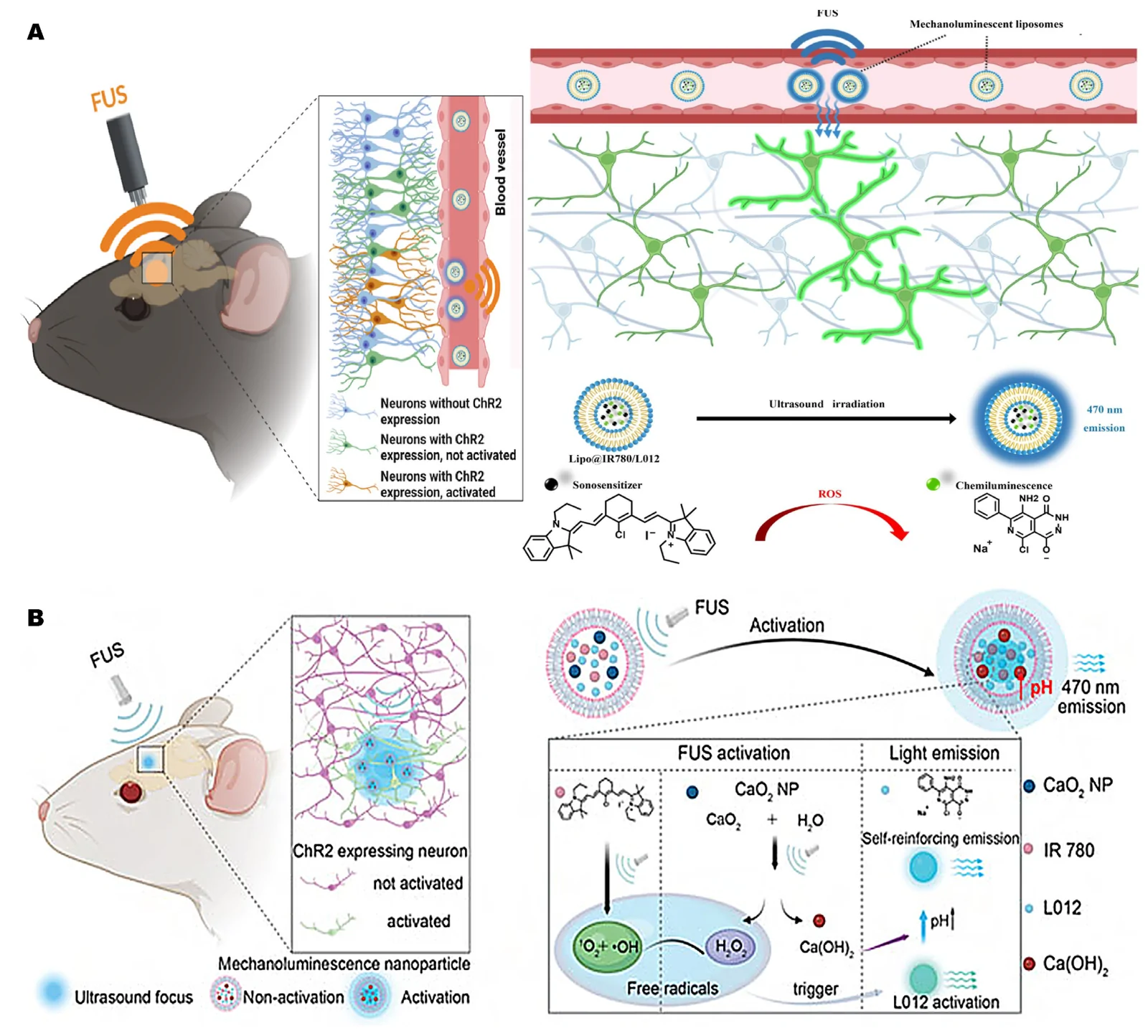
** Applications of ultrasound-induced luminescence in the nervous system.** (**A**) FUS penetrated the skull to activate mechanoluminescent nanotransducers within brain vasculature, generating localized light that selectively stimulates ChR2-expressing neurons while leaving non-expressing cells unaffected. Reprinted with permission from 96. Copyright 2023 American Chemical Society. (**B**) FUS activated a cascade-amplified mechanoluminescent nanotransducer (Lipo@IR780/L012/CaO₂) to generate enhanced blue emission for ChR2 stimulation, enabling wireless deep-brain neuromodulation. FUS-triggered light activation drove reward-related neural activity, increasing lever-press behavior *in vivo*. Reprinted with permission from 32. Copyright 2023 American Chemical Society. FUS, focused ultrasound; ChR2, channelrhodopsin-2.

**Figure 16:**
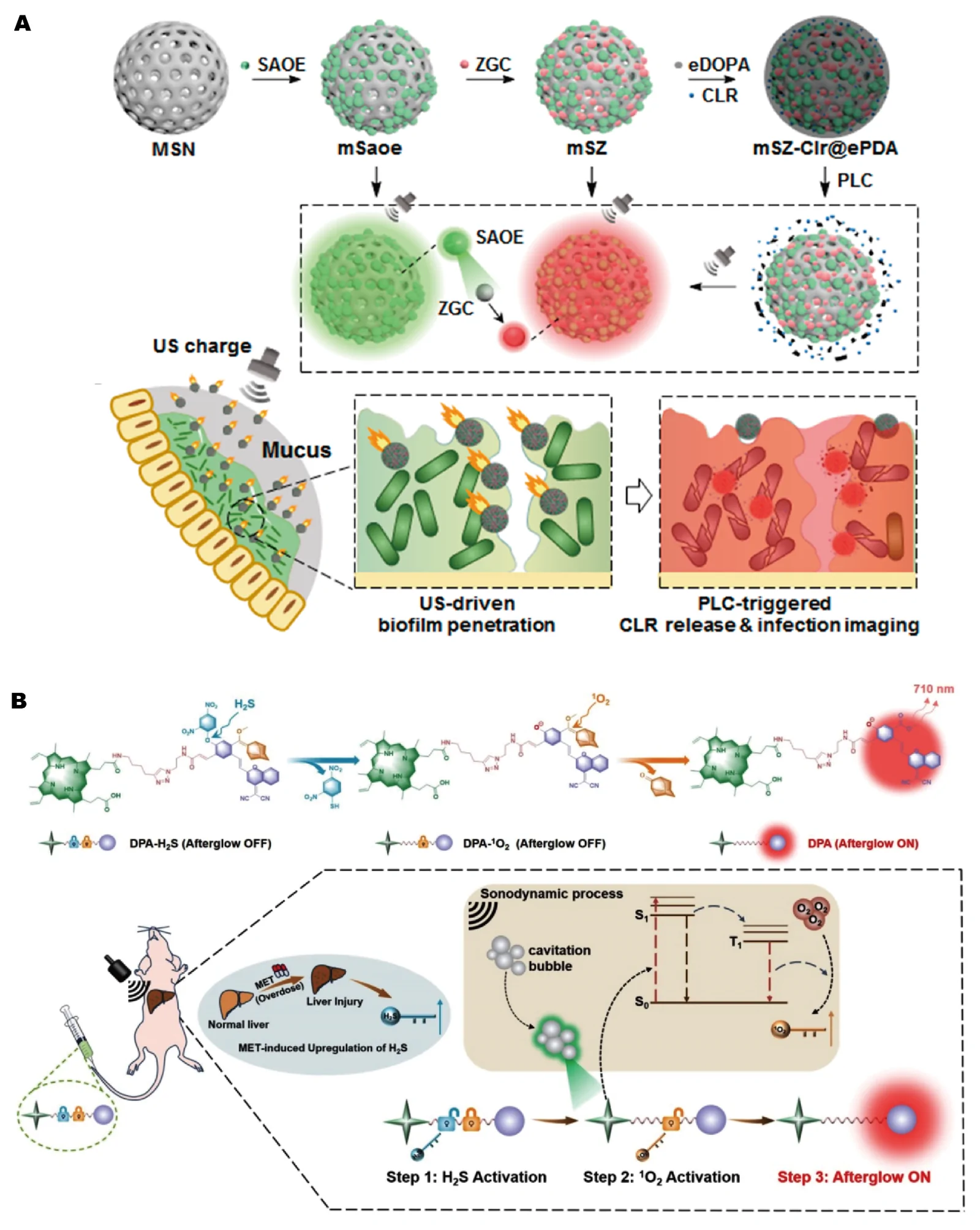
** Applications of ultrasound-induced luminescence in other regions.** (**A**) mSZ-Clr@ePDA NPs for *H. pylori* imaging. US generated green luminescence from SAOE to excite ZGC (red emission). ePDA coating quenched emission until PLC-triggered breakdown restored it. Reprinted with permission from 36. Copyright 2022 American Chemical Society. (**B**) Dual-locked DPA-H_2_S probe for precise imaging of MET-induced liver injury *via* H₂S-activatable sonoafterglow. Reprinted with permission from 71. Copyright 2024 American Chemical Society. SAOE, SrAl_2_O_4_: Eu^2+^; ZGC, ZnGa_2_O_4_: Cr^3+^; MSNs, mesoporous silica nanoparticles; CLR, clarithromycin; ePDA, polydopamine; PLC, phospholipase C; MET, metformin.

**Table 1:** Comparison of the three major paradigms of ultrasound-induced luminescence.

	Instant ultrasound-induced luminescence	Sonoafterglow luminescence	Off-On switching luminescence
Definition	Transient emission during or immediately after ultrasound stimulation	Delayed emission persisting after ultrasound cessation	Biomarker-gated activation followed by ultrasound-triggered emission
Emission timescales	During US excitation	Seconds to minutes or longer after US excitation stops	Determined by switching kinetics and stimulus duration
Energy conversion pathways	Rapid ultrasound-triggered transduction *via* cavitation, piezoelectric, thermal, or mechanoluminescent pathways	Acoustic energy storage in chemical intermediates or trapped states, followed by delayed photon release	Biomarker-gated caging/quenching controls specificity; ultrasound supplies excitation energy
Main biomedical advantages	Real-time imaging and synchronous therapeutic regulation	Reduced background and improved signal-to-noise ratio for delayed imaging	Activatable imaging and triggered drug release with high specificity
Main limitations	Requires continuous ultrasound irradiation; signal decays rapidly after ultrasound cessation	Limited intensity; dependent on energy-storage/release efficiency	More complex design and possible switching instability

**Table 2:** Organic initiators and substrates for sonoafterglow imaging.

Materials	Molecular structure	Mechanism	Ref.
Initiator			
RB	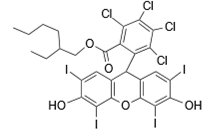	Iodine atoms enable near-unity intersystem crossing to the triplet state, promoting efficient Type II energy transfer to O₂ to produce ¹O₂.	Nat. Biomed. Eng 7, 298–312 (2023)
HMP	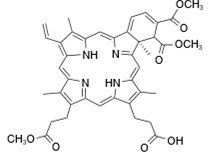	Ultrasound activates the porphyrin to its triplet state, which transfers an electron from the pyrrole nitrogen π-system to oxygen, generating singlet oxygen (¹O₂). Peripheral vinyl groups may also be oxidized.	Nat. Biomed. Eng 7, 298–312 (2023)
VP	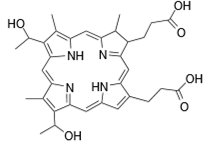	The reduced pyrrole ring increases electron density, enhancing electron donation from the porphyrin to oxygen for efficient ¹O₂ production.	Nat. Biomed. Eng 7, 298–312 (2023)
NCBS	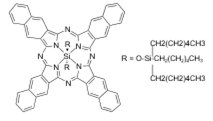	The expanded π-system and axial ligands of NCBS favor a long-lived triplet state, enabling efficient ¹O₂ generation *via* energy transfer. The silicon center and its ligands modulate the electronic properties for optimal sensitization.	Nat. Biomed. Eng 7, 298–312 (2023)
PFODBT	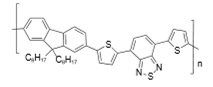	The piezoelectric effect causes band bending within the polymer nanoparticles, driving electrons and holes to the surface, where they reduce O₂ and oxidize H₂O/OH⁻, respectively to excite the π-conjugated backbone of the polymer.	Nat. Photon. 18, 334–343 (2024)
PFBT	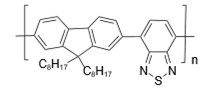		
MEHPPV	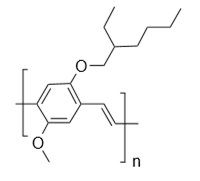		
TD	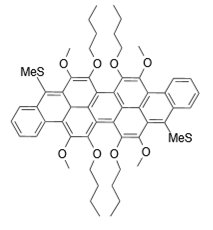	Ultrasound induces piezoelectric polarization generates separated electrons (e⁻) and holes (h⁺) that oxidize H₂O to hydroxyl radicals (^•^OH), which attack the central ring (meso-position) of the anthracene unit, leading to C-H bond cleavage, formation of carbonyl products, and consequent chemiluminescence.	Nat. Photon. 18, 334–343 (2024)Chem. Mater. 32, 5927-5936 (2020)ACS Appl. Mater. Interfaces 2024, 16, 56519-56544
Ce6	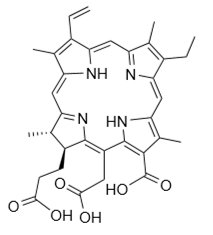	The porphyrin ring is excited by sonication and undergoes intersystem crossing to a long-lived triplet state (T₁), which generates singlet oxygen (¹O₂) *via* triplet-triplet annihilation with ground-state oxygen (³O₂). The central metal ion (or substituents like F in F-PpIX) fine-tunes this electron exchange process.	Nat. Photon. 18, 334–343 (2024)
F-PpIX	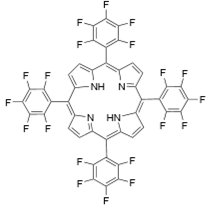		
BODIPY	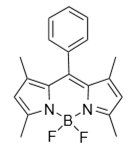	The bromine atom in BODIPY-Br enhances intersystem crossing to the triplet state *via* the heavy atom effect; subsequent electron transfer with oxygen or substrates produces radical species that excite the boron-chelated aza-indacene core.	Nat. Photon. 18, 334–343 (2024)Dyes Pigm. 160, 198-207 (2019)RSC Adv. 3, 9219-9222 (2013)Dyes Pigm. 112, 274-279 (2015)Biomol. Chem. 14, 7028-7037 (2016)
BODIPY-Br	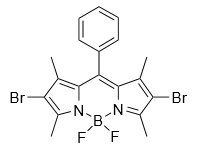		
Curcumin	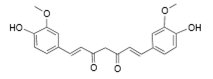	Ultrasound-generated radicals abstract hydrogen from its phenolic O-H group, triggering the central C-C bond oxidation and cleavage, and the formation of emissive fragments.	Nat. Photon. 18, 334–343 (2024)
ICG	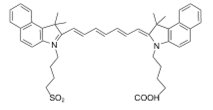	Reactive oxygen species (like ¹O₂) attack the polymethine chain (C=C)_n, cleaving the conjugated chain and producing excited-state carbonyl fragments (aldehydes/ketones).	Nat. Photon. 18, 334–343 (2024)
IR780	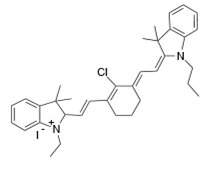		Nat. Photon. 18, 334–343 (2024)
HD	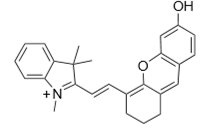		Nat. Photon. 18, 334–343 (2024)Chem. Soc. 134, 13510-13523 (2012).
BTz-IC-H	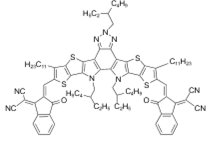	Strong intramolecular charge transfer (ICT) combined with piezoelectric polarization separates charges to drive surface redox reactions, populating the excited charge-transfer state.	Nat. Photon. 18, 334–343 (2024)ACS Appl. Mater. Interfaces 14, 18043-18052 (2022)Chem 6, 2147-2161 (2020)ACS Appl. Mater. Interfaces 11, 16311-16319 (2019)
BTz-IC-F	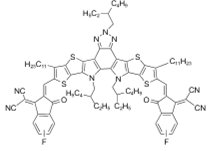		
BTz-IC-Cl	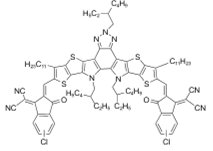		
Substrate			
PA	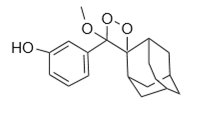	¹O₂ undergoes cycloaddition with the electron-rich C=C bond to form a high-energy 1,2-dioxetane, which thermally decomposes into an excited adamantanone.	Nat. Biomed. Eng 7, 298–312 (2023) ACS Cent. Sci., 3, 349-358 (2017)
AMPA	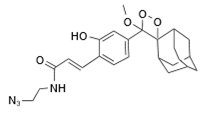	Primary dioxetane formation similar to PA; secondary homolysis of the azide (–N₃) group generates nitrene radicals that form additional emissive intermediates. Its core chemiluminescence mechanism is the same as PA (dioxetane formation). The unique azide group may provide a secondary pathway: under ultrasonic stress, the C-N₃ bond can homolyze to generate a nitrene radical, which can react to form other high-energy intermediates (*e.g.*, oxadiazoles) that also contribute to light emission.	Nat. Biomed. Eng 7, 298–312 (2023) Adv. Funct. Mater., 30, 2003628 (2020)
DPAo	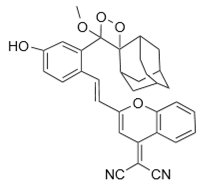	Similar to PA (¹O₂-triggered dioxetane), but electron-withdrawing groups stabilize intermediates and redshift emission *via* strong ICT effects.	Nat. Biomed. Eng 7, 298–312 (2023) J. Am. Chem. Soc., 139, 13243-13248 (2017)
DPAs	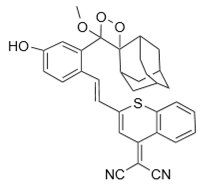		Nat. Biomed. Eng 7, 298–312 (2023) Angew. Chem. Int. Ed. Engl., 60, 3999-4003

Abbreviations: TD, trianthracene derivatives; PFODBT, poly[(2,6-(4,8-bis(5-(2-ethylhexyl)thiophen-2-yl)-benzo[1,2-b:4,5-b′]dithiophene))-alt-(5,5-(1′,3′-di-2-thienyl-5′,7′-bis(2-ethylhexyl)benzo[1′,2′-c:4′,5′-c′]dithiophene-4,8-dione))]; PFBT, poly(9,9-dioctylfluorenyl-2,7-diyl)-alt-(benzo[2,1,3]thiadiazol-4,8-diyl); MEHPPV, poly(2-methoxy-5-(2′-ethylhexyloxy)-1,4-phenylene vinylene); Ce6, chlorin e6; F-PpIX, foscan, or meta-tetra(hydroxyphenyl)chlorin; BODIPY, boron-dipyrromethene dye; BODIPY-Br, brominated boron-dipyrromethene dye; ICG, indocyanine green; IR780, IR-780 iodide; HD, heptamethine cyanine dye (specific structure of HD); BTz-IC-H, A-D-A′-D-A conjugated molecule with benzothiadiazole and indacenodithiophene cores (H-terminated); BTz-IC-F, A-D-A′-D-A conjugated molecule with benzothiadiazole and indacenodithiophene cores (F-terminated); BTz-IC-Cl, A-D-A′-D-A conjugated molecule with benzothiadiazole and indacenodithiophene cores (Cl-terminated); Curcumin, (1E,6E)-1,7-bis(4-hydroxy-3-methoxyphenyl)-1,6-heptadiene-3,5-dione; RB, rose bengal octyl ester; HMP, hematoporphyrin; VP, verteporfin; NCBS, silicon 2,3-naphthalocyanine bis(trihexylsilyloxide); PA, phenoxyl-adamantylidene; AMPA, azide-methyl acrylate-phenoxyl-adamantylidene; DPAo, dicyanomethylene-4H-benzopyran-phenoxyl-adamantylidene; DPAs, dicyanomethylene-4H-benzothiopyran-phenoxyl-adamantylidene.

**Table 3:** Representative biomarker-activatable Off-On probes in ultrasound-induced luminescence.

Biomarker (On-trigger)	Probes	Off-switch	Key chemical event	Ref.
	**Quenching**			
Granzyme B	TD-Grz-BHQ	TD donor linked to BHQ-3 quencher *via* a granzyme B-cleavable peptide (IEFD)	Proteolytic cleavage of the IEFD peptide by granzyme B, physically separating TD and BHQ-3	Nat. Photon. 18, 334–343 (2024).
Matrix metalloproteinase-2 (MMP-2)	TD-MMP-BHQ	TD donor linked to BHQ-3 quencher *via* an MMP-2-cleavable peptide (GPLGIA)	Proteolytic cleavage of the GPLGIA peptide by MMP-2	
Caspase-3	TD-caspase-BHQ	TD donor linked to BHQ-3 quencher *via* a caspase-3-cleavable peptide (DEVD)	Proteolytic cleavage of the DEVD peptide by caspase-3	
ONOO⁻	TD@IR780 NPs	IR780 dye (ONOO⁻-responsive) acts as an acceptor to quench the TD donor *via* RET	Oxidative degradation of the IR780 molecule by ONOO⁻, destroying its quenching ability	
	**Caging**			
	NCBS/DPAs SNAP (SNAP-M)	DPAs substrate caged by an ONOO⁻-responsive boronate ester (as Pro-DPAS)	Oxidative cleavage of the boronate ester cage by ONOO⁻, releasing the active DPAs	Nat. Biomed. Eng 7, 298–312 (2023).
	Pro-MB/Pro-R837/AMPA (SCAN)	1. Sonosensitizer MB caged as Pro-MB (*via* ONOO⁻-cleavable boronate). 2. Drug R837 caged as Pro-R837 (*via* ¹O₂-labile linker)	1. ONOO⁻ cleaves the boronate cage on Pro-MB, activating MB. 2. Ultrasound triggers activated MB to generate ¹O₂. 3. ¹O₂ cleaves the linker on Pro-R837.	
Hydrogen sulfide (H_2_S)	DPA-H_2_S	Luminogenic precursor caged by a H₂S-responsive 2,4-dinitrobenzene (DNB) ether.	Reductive cleavage of the DNB ether by H₂S, exposing an electron-rich double bond	Anal. Chem. 2024, 96, 37, 15031–15041
**Inactivity**				
MscL	Sono-bioluminescent nanoparticles (SNLPs)	The bioluminescent protein aequorin is inactive due to lack of Ca²⁺	Ultrasound mechanically gates the MscL ion channel, inducing Ca²⁺ influx	ACS Nano 2025, 19, 29, 26791–26804

Abbreviations: DPAs, Dicyanomethylene-4H-benzothiopyran-phenoxyl-adamantylidene; Pro-DPAs, ONOO⁻-caged DPAs (*via* boronate ester); NCBS, Silicon 2,3-naphthalocyanine bis(trihexylsilyloxide); MB, Methylene Blue; Pro-MB, ONOO⁻-caged Methylene Blue (*via* boronate ester); AMPA, Azide-methyl acrylate-phenoxyl-adamantylidene; Pro-R837, Singlet oxygen-caged imiquimod derivative; TD, Trianthracene; derivative nanoparticles; BHQ-3, Black Hole Quencher-3; IR780, IR-780 iodide (cyanine dye); MscL, Mechanosensitive channel of Large conductance; IEFD, Ile-Glu-Phe-Asp (peptide sequence for granzyme B); Ile-Glu-Phe-Asp; GPLGIA, Gly-Pro-Leu-Gly-Ile-Ala (peptide sequence for MMP-2); DEVD, Asp-Glu-Val-Asp (peptide sequence for caspase-3).

**Table 4:** Comparison analysis of diverse platforms for ultrasound-induced luminescence.

Platform	Representative systems	Structural definition	Batch consistency	Stability	Biodegradability	Clinical feasibility	Major strengths	Major limitations
Molecularly defined or near-molecular probes	PNCL, DPA-H_2_S, ncRuPA_APN_, PpIX-M	High	High	Moderate	Usually favorable to moderate	Moderate to relatively high	Clear composition, easier analytical characterization, more direct structure–property interpretation, simpler QC logic	Limited colloidal stability or solubility in some cases, shorter circulation, sometimes weaker acoustic amplification
Self-assembled organic sonoafterglow nanoparticles	NCBS/DPAs SNAP, NCBS/AMPA SNAP, Q SNAP, TD NPs, NPs-Ce4-SN, BTB-OC9/BDTSe, FZ970, TCL NPs, 3SCe-NP, FTA NPs	Moderate	Moderate	Moderate to good	Case-dependent	Moderate	Strong signal amplification, facile integration of sonosensitizer, afterglow substrate, quencher, and therapeutic cargo, activatable logic is readily built	Formulation-sensitive, packing state, cargo ratio, oxygen accessibility, and particle size may drift
Polymeric/semiconducting organic nanoplatforms	SPNs (SP1)@CoOOH, CSAPs, related semiconducting/polymer-containing hybrids	Moderate to low	Moderate to low	Good	Variable and polymer-dependent	Moderate to low	High modularity, convenient ROS amplification and theranostic integration	Greater compositional heterogeneity, more CQAs, harder scale-up and release specification
Liposomal carriers	Lipo@IR780/L012, Lipo@IR780/L012/CaO2	Moderate to high	Relatively high	Moderate to good	Usually favorable	Relatively high	Mature pharmaceutical logic, scalable lipid manufacturing, familiar regulatory framework, good biocompatibility	Leakage, oxidation, encapsulation efficiency, and storage stability remain constraints, especially for multi-cargo systems
Inorganic persistent-luminescent nanoplatforms	mSZ-Clr@ePDA NPs, Janus mSZ@PDA-NO NPs	High for core composition, moderate for surface-engineered systems	Moderate	High	Often less favorable	Low to moderate	Robust ultrasound rechargeability, persistent emission, strong mechanical stability	Slower degradation, possible long-term retention, and heavier translational burden
Hybrid/composite multifunctional systems	FURIN, FUSION, selected mSZ-based composites, selected activatable theranostic hybrids	Low to moderate	Low to moderate	Functionally robust	Depends on the least clearable component	Low to moderate	Highest functional integration, multimodal imaging/therapy, feedback-controlled design	Hardest to standardize, characterize, and scale, highest CMC and regulatory complexity

Abbreviations: QC, quality control; ROS, reactive oxygen species; CQA, critical quality attribute; CMC, chemistry, manufacturing, and controls. Note: This table classifies systems by their dominant translational determinant rather than a single absolute material label. Several reported ultrasound-excited luminescent systems are inherently cross-category hybrids, and the relative rankings are intended as qualitative trends rather than absolute rules.

**Table 5:** General molecular design strategies and structure–property relationships governing the four-stage energy-conversion cascade and luminescent and biological performance of ultrasound-induced luminescence systems.

Molecular engineering strategy	Changed structure–property relationship	Effect on energy pathway	Luminescent and biological outcome
Increased D–A strength	Reduced bandgap and enhanced charge-transfer character	Stage 2–3: facilitated excitation capture and charge, and energy conversion	Red-shifted emission, increased intensity and sonosensitization efficiency, improved imaging sensitivity and therapeutic response
Branched alkyl side chains	Suppressed π–π stacking and loosened intermolecular packing	Stage 4, with support to Stage 3: reduced aggregation-caused quenching and nonradiative decay	Brighter emission, prolonged afterglow lifetime, improved *in vivo* detectability
Heavy-atom substitution/ metal coordination	Enhanced spin–orbit coupling and intersystem crossing probability	Dominantly Stage 3, with support from Stage 2	Increased triplet-state utilization and ROS generation, enhanced afterglow/therapeutic efficacy
Artificial cavitation nuclei/ cavitation-active interfaces	Lowered cavitation threshold and intensified local acoustic/mechanical field	Stage 1: excitation input	Easier luminescence triggering, higher initial signal intensity, improved imaging sensitivity and ultrasound-activated therapy
Dynamic shell engineering for bubble stabilization	Prolonged lifetime of oscillating bubbles and sustained cavitation activity	Stage 1: excitation input	Stronger and more persistent sonoluminescence, longer imaging window, improved visualization performance
Piezoelectric host–dopant coupling	Tuned internal electric field, trap release efficiency, and emissive-center activation	Mainly Stage 2, partly Stage 3	Enhanced mechanoluminescence output, tunable visible/NIR emission, improved deep-tissue readout
Dopant engineering of emissive and trap centers	Modified defect distribution, transition energy, and emission center identity	Stage 2 and Stage 4	Tunable emission wavelength, adjustable luminescence color/intensity, potential for multiplexed imaging
Deep-trap and defect engineering	Increased density and stability of deep traps for carrier storage	Stage 2: energy capture/storage	Prolonged persistent luminescence/afterglow duration, stable NIR output, extended monitoring capability
Trap–acceptor spectral matching / co-doping	Coupled carrier detrapping with resonant donor-to-acceptor energy transfer	Stage 2–3	Enabled wavelength conversion to NIR-I/NIR-II, improved penetration depth, strengthened theranostic performance
Multi-pathway ROS amplification	Improved charge separation and integrated parallel ROS-generation routes	Stage 3: energy utilization and transformation	Increased chemical-energy pool, stronger luminescence output, enhanced sonodynamic/oxidative therapeutic efficacy
Local chemical microenvironment regulation of chemiluminescent substrates	Activated luminogens through pH/redox modulation and substrate deprotonation or uncaging	Stage 3	Higher chemiluminescence quantum yield, brighter activatable imaging, better therapy feedback
Chemical-energy reservoir formation	Generated metastable endoperoxide/dioxetane-like intermediates for delayed energy storage and release	Stage 3, with support to Stage 4	Prolonged afterglow duration, sustained post-ultrasound emission, increased cumulative therapeutic output
D–A modulation combined with anti-aggregation/ rigidification	Stabilized charge-separated states, narrowed ΔEST, reduced π–π stacking, and suppressed nonradiative relaxation	Stage 2–4, with dominant effect at Stage 3	Red-shifted and brighter afterglow, longer lifetime, higher SBR, improved efficacy with reduced long-term toxicity
Intramolecular short-range energy handoff	Minimized donor–emitter distance and reduced chemical-excitation transfer loss	Stage 3	More efficient NIR afterglow generation, enhanced brightness, improved deep-tissue imaging capability
Intermolecular spectral-overlap engineering (FRET/CRET)	Optimized donor-emission/acceptor-absorption matching within nanoassemblies	Stage 3	Controlled final emitter identity and wavelength, enhanced intensity/contrast, improved activatable imaging and therapy
Linker engineering/ controlled electronic decoupling	Balanced electronic communication with the stability of emissive intermediates	Stage 3–4	Reduced energy dissipation, stronger signal output, more persistent afterglow
Local energy-level perturbation/ microenvironment doping	Modified emitter energy landscape and radiative/nonradiative rate balance	Stage 4: emission competition	Markedly enhanced brightness and imaging sensitivity
Rigid confinement microenvironments	Restricted vibration/rotation, reduced oxygen quenching, and stabilized triplet excitons	Stage 4	Longer phosphorescence/afterglow duration, improved signal retention, prolonged tracking window
Ultrasound-promoted AIE assembly	Restricted intramolecular motion in the aggregated state	Stage 4, with partial support to Stage 3	Brighter emission, suppressed nonradiative loss, enhanced probe performance and light-harvesting efficiency
Rigid ICT skeleton and packing optimization	Strengthened ICT, facilitated ISC, and stabilized emissive packing/crystalline order	Stage 3–4	Red-shifted fluorescence/phosphorescence, increased SBR, improved subcutaneous or deep-tissue imaging
Through-space charge transfer/ cocrystallized packing	Enhanced 3D orbital coupling and rigidified lattice-level organization	Stage 3–4	Improved excited-state transformation, red-shifted and intensified emission, prolonged lifetime
Hybrid metal-halide/ hybrid lattice design	Tunable band structure, exciton behavior, and defect-assisted radiative pathways	Stage 2–4	Stronger optical output, wider spectral tunability, improved adaptability for imaging applications
Multi-cascade integrated afterglow design	Coordinated ROS generation, energy transfer, and suppression of nonradiative decay within one system	Stage 3–4	High-intensity and long-lasting afterglow, combined imaging guidance and synergistic therapy

## Abbreviations

AIE: aggregation-induced emission
FRET: Förster resonance energy transfer
FUS: focused ultrasound
ICT: intramolecular charge transfer
ISC: intersystem crossing
ML: mechanoluminescence
MRI: magnetic resonance imaging
NIR: near-infrared
NIR-I: first near-infrared window
NIR-II: second near-infrared window
NPs: nanoparticles
PEG: polyethylene glycol
PerL: persistent luminescence
RISC: reverse intersystem crossing
ROS: reactive oxygen species
SBR: signal-to-background ratio
SDT: sonodynamic therapy
TME: tumor microenvironment
US: ultrasound

## References

[1] Yang Y, Jiang Q, Zhang F (2024). Nanocrystals for Deep-Tissue In Vivo Luminescence Imaging in the Near-Infrared Region. Chemical Reviews.

[2] Liu Y, Valji K, Monsky W, Zheng C, Yang X (2025). Optical imaging guidance in oncologic surgery and interventional oncology. Pharmacological Research.

[3] Jiang Y, Pu K (2021). Molecular Probes for Autofluorescence-Free Optical Imaging. Chemical Reviews.

[4] Klinkhammer BM, Lammers T, Mottaghy FM, Kiessling F, Floege J, Boor P (2021). Non-invasive molecular imaging of kidney diseases. Nature reviews Nephrology.

[5] Jokerst JV, Gambhir SS (2011). Molecular imaging with theranostic nanoparticles. Accounts of chemical research.

[6] Diao S, Blackburn JL, Hong G, Antaris AL, Chang J, Wu JZ (2015). Fluorescence Imaging In Vivo at Wavelengths beyond 1500 nm. Angewandte Chemie (International ed in English).

[7] Yang M, Huang J, Fan J, Du J, Pu K, Peng X (2020). Chemiluminescence for bioimaging and therapeutics: recent advances and challenges. Chemical Society reviews.

[8] Lin Y, Huang J, Pu K (2025). Near-Infrared Chemiluminescent Theranostics. Angewandte Chemie International Edition.

[9] Xia Y, Zhang R, Wang Z, Tian J, Chen X (2017). Recent advances in high-performance fluorescent and bioluminescent RNA imaging probes. Chemical Society reviews.

[10] Shaffer TM, Pratt EC, Grimm J (2017). Utilizing the power of Cerenkov light with nanotechnology. Nature nanotechnology.

[11] Pei P, Chen Y, Sun C, Fan Y, Yang Y, Liu X (2021). X-ray-activated persistent luminescence nanomaterials for NIR-II imaging. Nature nanotechnology.

[12] Wei Y, Wang J (2024). X-ray/γ-ray/Ultrasound-Activated Persistent Luminescence Phosphors for Deep Tissue Bioimaging and Therapy. ACS applied materials & interfaces.

[13] Díaz-Gómez JL, Mayo PH, Koenig SJ (2021). Point-of-Care Ultrasonography. The New England journal of medicine.

[14] Zhang G, Ye HR, Sun Y, Guo ZZ (2022). Ultrasound Molecular Imaging and Its Applications in Cancer Diagnosis and Therapy. ACS sensors.

[15] Whitman GJ, Hortobagyi GN (2017). Ultrasound Molecular Imaging: A Good Start. Journal of clinical oncology: official journal of the American Society of Clinical Oncology.

[16] Farhadi A, Ho GH, Sawyer DP, Bourdeau RW, Shapiro MG (2019). Ultrasound imaging of gene expression in mammalian cells. Science (New York, NY).

[17] Wang Y, Yi Z, Guo J, Liao S, Li Z, Xu S (2024). In vivo ultrasound-induced luminescence molecular imaging. Nature Photonics.

[18] Frenzel H, Schultes H (1934). Luminescenz im ultraschallbeschickten Wasser. Zeitschrift für Physikalische Chemie.

[19] Doktycz SJ, Suslick KS (1990). Interparticle collisions driven by ultrasound. Science (New York, NY).

[20] Didenko YT, McNamara WB 3rd, Suslick KS (2000). Molecular emission from single-bubble sonoluminescence. Nature.

[21] Jarman P (1960). Sonoluminescence: A Discussion. The Journal of the Acoustical Society of America.

[22] Barber BP, Putterman SJ (1991). Observation of synchronous picosecond sonoluminescence. Nature.

[23] Gaitan DF, Crum LA, Church CC, Roy RA (1992). Sonoluminescence and bubble dynamics for a single, stable, cavitation bubble. The Journal of the Acoustical Society of America.

[24] Yonghong H, Da X, Shici T, Yonghong T, Ken-ichi U (2002). In vivo sonoluminescence imaging with the assistance of FCLA. Physics in Medicine & Biology.

[25] He Y, Xing D, Yan G, Ueda K-i (2002). FCLA chemiluminescence from sonodynamic action in vitro and in vivo. Cancer Letters.

[26] He Y, Xing D, Wang J (2003). A novel cancer imaging method using chemiluminescence-mediated sonosensitization. Chinese Science Bulletin.

[27] Ohl S-W, Ow DSW, Klaseboer E, Wong VV, Dumke R, Ohl C-D (2011). Sonochemistry and sonoluminescence in microfluidics. Proceedings of the National Academy of Sciences.

[28] Fernandez Rivas D, Ashokkumar M, Leong T, Yasui K, Tuziuti T, Kentish S (2012). Sonoluminescence and sonochemiluminescence from a microreactor. Ultrasonics sonochemistry.

[29] Terasaki N, Yamada H, Xu C-N (2013). Ultrasonic wave induced mechanoluminescence and its application for photocatalysis as ubiquitous light source. Catalysis Today.

[30] Beguin E, Shrivastava S, Dezhkunov NV, McHale AP, Callan JF, Stride E (2019). Direct Evidence of Multibubble Sonoluminescence Using Therapeutic Ultrasound and Microbubbles. ACS applied materials & interfaces.

[31] Wu X, Zhu X, Chong P, Liu J, Andre LN, Ong KS (2019). Sono-optogenetics facilitated by a circulation-delivered rechargeable light source for minimally invasive optogenetics. Proceedings of the National Academy of Sciences.

[32] Wang W, Kevin Tang KW, Pyatnitskiy I, Liu X, Shi X, Huo D (2023). Ultrasound-Induced Cascade Amplification in a Mechanoluminescent Nanotransducer for Enhanced Sono-Optogenetic Deep Brain Stimulation. ACS nano.

[33] Ding Y, So B, Cao J, Wondraczek L (2022). Ultrasound-Induced Mechanoluminescence and Optical Thermometry Toward Stimulus-Responsive Materials with Simultaneous Trigger Response and Read-Out Functions. Advanced science (Weinheim, Baden-Wurttemberg, Germany).

[34] Xu C, Huang J, Jiang Y, He S, Zhang C, Pu K (2023). Nanoparticles with ultrasound-induced afterglow luminescence for tumour-specific theranostics. Nature biomedical engineering.

[35] Zhang Z, Yan H, Cao W, Xie S, Ran P, Wei K (2023). Ultrasound-Chargeable Persistent Luminescence Nanoparticles to Generate Self-Propelled Motion and Photothermal/NO Therapy for Synergistic Tumor Treatment. ACS nano.

[36] Zhou D, Zhang Z, Qiu B, Zhang D, Xie S, Huang K (2022). Ultrasound-Activated Persistent Luminescence Imaging and Bacteria-Triggered Drug Release for Helicobacter pylori Infection Theranostics. ACS applied materials & interfaces.

[37] Chang K, Gu J, Yuan L, Guo J, Wu X, Fan Y (2024). Achieving Ultrasound-Excited Emission with Organic Mechanoluminescent Materials. Advanced materials (Deerfield Beach, Fla).

[38] Xu X, Wang Y, Li Z, Zhang X-B, Song G (2026). In vivo ultrasound-induced luminescence imaging via trianthracene derivatives nanomaterials. Nature Protocols.

[39] Li X, Tan Y, Li Z, Wang Y, Lu C, Liao S (2025). Cascade Energy Transformation in Hybrid Nanosensitizer Enables Ultrasound-Activated Luminescence Imaging and Enhanced Sonodynamic Therapy. Journal of the American Chemical Society.

[40] Jiang S, Malinao MG, Yang F, Zeng Y, Hou SS, Wu X (2026). An ultrasound-scanning in vivo light source. Nature Materials.

[41] Li Z, Xiang J, Wu X, Cai J, Lv F, Yin B (2026). Long-Acting Fractionated Sonodynamic Therapy Enabled by Fused-Trianthracene Nanoparticles with Ultra-Bright Ultrasound-Induced Afterglow Luminescence. Journal of the American Chemical Society.

[42] Fan D, Liu M, Zhou Y, Huang X, Cheng X, Huang S (2026). Smart Sonoafterglow Nanobombs for High-Contrast Imaging and Synergistic Gas-Sonodynamic Therapy of Hypoxic Tumors. Angewandte Chemie International Edition.

[43] Wang Q, Tian Y, Yao M, Fu J, Wang L, Zhu Y (2023). Bimetallic Organic Frameworks of High Piezovoltage for Sono-Piezo Dynamic Therapy. Advanced Materials.

[44] Qu R, Jiang X, Zhen X (2024). Light/X-ray/ultrasound activated delayed photon emission of organic molecular probes for optical imaging: mechanisms, design strategies, and biomedical applications. Chemical Society reviews.

[45] Wu J, Zhou X, Tsang CY, Mei Q, Zhang Y (2025). Bioengineered nanomaterials for dynamic diagnostics in vivo. Chemical Society reviews.

[46] Wang Y, Xu Y, Dong S, Wang P, Chen W, Lu Z (2021). Ultrasonic activation of inert poly(tetrafluoroethylene) enables piezocatalytic generation of reactive oxygen species. Nature communications.

[47] Wu R, Yao Z, Chen Z, Ge X, Su L, Wang S (2023). Ultrasound-Activated NIR Chemiluminescence for Deep Tissue and Tumor Foci Imaging. Analytical chemistry.

[48] Canaparo R, Foglietta F, Giuntini F, Francovich A, Serpe L (2020). The bright side of sound: perspectives on the biomedical application of sonoluminescence. Photochemical & photobiological sciences: Official journal of the European Photochemistry Association and the European Society for Photobiology.

[49] Suslick KS, Doktycz SJ, Flint EB (1990). On the origin of sonoluminescence and sonochemistry. Ultrasonics.

[50] Ashokkumar M, Lee J, Kentish S, Grieser F (2007). Bubbles in an acoustic field: An overview. Ultrasonics sonochemistry.

[51] Kanthale P, Ashokkumar M, Grieser F (2008). Sonoluminescence, sonochemistry (H2O2 yield) and bubble dynamics: frequency and power effects. Ultrasonics sonochemistry.

[52] Rooze J, Rebrov EV, Schouten JC, Keurentjes JT (2013). Dissolved gas and ultrasonic cavitation-a review. Ultrasonics sonochemistry.

[53] Sazgarnia A, Shanei A, Eshghi H, Hassanzadeh-Khayyat M, Esmaily H, Shanei MM (2013). Detection of sonoluminescence signals in a gel phantom in the presence of Protoporphyrin IX conjugated to gold nanoparticles. Ultrasonics.

[54] Li C, Luo Z, Shao Y, Qian Y, Du S, Yang Q (2025). A review on ultrasound-enhanced heat transfer. Ultrasonics sonochemistry.

[55] Chen X, Wu D, Chen Z (2024). Biomedical applications of stimuli-responsive nanomaterials. MedComm.

[56] Dezhkunov NV (2002). Multibubble sonoluminescence intensity dependence on liquid temperature at different ultrasound intensities. Ultrasonics sonochemistry.

[57] Wang X, Dai X, Chen Y (2023). Sonopiezoelectric Nanomedicine and Materdicine. Small (Weinheim an der Bergstrasse, Germany).

[58] Cafarelli A, Marino A, Vannozzi L, Puigmartí-Luis J, Pané S, Ciofani G (2021). Piezoelectric Nanomaterials Activated by Ultrasound: The Pathway from Discovery to Future Clinical Adoption. ACS nano.

[59] Yang S, Wang Y, Liang X (2023). Piezoelectric Nanomaterials Activated by Ultrasound in Disease Treatment. Pharmaceutics.

[60] Dalecki D (2004). Mechanical Bioeffects of Ultrasound. Annual Review of Biomedical Engineering.

[61] Stratmeyer ME, Greenleaf JF, Dalecki D, Salvesen KA (2008). Fetal ultrasound: mechanical effects. Journal of ultrasound in medicine: official journal of the American Institute of Ultrasound in Medicine.

[62] Thanh Nguyen T, Asakura Y, Koda S, Yasuda K (2017). Dependence of cavitation, chemical effect, and mechanical effect thresholds on ultrasonic frequency. Ultrasonics sonochemistry.

[63] O'Reilly MA (2024). Exploiting the mechanical effects of ultrasound for noninvasive therapy. Science (New York, NY).

[64] Xu S, Li X, Hu Q, Zhang J, Li R, Meng L (2024). Focused Ultrasound-Responsive Nanocomposite with Near-Infrared II Mechanoluminescence for Spatiotemporally Selective Immune Activation in Lymph Nodes. Chemistry.

[65] Tu L, Xie Y, Li Z (2022). Advances in Pure Organic Mechanoluminescence Materials. The Journal of Physical Chemistry Letters.

[66] Eng J, Penfold TJ (2021). Open questions on the photophysics of thermally activated delayed fluorescence. Communications Chemistry.

[67] Shi H, Yao W, Ye W, Ma H, Huang W, An Z (2022). Ultralong Organic Phosphorescence: From Material Design to Applications. Accounts of chemical research.

[68] Xu C, Zhang Y, Liang G, Pu K (2025). Molecular afterglow imaging for biomedical applications. Nature Materials.

[69] Sun Y, Wu L, Zhu L, Baryshnikov GV, Zhang F, Li X (2025). Recent Advances in Thermally Activated Delayed Fluorescence-Based Organic Afterglow Materials. Small methods.

[70] Li Z, Liu H, Zhang XB (2024). Reactive oxygen species-mediated organic long-persistent luminophores light up biomedicine: from two-component separated nano-systems to integrated uni-luminophores. Chemical Society reviews.

[71] Yao Z, Xu F, Wu R, Wang X, Guo M, Wang S (2024). Dual-Locked Probe with Activatable Sonoafterglow Luminescence for Precise Imaging of MET-Induced Liver Injury. Analytical chemistry.

[72] Xu C, He S, Wei X, Huang J, Xu M, Pu K (2023). Activatable Sonoafterglow Nanoprobes for T-Cell Imaging. Advanced materials (Deerfield Beach, Fla).

[73] Kunti AK, Banerjee D (2023). 3 - Lanthanide-doped phosphor: an overview. In: Pawade VB, Kohale RL, Dhoble SJ, Swart HC, editors. Phosphor Handbook: Woodhead Publishing.

[74] Zheng B, Fan J, Chen B, Qin X, Wang J, Wang F (2022). Rare-Earth Doping in Nanostructured Inorganic Materials. Chemical Reviews.

[75] Li H-H, Wang Y-K, Liao L-S (2024). Near-Infrared Luminescent Materials Incorporating Rare Earth/Transition Metal Ions: From Materials to Applications. Advanced Materials.

[76] Patel DK, Sharma VK (2025). Illuminating Innovations: A Comprehensive Review of Rare Earth-Doped Phosphors and Their Applications in LEDs. Transactions of the Indian Institute of Metals.

[77] Voncken JHL (2016). Physical and Chemical Properties of the Rare Earths. In: Voncken JHL, editor. The Rare Earth Elements: An Introduction. Cham: Springer International Publishing.

[78] Shen J, Sun L-D, Yan C-H (2008). Luminescent rare earth nanomaterials for bioprobe applications. Dalton Transactions.

[79] Lee LC-C, Lo KK-W (2024). Shining New Light on Biological Systems: Luminescent Transition Metal Complexes for Bioimaging and Biosensing Applications. Chemical Reviews.

[80] Parayil RT, Gupta SK, Mohapatra M (2025). A review on defect engineered NIR persistent luminescence through transition metal ion (Cr, Mn, Fe and Ni) doping: Wider perspective covering synthesis, characterization, fundamentals and applications. Coordination Chemistry Reviews.

[81] Kiran R, Kamath N, Sayyed MI, Almuqrin AH, Kamath SD (2025). A review of recent developments in rare earth-doped nanophosphors for emerging technological applications. RSC Advances.

[82] Yang F, Kim S-J, Wu X, Cui H, Hahn SK, Hong G (2023). Principles and applications of sono-optogenetics. Advanced drug delivery reviews.

[83] Sweeting LM, Rheingold AL (1988). Crystal structure and triboluminescence. 1. 9-Anthryl carbinols. The Journal of Physical Chemistry.

[84] Hardy GE, Baldwin JC, Zink JI, Kaska WC, Liu P-H, Dubois L (1977). Triboluminescence spectroscopy of aromatic compounds. Journal of the American Chemical Society.

[85] Sweeting LM, Cashel ML, Dott M, Gingerich JM, Guido JL, Kling JA (1992). Spectroscopy and Mechanism in Triboluminescence. Molecular Crystals and Liquid Crystals Science and Technology Section A Molecular Crystals and Liquid Crystals.

[86] Nishida J, Ohura H, Kita Y, Hasegawa H, Kawase T, Takada N (2016). Phthalimide Compounds Containing a Trifluoromethylphenyl Group and Electron-Donating Aryl Groups: Color-Tuning and Enhancement of Triboluminescence. The Journal of organic chemistry.

[87] Wang Z, Chu C, Hao A, Xing P (2025). Dynamic Borate Esterification for Evolved Supramolecular Chirality and Chiral Optics. Angewandte Chemie International Edition.

[88] Wu Y, Xia Y, Yuan Y, Dou Z, Kan C, Wang T (2024). Mechanochromism of π-conjugated borate derivatives and the effect of intermolecular non-covalent forces on molecular packing and mechanochromic property. Journal of Molecular Structure.

[89] Xu B, He J, Mu Y, Zhu Q, Wu S, Wang Y (2015). Very bright mechanoluminescence and remarkable mechanochromism using a tetraphenylethene derivative with aggregation-induced emission. Chemical science.

[90] Nakayama H, Nishida J-i, Takada N, Sato H, Yamashita Y (2012). Crystal Structures and Triboluminescence Based on Trifluoromethyl and Pentafluorosulfanyl Substituted Asymmetric N-Phenyl Imide Compounds. Chemistry of Materials.

[91] Xie Z, Yu T, Chen J, Ubba E, Wang L, Mao Z (2018). Weak interactions but potent effect: tunable mechanoluminescence by adjusting intermolecular C-H···π interactions. Chemical science.

[92] Kim G, Lau VM, Halmes AJ, Oelze ML, Moore JS, Li KC (2019). High-intensity focused ultrasound-induced mechanochemical transduction in synthetic elastomers. Proceedings of the National Academy of Sciences of the United States of America.

[93] Li Y, Clough JM (2025). Optical Force Monitoring in Polymeric Materials with a Coumarin-Based Mechanophore. Angewandte Chemie International Edition.

[94] Wang C, Yang X, Duan R, Li X, Liu Y, Liu W (2024). Piezochromic luminescence of excimers formed by anthracene plane slip. Dyes and Pigments.

[95] Xu S, Liu T, Mu Y, Wang Y-F, Chi Z, Lo C-C (2015). An Organic Molecule with Asymmetric Structure Exhibiting Aggregation-Induced Emission, Delayed Fluorescence, and Mechanoluminescence. Angewandte Chemie International Edition.

[96] Wang W, Wu X, Kevin Tang KW, Pyatnitskiy I, Taniguchi R, Lin P (2023). Ultrasound-Triggered In Situ Photon Emission for Noninvasive Optogenetics. Journal of the American Chemical Society.

[97] Liang Y, Shao H, Liu K, Cao Q, Zhang S, Wang H (2025). Ultrasound-responsive phosphorescence in aqueous solution enabled by microscale rigid framework engineering of carbon nanodots. Light, science & applications.

[98] Datta P, Moolayadukkam S, Prasad Sahu R, Ganguly R, Sen S, Puri IK (2024). Deciphering the hydrodynamics of lipid-coated microbubble sonoluminescence for sonodynamic therapy. Ultrasonics sonochemistry.

[99] Pan X, Zhuang Y, He W, Lin C, Mei L, Chen C (2024). Quantifying the interfacial triboelectricity in inorganic-organic composite mechanoluminescent materials. Nature communications.

[100] Rejab MRBM, Hamdan MHBM, Quanjin M, Siregar JP, Bachtiar D, Muchlis Y (2020). Historical Development of Hybrid Materials. In: Hashmi S, Choudhury IA, editors. Encyclopedia of Renewable and Sustainable Materials. Oxford: Elsevier.

[101] Xu S, Meng L, Hu Q, Li F, Zhang J, Kong N (2024). Closed-Loop Control of Macrophage Engineering Enabled by Focused-Ultrasound Responsive Mechanoluminescence Nanoplatform for Precise Cancer Immunotherapy. Small (Weinheim an der Bergstrasse, Germany).

[102] Vighetto V, Pascucci E, Percivalle NM, Troia A, Meiburger KM, Broek MRPvd (2025). Functional nanocrystal as effective contrast agents for dual-mode imaging: Live-cell sonoluminescence and contrast-enhanced echography. Ultrasonics sonochemistry.

[103] Gao R, Kodaimati MS, Yan D (2021). Recent advances in persistent luminescence based on molecular hybrid materials. Chemical Society reviews.

[104] Ding Y, So B, Cao J, Langenhorst F, Wondraczek L (2023). Light Delivery, Acoustic Read-Out, and Optical Thermometry Using Ultrasound-Induced Mechanoluminescence and the Near-Infrared Persistent Luminescence of CaZnOS:Nd3+. Advanced Optical Materials.

[105] Cao J, Ding Y, Sun M, Sajzew R, Langenhorst F, Wondraczek L (2023). Ultrasound-Induced Luminescence from Cr3+-Doped ZnGa2O4 Glass-Ceramic Composites. Advanced Photonics Research.

[106] Wang J, Zou H, Qin J, Zhang M, Zhu K, Zhang B (2026). Spatiotemporally Controlled NIR-II Afterglow Nanoprobes via Synergistic Ultrasound and Peroxynitrite-Induced Energy Transfer for Precision Theranostics. Journal of the American Chemical Society.

[107] Yuan M, Fang X, Liu W, Ge X, Wu Y, Su L (2025). Ultrasound-Activated Near-Infrared-II Afterglow Luminescence for Precise Cancer Imaging. ACS applied bio materials.

[108] Wang Y, Yin X-B (2025). Persistent luminescence materials for imaging and therapeutic applications. Coordination Chemistry Reviews.

[109] Pan W, Rafiq M, Haider W, Guo Y, Wang H, Xu M (2024). Recent advances in NIR-II fluorescence/photoacoustic dual-modality imaging probes. Coordination Chemistry Reviews.

[110] Zhang L, Zhang L, Zhang X, Zhao Y, Fang S, You J (2023). Responsive fluorescent probes for cellular microenvironment and redox small biomolecules. TrAC Trends in Analytical Chemistry.

[111] Wang L, Liu Y, Sun J, Su J, Feng J, Miao L (2025). In Situ Ultrasound-Triggered Bioluminescence for Combined Sono/Photodynamic Immunotherapy. ACS nano.

[112] Wang Z, Jin L, Ni H, Kim H, Zeng Y, Zhou Q (2025). Controllable Doping for Tunable and Multimodal Emission in ZnS-Based Mechanoluminescent Nanocrystals. Nano letters.

[113] Drozdowski A, Soler-Carracedo K, Barzowska J, Mahlik S, Grzyb T, Runowski M (2026). Huge Enhancement of Green Persistent Mechanoluminescence of SrAl(2)O(4):Dy(3+)-Eu(2+) by Nd(3+) for Sound-to-Light Conversion, Handwriting Visualization, Force Sensing, and Luminescence Thermometry. Small (Weinheim an der Bergstrasse, Germany).

[114] Xiang C, Li C, Liu Y, Yang X, Xiang J, Li C (2026). A biodegradable theranostic sonosensitizer for afterglow-guided sonodynamic therapy. Acta biomaterialia.

[115] Wang T, Huang J, Xu C, Zhang L, Lin Y, Pu K (2026). Ruthenium-Based Sonoafterglow Probes for Tumor-Specific Theranostics. Angewandte Chemie International Edition.

[116] Tu Y, Zhao Z, Lam JWY, Tang BZ (2021). Mechanistic connotations of restriction of intramolecular motions (RIM). National Science Review.

[117] Tian Y, Huang W, Sheng Z, Yan D, Wang D, Tang BZ (2025). Nonlight-Driven Aggregation-Induced Emission Luminogens for Bioimaging and Theranostics. Chemical & Biomedical Imaging.

[118] Zeng W, Xu Y, Yang W, Liu K, Bian K, Zhang B (2020). An Ultrasound-Excitable Aggregation-Induced Emission Dye for Enhanced Sonodynamic Therapy of Tumors. Advanced Healthcare Materials.

[119] Du Z, Chen W, Tian M, Liu J, Kang X, Qi J (2025). Aggregation-induced emission sonosensitizers: molecular engineering and biomedical applications. Chemical Communications.

[120] Li C, Yan J, Yin B, Zhang Q, Huang Y, Chen J (2024). Scaffold-Free Efficient Light-Harvesting Nanoparticles Based on One-Pot Self-Assembly of Donor-Acceptor Aggregation-Induced Emission Luminogens. ACS Applied Nano Materials.

[121] Zhu F-Y, Mei L-J, Tian R, Li C, Wang Y-L, Xiang S-L (2024). Recent advances in super-resolution optical imaging based on aggregation-induced emission. Chemical Society reviews.

[122] Xu Z, Zeng Y, Kang M, Zhang Z, Yang Z, Fan M (2026). Dual-Enhanced Sonodynamic Therapy: Synergizing Cavitation Amplification and Efficient Sonoluminescence-to-ROS Conversion for Orthotopic Breast Cancer Treatment. Advanced Materials.

[123] McCarron J, Turner B, McHugh LN (2025). Hybrid Framework Materials: Next-Generation Engineering Materials. Advanced Engineering Materials.

[124] Li W, Liang Z, Wang P, Ma Q (2024). The luminescent principle and sensing mechanism of metal-organic framework for bioanalysis and bioimaging. Biosensors & bioelectronics.

[125] Xiao Y, Shen M, Chan C-Y, Yu T, Huang W (2025). Ultralong organic afterglow from small molecular host-guest materials: state of the art. Light: Science & Applications.

[126] Li X, Yang Y, Zhang R, Huang W (2025). Construction and optimization of organic fluorophores in NIR-II fluorescence imaging. Chemical Society reviews.

[127] Tian R, Zeng Q, Zhu S, Lau J, Chandra S, Ertsey R (2019). Albumin-chaperoned cyanine dye yields superbright NIR-II fluorophore with enhanced pharmacokinetics. Science Advances.

[128] Yang S-Y, Feng Z-Q, Liu C, Zhang J, Deng Z, Zhang K (2026). Heptacyclic spiro-TADF emitters with efficient long-wavelength emission via through-space interactions. Science China Chemistry.

[129] Yin Z, Xie Z, Zhang X, Xue Y, Zhang D, Liu B (2025). Cocrystallization-Induced Red Ultralong Organic Phosphorescence. Angewandte Chemie (International ed in English).

[130] Zhao L, Miao Q (2025). Organic afterglow luminescence for disease diagnosis and treatment. Nature Reviews Bioengineering.

[131] Zhang X, Jiang S, Xu Z-T, Pang A, Shi C-M (2025). Strategy for tuning luminescence properties of metal halide hybrid materials: ion regulation. CrystEngComm.

[132] Liang S, Hu D, Li G, Gao D, Li F, Zheng H (2022). NIR-II fluorescence visualization of ultrasound-induced blood-brain barrier opening for enhanced photothermal therapy against glioblastoma using indocyanine green microbubbles. Science bulletin.

[133] Park J, Oh D, Yoo J, Ha H, Kim D, Kim HH (2025). Clinical ultrasound, photoacoustic, and fluorescence image-guided lymphovenous anastomosis microsurgery via a transparent ultrasound transducer array. Nature communications.

[134] Zhang Y, Wen J, Fan X, Yu Y, Zhang C, Sun Y (2025). Three-Dimensional Afterglow Luminescence-MRI Fusion Tomography with Magnetically Targeted Luminescent-Magnetic Nanoparticles for Deep-Seated Tumor Imaging. ACS nano.

[135] Xu C, Huang J, Pu K (2025). Sonoafterglow nanoprobes for deep-tissue imaging of peroxynitrite. Nat Protoc.

[136] Mahmoudi M, Landry MP, Moore A, Coreas R (2023). The protein corona from nanomedicine to environmental science. Nature Reviews Materials.

[137] Ju Y, Kent SJ (2025). Balancing stealth and targeting to improve nanomedicine efficacy. Nature nanotechnology.

[138] Thomas OS, Weber W (2019). Overcoming Physiological Barriers to Nanoparticle Delivery-Are We There Yet?. Frontiers in bioengineering and biotechnology.

[139] Jiang Y, Zhang J, Ye J, Jin X, Zhao Y, Xu H (2025). Intratumoral Bacterial-Targeted Cascade-Responsive Sonoafterglow Nanoprobes for Immune Reprogramming and Visualization in Glioblastoma. Analytical chemistry.

[140] Bajracharya R, Song JG, Patil BR, Lee SH, Noh HM, Kim DH (2022). Functional ligands for improving anticancer drug therapy: current status and applications to drug delivery systems. Drug delivery.

[141] Yan S, Na J, Liu X, Wu P (2024). Different Targeting Ligands-Mediated Drug Delivery Systems for Tumor Therapy. Pharmaceutics.

[142] Sun X, Guo L, Shang M, Shi D, Liang P, Jing X (2020). Ultrasound Mediated Destruction of LMW-HA-Loaded and Folate-Conjugated Nanobubble for TAM Targeting and Reeducation. International journal of nanomedicine.

[143] Zhang H, Zhuang Y, Song J, Shao F, Shi W (2026). Biomimetic Nanoplatform Based on Macrophage Membrane-Coated Fe3O4 Nanoparticles for Synergistic Ferroptosis and Sonodynamic Therapy of Lung Cancer. International journal of nanomedicine.

[144] Wilhelm S, Tavares AJ, Dai Q, Ohta S, Audet J, Dvorak HF (2016). Analysis of nanoparticle delivery to tumours. Nature Reviews Materials.

[145] Dasgupta A, Sofias AM, Kiessling F, Lammers T (2024). Nanoparticle delivery to tumours: from EPR and ATR mechanisms to clinical impact. Nature Reviews Bioengineering.

[146] Duan L, Yang L, Jin J, Yang F, Liu D, Hu K (2020). Micro/nano-bubble-assisted ultrasound to enhance the EPR effect and potential theranostic applications. Theranostics.

[147] Liu D, Ling Y, Liu W, Deng J, Guo Y (2025). Ultrasound-responsive micro/nanobubbles based intelligent theranostic systems for precision tumor therapy. Journal of Nanobiotechnology.

[148] Yu M, Xu J, Zheng J (2019). Renal Clearable Luminescent Gold Nanoparticles: From the Bench to the Clinic. Angewandte Chemie (International ed in English).

[149] Cheng P, Pu K (2021). Molecular imaging and disease theranostics with renal-clearable optical agents. Nature Reviews Materials.

[150] Yang Z, Shen X, Jin J, Jiang X, Pan W, Wu C (2024). Sonosynthetic Cyanobacteria Oxygenation for Self-Enhanced Tumor-Specific Treatment. Advanced Science.

[151] Meng L, Xu S, Hu Q, Wang H, Wang P, Li R (2025). Mild Focused Ultrasound-Induced Microscopic Heating of Nanoparticles Observed by Lanthanide Luminescence for Precise Sonothermal Cancer Therapy. Nano letters.

[152] Hao Y, Liu Y, Liu X, Ma S, Wang C, Miao Q (2026). Deep excitation afterglow luminescent probes for biomedical applications. Chemical science.

[153] Wang W, Tasset A, Pyatnitskiy I, Mohamed HG, Taniguchi R, Zhou R (2022). Ultrasound triggered organic mechanoluminescence materials. Advanced drug delivery reviews.

[154] Jacques SL (2013). Optical properties of biological tissues: a review. Physics in medicine and biology.

[155] Wells PN, Liang HD (2011). Medical ultrasound: imaging of soft tissue strain and elasticity. Journal of the Royal Society, Interface.

[156] Kyriakides TR, Raj A, Tseng TH, Xiao H, Nguyen R, Mohammed FS (2021). Biocompatibility of nanomaterials and their immunological properties. Biomedical materials (Bristol, England).

[157] Choi HS, Liu W, Misra P, Tanaka E, Zimmer JP, Itty Ipe B (2007). Renal clearance of quantum dots. Nature biotechnology.

[158] Panico S, Capolla S, Bozzer S, Toffoli G, Dal Bo M, Macor P (2022). Biological Features of Nanoparticles: Protein Corona Formation and Interaction with the Immune System. Pharmaceutics.

[159] Abu Lila AS, Kiwada H, Ishida T (2013). The accelerated blood clearance (ABC) phenomenon: clinical challenge and approaches to manage. Journal of controlled release: official journal of the Controlled Release Society.

[160] Mima Y, Hashimoto Y, Shimizu T, Kiwada H, Ishida T (2015). Anti-PEG IgM Is a Major Contributor to the Accelerated Blood Clearance of Polyethylene Glycol-Conjugated Protein. Molecular pharmaceutics.

[161] Paliwal R, Babu RJ, Palakurthi S (2014). Nanomedicine scale-up technologies: feasibilities and challenges. AAPS PharmSciTech.

[162] Martin E, Aubry J-F, Schafer M, Verhagen L, Treeby B, Pauly KB (2024). ITRUSST consensus on standardised reporting for transcranial ultrasound stimulation. Brain Stimulation: Basic, Translational, and Clinical Research in Neuromodulation.

[163] Karthikesh MS, Yang X (2021). The effect of ultrasound cavitation on endothelial cells. Experimental biology and medicine (Maywood, NJ).

[164] Zhu Y, Fang J, Ahmed SAH, Zhang T, Zeng S, Liao J-Y (2025). A modular artificial intelligence framework to facilitate fluorophore design. Nature communications.

[165] Dong J, Qian J, Yu K, Huang S, Cheng X, Chen F (2023). Rational Design of Organelle-Targeted Fluorescent Probes: Insights from Artificial Intelligence. Research (Washington, DC).

[166] Wu M, Shu J (2018). Multimodal Molecular Imaging: Current Status and Future Directions. Contrast Media & Molecular Imaging.

[167] Wang T, Peng W, Du M, Chen Z (2023). Immunogenic sonodynamic therapy for inducing immunogenic cell death and activating antitumor immunity. Frontiers in oncology.

[168] Yang Y, Huang J, Liu M, Qiu Y, Chen Q, Zhao T (2023). Emerging Sonodynamic Therapy-Based Nanomedicines for Cancer Immunotherapy. Advanced science (Weinheim, Baden-Wurttemberg, Germany).

[169] Liang Y, Shao H, Liu K, Cao Q, Zhang S, Wang H (2025). Ultrasound-responsive phosphorescence in aqueous solution enabled by microscale rigid framework engineering of carbon nanodots. Light: Science & Applications.

