# Supplementary material for: Ultrasound-induced luminescence imaging for deep tissues
Source: Theranostics. 2026 Sep 11;16(16):9221–60. doi: 10.7150/thno.133393 (PMC13684393; doi:10.7150/thno.133393)
Supplement: Supplementary file 1 [file thnov16p9221s1.pdf]

Table S1

Table S1. Overview of the organization and key contents of this review.

Major topic	Scope and key focus
Basic introduction of ultrasound-induced luminescence	Advantages, development history, and conceptual basis of ultrasound-induced luminescence
Paradigms and mechanisms of ultrasound-induced luminescence	Instant ultrasound-induced luminescence, sonoafterglow luminescence, Off-On switching luminescence and their energy-conversion mechanisms
Molecular design in available materials for ultrasound-induced luminescence	Inorganic, organic, and hybrid systems across instant ultrasound-induced luminescence, sonoafterglow luminescence, and Off-On switching luminescence
Luminescent performance enhancement strategies (structure–property relationships for energy pathways)	Four key steps in the energy-conversion pathway: (Stage 1) the excitation input stage, (Stage 2) the energy capture stage, (Stage 3) the energy transfer and transformation stage, and (Stage 4) the emission stage where radiative and nonradiative pathways compete
Biological performance optimization	Biocompatibility, stability, targeting, and metabolism
Biomedical applications in various disease models	Tumor microenvironment, immune system, nervous system, and other biomedical regions
Challenges and perspectives	Fundamental bottlenecks, unresolved controversies, practical limitations, translational barriers and clinical readiness, and future directions for advanced probes and precision theranostics

Table S2

Table S2. Comparative performance matrix of representative system directions relevant to ultrasound-induced luminescence.

Representative system	λ_{em} (nm)	US	Penetration depth		Half-life (life/duration)	SNR	s.c.		i.v.		Imaging and therapeutic efficacy	Ref.
			Through tissue	*In vivo* (model)			Intensity per mass concentration	SBR	Intensity per dose per mouse	TBR		
mSZ-Clr@ePDA NP ($SrAl_2O_4:Eu^{2+}/ZnGa_2O_4:Cr^{3+}$ nanodots)	700	1MHz, 2 W cm^{-2}	N.A.	N.A.	N.A. (life≈ 4 h)	N.A.	N.A.	N.A.	N.A.	N.A.	US-propelled motion & afterglow for precise imaging and efficient eradication of *H. pylori*	2022
NCBS/DPAs SNAP	780	1MHz, 2 W cm^{-2}	4 cm	N.A.	110 s	N.A.	2.3×10^5 $(p/s/cm^2/sr)/(\mu g/mL)$	2255 (Balb/c mice)	1.1×10^4 $(p/s/cm^2/sr)/\mu g$	114 (Balb/c mice) [*Note*]	Modular sonoafterglow for deep-tissue background-free *in vivo* optical imaging	2022
NCBS/AMPA SNAP	500	1MHz, 2 W cm^{-2}	N.A.	N.A.	75 s	N.A.	9.5×10^5 $(p/s/cm^2/sr)/(\mu g/mL)$	9467 (Balb/c mice)	N.A.	N.A.		
Q SNAP (NCBS/DPAs)	780	1MHz, 1.5 W cm^{-2}	4 cm		≈ 1.5 min (life>5 min)	N.A.	N.A.	N.A.	N.A.	58.3 (Balb/c mice) [SBR]	Activatable sonoafterglow nanoprobes for T-cell imaging	2023
PNCL (PpIX/phenoxy-dioxetane precursor containing a dicyanomethyl chromone acceptor scaffold)	710	1MHz, 1.5 W cm^{-2}	20 mm	4T1	N.A. (life>250 min) (i.t.)	N.A.	28 $(p/s/cm^2/sr)/\mu M$ (i.t.)	N.A.	N.A.	N.A.	US-activated NIR chemiluminescence for deep tissue and tumor foci imaging	2023
Janus mSZ@PDA-NO NPs	696	1MHz, 2 W cm^{-2}	N.A.	N.A.	N.A. (life≈ 4 h)	N.A.	N.A.	N.A.	N.A.	N.A.	US-chargeable persistent luminescence for self-	2023

											propelled photothermal/NO Therapy	
Lipo@IR780/L012	470	FUS (1.5 MHz, puls 100 ms on, 900 ms off, 1 Hz, 2.2 MPa)	10 mm	Motor cortex After i.v.	N.A.	N.A.	N.A.	N.A.	N.A.	N.A.	US-triggered *in situ* photon emission for noninvasive optogenetics	2023
Lipo@IR780/L012/CaO_2	470	FUS (1.5 MHz, 1.55 MPa, pulse 100 on 900 ms off)	10 mm	Superficial motor cortex and deep ventral tegmental area after intracranial injection	N.A.	N.A.	N.A.	N.A.	N.A.	N.A.	Minimally invasive deep brain stimulation for behavioral control in animals *via* a flexible, mechanoluminescent sono-optogenetic system	2023
TD NPs	625–650	1MHz, 2 W cm^{-2}	2.2 cm	N.A.	180 s (life>7 h)	206 *in vivo*	N.A.	N.A.	N.A.	N.A.	Advance in energy-conversion for sonoafterglow imaging	2024
DPA-H_2S	710	1MHz, 1.5 Wcm^{-2}	20 mm	MET-induced liver injury	3.2 min (life 6 min)	N.A.	N.A.	N.A.	N.A.	4.5 (Balb/c-nude mice) [SBR]	Dual-locked probe with activatable sonoafterglow for precise imaging of liver injury	2024

NPs-Ce4-SN	~1100	2.5 W cm^{-2}	2 cm	subcutaneous MC38 tumor model	≈ 10 s	N.A.	N.A.	N.A.	N.A.	N.A.	NIR-II sonoafterglow for precise cancer imaging	2024
BTB-OC9/BDTSe	727	50 kHz, 5.6 W cm^{-2}	3 mm	N.A.	N.A. (life ≈ 174.83ms)	N.A.	9.15 ± 1.32 × 10^4 p s^{-1}cm^{-2}sr^{-1}	24	N.A.	N.A.	N.A.	2024
FURIN ($Zn_2Ga_{2.96}Ge_{0.75}O_8:Cr_{0.02},Nd_{0.02}$/MSN)	694, 883, 1062, 1335 (five repetitions of LED and FUS)	Pre-charged by 635 nm light + pulsed FUS (1 MHz, 34.1 W cm^{-2})	N.A.	Footpad of the right forelimb	N.A.	N.A.	N.A.	N.A.	N.A.	N.A.	FUS-responsive persistent mechanoluminescence for spatiotemporal immune activation control in lymph nodes	2024
FUSION ($Zn_{1.2}Ga_{1.6}Sn_{0.2}O_4$:0.5%$Cr^{3+}$/MSN/R848/DPPC)	694	Pre-charged by 635nm light + pulsed FUS (1 MHz, 33.9 Wcm^{-2})	N.A.	4T1	N.A.	8.5 (first ML) 0.2 (after 20 times FUS) (i.v.)	N.A.	N.A.	N.A.	N.A.	FUS-responsive persistent mechanoluminescence for reporting the effectiveness of macrophage regulation	2024

CSAPs (HMME/ MACF)	≈ 700	30 kHz, 2 W cm^{-2}	1.8 cm (*in vivo* after subcutaneous injection)	Glioblastoma	8 min (life≈ 30 min)	N.A.	N.A.	24.11	N.A.	N.A.	Intratumoral bacterial-targeted cascade-responsive sonoafterglow nanoprobes for immune reprogramming and visualization in glioblastoma	2025
SPNs (SP1) @CoOOH	570-650	1 MHz, 0.7-1 W cm^{-2} (*in vitro*) 2.2 W cm^{-2} (*in vivo*)	1.2 cm	4T1	N.A. 1 min (imaging) 10 min (therapy)	N.A.	N.A.	N.A.	N.A.	N.A.	CoOOH universal approach for enhanced SDT efficiency and ultrasound-activated luminescence	2025
ncRuPA$_{APN}$	560 640	1 MHz, 2 W cm^{-2}	N.A.	4T1	5 s	N.A.	N.A.	N.A.	≈ 1600 (p/s/cm^2/sr)/μM	2.7 (Balb/c mice) (i.t.)	Sonoafterglow luminophore with a nonconjugated linker for tumor-specific Theranostics	2026
FZ970 (ZnPc/FZA)	970	1 MHz, 1.6 W cm^{-2}	4 cm	N.A.	≈ 150 s	N.A.	N.A.	N.A.	N.A.	N.A.	Ultrasound/peroxynitrite-synergized NIR-II afterglow-guided SDT	2026
TCL NPs (TTQ/ CL─COOCH3/DHF)	N.A.	1 MHz, 1.5 W cm^{-2}	2 cm	CT26	N.A. (life>27 min *in vivo*)	1180 (peak at 3	N.A.	N.A.	N.A.	N.A.	High-Contrast Imaging and Synergistic Gas-SDT of Hypoxic Tumors	2026

						min)						
3SCe-NP	710	20 Hz, 1.5 W cm^{-2}	7 cm	subcutaneous colon, orthotopic tumor (colon/ breast/pancreatic/ lung/glioma) tumor, peritoneal metastasis, lymph nodes	≈ 40 min	N.A.	N.A.	330	N.A.	27-39 (peritoneal metastasis) (SBR)	A self-activating cyclic amplification NIR sonoafterglow probe for high-contrast imaging *in vivo*	2026
PpIX-M (PpIX/TPP^{+})	600–700	1 MHz, 1.5 W cm^{-2}	N.A.	N.A.	N.A. (life>20 min *in vivo*)	N.A.	≈266 (p/s/cm^2/sr)/(μg/mL)	48.6 (nude mice)	N.A.	N.A.	Sonofterglow-guided SDT	2026
FTA NPs	600-710	1 MHz, 2.2 W cm^{-2}	5 cm	Pancreatic tumor model	N.A. (life>15 min)	N.A.	N.A.	N.A.	N.A.	N.A.	Fractionated ultra-bright sonofterglow endoperoxide SDT	2026

Abbreviations: λem, emission wavelength; s.c., subcutaneous injection; i.v., intravenous injection; CNDs, carbon nanodots; FUS, focused ultrasound; SDT, sonodynamic therapy; NIR-II, near-infrared-II.

[*Note*] Balb/c mice have higher background (~5×10^3 p/s/cm^2/sr) than nude mice (~2×10^3 p/s/cm^2/sr).
